# A Phase II Study of Perioperative Avelumab plus Chemotherapy for Patients with Resectable Gastric Cancer or Gastroesophageal Junction Cancer – The MONEO Study

**DOI:** 10.1158/1078-0432.CCR-25-0369

**Published:** 2025-05-19

**Authors:** Maria Alsina, Guillermo Villacampa, Carlos de Andrea, Ana Vivancos, Mariano Ponz-Sarvise, Virginia Arrazubi, Paula Jimenez-Fonseca, Marc Diez, Enrique Sanz-Garcia, Eva Martínez, Raquel Guardeño, Mariona Calvo, Cristina Bugés, Federico Longo, Víctor Navarro, Eduardo García-Galea, Alena Gros, Maria C. Ochoa, Alvaro Lopez-Janeiro, Sandra Sanchez-Gregorio, Claudia Herrero, Ibone Labiano, Maria Vila-Casadesús, Dario López, Raluca Alexandru, Susana Muñoz, Josep Tabernero, Ignacio Melero

**Affiliations:** 1 Vall d’Hebron Institute of Oncology (VHIO), Barcelona, Spain.; 2 Medical Oncology Department, Hospital Universitario de Navarra, Pamplona, Spain.; 3 Translational Medical Oncology Unit, Navarrabiomed – IdiSNA, Pamplona, Spain.; 4 Statistics Unit, Vall d’Hebron Institute of Oncology (VHIO), Barcelona, Spain.; 5 Clínica Universidad de Navarra, University of Navarra, Centro de Investigación Biomédica en Red de Cáncer (CIBERONC), Pamplona, Spain.; 6 Cancer Genomics, Vall d’Hebron Institute of Oncology (VHIO), Barcelona, Spain.; 7 Department of Medical Oncology and Program in Solid Tumors, CIMA-IdiSNA-Universidad de Navarra, Cancer Center Clínica Universidad de Navarra (CCUN), Pamplona, Spain.; 8 Medical Oncology Department, Hospital Universitario Central de Asturias, ISPA, Oviedo, Spain.; 9 Medical Oncology Department, Institut Catala d'Oncologia-IDIBELL, L’Hospitalet, Barcelona, Spain.; 10 Medical Oncology Department, HM Sanchinarro Centro Oncologico Clara Campa, Madrid, Spain.; 11 Princess Margaret Cancer Centre, University Health Network, Toronto, Canada.; 12 Medical Oncology Department, Hospital Universitario Marqués de Valdecilla, Instituto de investigación IDIVAL, Santander, Spain.; 13 Medical Oncology Department, Institut Català d'Oncologia, Hospital Josep Trueta, Girona, Spain.; 14 Medical Oncology Department, Institut Català d’Oncologia (ICO), Badalona, Hospital Germans Trias i Pujol, Barcelona. Spain.; 15 Medical Oncology Department Hospital Universitario Ramón y Cajal, Universidad de Alcalá, IRICYS, CIBERONC, Madrid, Spain.; 16 Vall d’Hebron Barcelona Hospital Campus, Barcelona, Spain.; 17 Centro de Investigación Biomédica en Red de Cáncer, Clínica Universidad de Navarra, University of Navarra, Pamplona, Spain.; 18 Program of Immunology and Immunotherapy, Center for Applied Medical Research (CIMA), Pamplona, Spain.; 19 Clínica Universidad de Navarra and CIMA, Pamplona, Spain.; 20 IDISNA and CIBERONC, Pamplona, Spain.; 21 Nuffield Department of Medicine, University of Oxford, Oxford, United Kingdom.

## Abstract

**Purpose:**

Immune checkpoint inhibitors combined with chemotherapy have provided successful results in patients with gastric and gastroesophageal junction (G/GEJ) cancers in the metastatic setting. Similar strategies have been explored in earlier stages. In this study, we present the final results of the phase II MONEO trial, which evaluated the addition of avelumab to neoadjuvant chemotherapy.

**Patients and Methods:**

Patients with untreated, resectable G/GEJ adenocarcinoma received neoadjuvant treatment with four cycles of avelumab plus the FLOT4 regimen, followed by surgery. Upon postoperative recovery, patients underwent four additional adjuvant cycles of the same combination, followed by avelumab monotherapy for up to 1 year. The primary endpoint was pathologic complete response rate. Sequential flow cytometry and cytokine determination were performed in peripheral blood, along with multiplex tissue immunofluorescence and RNA sequencing in tumor specimens.

**Results:**

Forty patients were enrolled, achieving a pathologic complete response rate of 21.1% (95% confidence interval, 10.0–37.0). The major pathologic response rate was 28.9%, more pronounced in patients with tumors expressing PD-L1 before treatment as measured by the combined positive score (cutoff, 10; 33.3% vs. 21.1%). The results propose several potential biomarkers considering tumor immune infiltrate, circulating immune cells, and cytokines. Eighty percent of patients experienced treatment-related grade ≥3 adverse events.

**Conclusions:**

The combination of avelumab plus the FLOT4 regimen showed relatively modest efficacy in resectable G/GEJ adenocarcinoma. Better results were observed in PD-L1 combined positive score ≥10% tumors. Exploratory biomarker analyses provide insights that may help to identify candidates most likely to benefit from chemoimmunotherapy as a neoadjuvant treatment.

**Translational Relevance**

The phase II MONEO trial was designed to better understand the immunobiologic pathways underlying the response to the combination of immune checkpoint inhibitors and chemotherapy in the neoadjuvant setting of gastric cancer. Patients with locally advanced, resectable gastric and gastroesophageal junction cancers were treated with a perioperative approach using the anti–PD-L1 antibody avelumab and the FLOT4 schema. The primary endpoint of the study was the rate of pathologic complete response. A close pharmacodynamic follow-up was performed to investigate potential immune biomarkers in both tissue and blood. The combination of avelumab and chemotherapy was safe and showed signs of relatively moderate efficacy. Exploratory analyses suggested a numerical correlation between the grade of pathologic response and the PD-L1 combined positive score. Additionally, this study proposes some tumor- and blood-based parameters with potential predictive value for pathologic response, warranting prospective validation.

## Introduction

Gastric and gastroesophageal junction (G/GEJ) cancers represent the fifth most common solid malignancy and the fourth leading cause of cancer-related mortality worldwide (1). Radical surgery remains the gold standard of the curative approach for patients with resectable tumors, yet less than 25% of newly diagnosed patients can be considered for resection (2). Neoadjuvant and perioperative strategies with chemotherapy aim to improve survival outcomes, but prognosis remains poor (2). In 2017, the fluorouracil–leucovorin–oxaliplatin–docetaxel (FLOT4) phase 2/3 clinical trial established the current standard-of-care in Western patients with G/GEJ cancer (3).

Efforts to explore synergistic approaches along with the FLOT chemotherapy regimen have been carried out during the last decades, considering the addition of targeted therapies and immune checkpoint inhibitors, accounting with the recognized inherent molecular heterogeneity of these tumors (4). The addition of antiangiogenic agents did not translate into better survival outcomes in unselected populations (5, 6). In contrast, the addition of anti-HER2 antibodies in patients harboring HER2-positive G/GEJ cancers showed a good therapeutic synergism, with higher rates of pathologic responses (7, 8). Finally, the combinations with immune checkpoint inhibitors have been tested during the last decade, with contradictory results among studies (9–11). PD-L1, an inhibitory checkpoint molecule, has been shown to be expressed in gastric cancer (12). Randomized trials have shown improved clinical outcomes by adding PD-1 antibodies to chemotherapy in patients with metastatic G/GEJ cancers harboring high PD-L1 combined positive score (CPS) levels (13, 14), leading to the approval of these regimens in the first-line setting for metastatic disease. Furthermore, 1 year of treatment with PD-1 inhibition in patients with esophageal and GEJ cancers following neoadjuvant chemoradiotherapy and surgery improved rates of disease-free survival [overall survival (OS) results are still awaited; ref. 15]. Overall, these studies demonstrated the efficacy of anti–PD-1 strategies in G/GEJ cancer, with moderate association with PD-L1 CPS expression, thus indicating an unmet need for the identification of predictive biomarkers.

The phase II MONEO clinical trial was designed to explore a potential synergism between the anti–PD-L1 antibody avelumab with the use of perioperative chemotherapy in patients with resectable stage II and III G/GEJ cancer. The primary endpoint was the rate of pathologic complete response (pCR). Avelumab is a human Fc receptor–competent fully human IgG1 that in addition to PD-L1/PD-1 blockade may exert some Fc-mediated proinflammatory properties in the tumor tissue microenvironment (16). In this study, we present the results of the study, including comprehensive translational analyses, to identify predictive biomarkers and gain immunobiologic insights.

## Patients and Methods

### Study design and population

The MONEO study was a multicenter, single-arm, phase II trial conducted at 10 sites in Spain. Eligible patients were treatment-naïve adults (≥18 years) with histologically confirmed stage Ib (T1N1 only) to IIIC G/GEJ cancer (American Joint Committee on Cancer 7th Edition) amenable to surgery, with evaluable disease per RECIST version 1.1 criteria, an Eastern Cooperative Oncology Group (ECOG) performance status of 0 to 1, and adequate organ function. An available paraffin block from diagnostic biopsy and surgery was required. Key exclusion criteria included unresectable disease, prior cancer therapy, significant comorbidities, and active autoimmune diseases. Detailed inclusion criteria can be found in the study protocol (Supplementary). The trial, registered at ClinicalTrials.gov (NCT03979131), was approved by the regulatory authorities and conducted in accordance with the Helsinki Declaration, International Council for Harmonisation of Good Clinical Practice (ICH-GCP) guidelines or International Organization for Standardization 14155 standards, and local regulations. All patients provided written informed consent.

### Treatment

Patients received a neoadjuvant regimen prior to surgery, consisting of four cycles of avelumab (10 mg/Kg i.v. day 1 once every 2 weeks) with the standard dose of FLOT chemotherapy (5-fluorouracil 2,600 mg/m^2^ 24 hours i.v. day 1, leucovorin 200 mg/m^2^ i.v. day 1, oxaliplatin 85 mg/m^2^ i.v. day 1, and docetaxel 50 mg/m^2^ i.v. day 1, once every 2 weeks). After surgery, patients underwent four cycles of adjuvant therapy using the same regimen, followed by up to 20 cycles of avelumab monotherapy (10 mg/Kg i.v. once every 2 weeks). The overall treatment duration for each participant included preoperative therapy, surgical intervention, and postoperative therapy. Tumor assessments, CT scans, or MRI were performed at baseline, after neoadjuvant therapy, after adjuvant therapy, and subsequently every 6 months up to 5 years. Avelumab discontinuation criteria included confirmed disease progression (RECIST version 1.1), significant clinical deterioration or clinical progression, unacceptable toxicity, or consent withdrawal.

### Study endpoints

The primary endpoint was the pCR rate at surgery, locally assessed and consensual according to Becker remission criteria; i.e., defined as the absence of residual tumor based on evaluation of the resected esophagogastric specimen (Becker grade 1a; ref. 17). Secondary endpoints included the following: (i) major pathologic response (MPR) rate (Becker remission criteria grades 1a and 1b); (ii) surgical complete resection, (iii) overall response rate (ORR), (iv) progression-free survival (PFS), and (v) OS. The ORR was defined as the proportion of all subjects achieving complete or partial response according to RECIST version 1.1 by a local investigator. PFS was defined as the time from the initial date of neoadjuvant chemotherapy to the date of the first documented disease progression or death from any cause, whichever occurred first. Patients with progressive disease during the neoadjuvant phase were also included. OS was defined as the time from first treatment administration to the date of death due to any cause. Safety endpoints included adverse events (AE) graded using the Common Terminology Criteria for Adverse Events, version 4.0.

### Biomarker analyses

Baseline tumor biopsies were obtained for the assessment of PD-L1 CPS, HER2, and mismatch-repair (MMR) system status in the intention-to-treat (ITT) population. Baseline tumor and tissue specimens were assessed to compare the tumor immune infiltrates among patients who underwent surgery (multiparametric IHC). Basal and postneoadjuvant treatment (after cycle 4) blood samples were obtained from 12 patients to perform circulating immune cell characterization (multiparametric flow cytometry). Additionally, baseline, post–cycle 1, and post–cycle 4 blood samples were obtained to assess cytokine concentrations in 36 patients (ProcartaPlex Kit using Luminex technology). Gene expression profiles were assessed in surgical specimens in 22 patients (VHIOε00, Epsilon). Detailed procedures are specified in Supplementary Methodology S1.

Tissue and blood biomarker analyses were centrally assessed either in the Pathology Department of Clínica Universitaria de Navarra, Pamplona (PD-L1 CPS, HER2, and MMR assessment, tumor immune infiltrates, circulating immune cell characterization, and cytokine concentrations), and in the Cancer Genomics Department of Vall d’Hebron University Institute, Barcelona (gene expression profiles).

### Statistical analysis

The study aimed to improve pCR rates with the addition of avelumab to the FLOT regimen compared with the historic control in the ITT population. The pCR rate of the historic data was estimated at 16% (based on the FLOT4 study; ref. 3), and the pCR rate in the study treatment was assumed to be 33%. The study was designed to achieve >80% statistical power with a one-sided type I error of 0.1 using a single-stage design. After accounting for the forecasted dropout rate, the target sample size was at least 37 patients. Due to the restrictions secondary to the COVID pandemic, two patients were unable to undergone surgery, thus causing two major protocol violations of unrelated study procedures. To overcome this issue, two populations were defined for this study: (i) the initially defined ITT population, which included all patients enrolled in the study, and (ii) the modified ITT (mITT) population, excluding the two aforementioned patients. Patients with progressive disease during the neoadjuvant treatment or those who stopped treatment due to toxicity were also included in the mITT population as nonresponders. For binary and categorical efficacy endpoints (i.e., pCR and ORR), counts and percentages, with 95% confidence intervals (CI), were calculated. The Kaplan–Meier method was used to estimate survival rates, and univariable Cox proportional hazard models were used to obtain HRs with 95% CIs in exploratory subgroup analyses. For the biomarker analysis, differential expression analysis was used, and the nonparametric Mann–Whitney U test was used to compare responders and nonresponders. No data imputation was performed. The median follow-up was estimated using the reverse Kaplan–Meier method. All statistical analyses were conducted using R version 4.3.1 software.

### Data availability

The data produced in this study are not publicly accessible due to concerns regarding patient privacy or consent. However, they are available upon reasonable request from the corresponding author.

## Results

Between August 2019 and February 2021, 46 patients were screened, and 40 were included in the study. All patients received the study treatment; however, two patients were unable to adhere to the treatment protocol due to the COVID-19 pandemics restrictions and were excluded from the mITT population (*n* = 38). Five more patients did not undergo surgery (Supplementary Fig. S1). The reasons for not being resected were peritoneal progression objectified during the surgical approach (three patients), pulmonary progression objectified in the presurgery CT scan (one patient), and limiting toxicity with early progression (one patient). Baseline characteristics are summarized in Table 1. Briefly, the median age was 64 years, 55% had an ECOG score of 1, 60% had gastric cancer, and 51.3% had stage II disease (Supplementary Table S1). MMR status information was available for 35 patients; with 29 classified as MMR-proficient and six as MMR-deficient (dMMR). At the data cutoff (November 2023), the median duration of follow-up was 37.7 months.

**Table 1.** Baseline patient characteristics in the ITT and mITT populations.

Characteristics	ITT (*n* = 40)	mITT (*n* = 38)
Age, median (IQR)	64 (56–70)	65 (57–71)
BMI, median (IQR)	27.2 (24.4–28.8)	27.6 (24.7–29.0)
Ethnicity		
Hispanic	5 (12.5%)	4 (10.5%)
Not Hispanic	35 (87.5%)	34 (89.5%)
Sex		
Male	22 (55.0%)	21 (55.3%)
Female	18 (45.0%)	17 (44.7%)
ECOG		
0	18 (45.0%)	17 (44.7%)
1	22 (55.0%)	21 (55.3%)
Location		
Gastric	24 (60.0%)	23 (60.5%)
Gastro-esophageal	16 (40.0%)	15 (39.5%)
Histologic type (Lauren)		
Diffuse	18 (45.0%)	17 (44.7%)
Intestinal	15 (37.5%)	14 (36.8%)
Mixed	3 (7.5%)	3 (7.9%)
Indeterminate	2 (5.0%)	2 (5.3%)
Not defined	2 (5.0%)	2 (5.3%)
Stage at diagnosis		
Stage II	20 (51.3%)	19 (51.4%)
Stage III	19 (48.7%)	18 (48.6%)
Missing	1	1
T		
T2	1 (2.5%)	1 (2.6%)
T3	28 (70.0%)	26 (68.5%)
T4a	10 (25.0%)	10 (26.3%)
Any T	1 (2.5%)	1 (2.6%)
N		
N0	13 (32.5%)	13 (34.2%)
N1	15 (37.5%)	14 (36.9%)
N2	10 (25%)	9 (23.7%)
N3	1 (2.5%)	1 (2.6%)
Any N	1 (2.5%)	1 (2.6%)
MMR status		
dMMR	6 (15%)	6 (16%)
pMMR	29 (73%)	29 (76%)
Not known	5 (12%)	3 (8%)

### Efficacy endpoints

**Figure 1.**
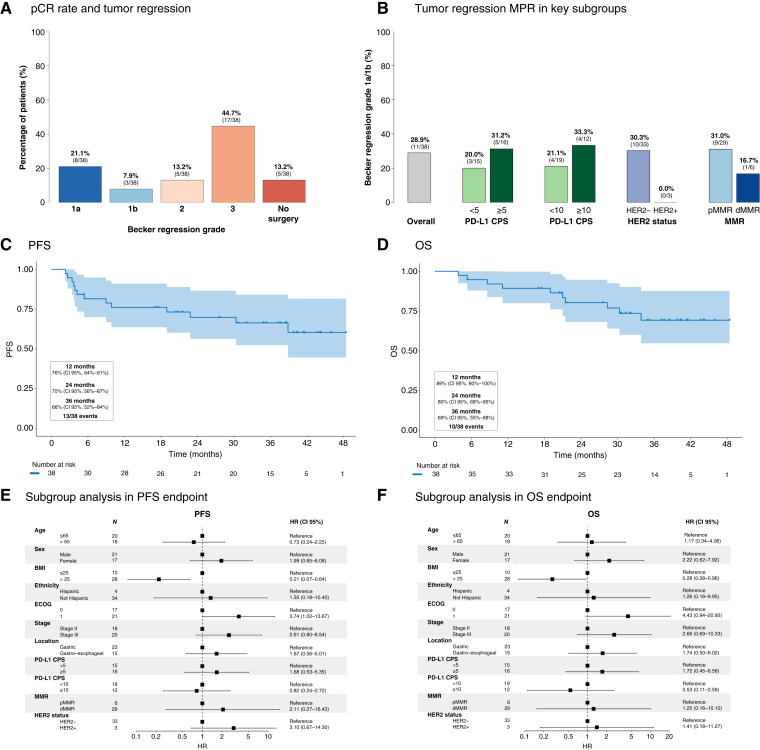
Efficacy endpoints. **A,** Percentage of tumor regression grade. **B,** Tumor regression grade depending on PD-L1 CPS, HER2, and MMR status. **C,** PFS. **D,** OS. **E,** Subgroup analysis for PFS. **F,** Subgroup analysis for OS. pMMR, MMR-proficient.

The primary end point of the pCR rate at surgery was 21.1% (8/38; 95% CI, 10.0–37.0) in the mITT population and 20.0% (8/40; 95% CI, 9.0–36.0) in the ITT population, showing no statistically significant improvement over the historic control, as the lower limit of the CI exceeded the 16% threshold (*P* value = 0.26; Fig. 1A; ref. 3). The MPR rate was 28.9% in the mITT population, which was numerically associated with PD-L1 CPS, demonstrating a higher percentage of MPR in patients with elevated PD-L1 CPS using the cutoffs of 5 (31.2% vs. 20.0%) and 10 (33.3% vs. 21.1%; Fig. 1B; Supplementary Table S2). The percentage of MPR was 16.7% (1/6) in dMMR tumors and nonexistent in the three cases of HER2-positive tumors (0/3; Fig. 1B). Up to 25 patients had measurable disease; four of them presented complete response, and 12 had partial response. Overall, the ORR by RECIST version 1.1 was 42.1% (95% CI, 26.7–59.1), including 10.5% complete and 31.6% partial responses.

In terms of survival outcomes, the 3-year PFS rate was 66% (95% CI, 52–84), whereas the 3-year OS was 69% (95% CI, 55–88; Fig. 1C and D). Subgroup analyses of PFS and OS showed consistent results across subgroups, except for body mass index (BMI) and ECOG. Notably, patients with high BMI and an ECOG score of 0 experienced better survival outcomes compared with those with low BMI and ECOG score ≥1, respectively. Patients who presented pCR and MPR tend to associate better survival outcomes (Supplementary Table S3). No difference in long-term outcomes was found regarding PD-L1 CPS expression (Fig. 1E and F).

### Biomarker analysis

**Figure 2.**
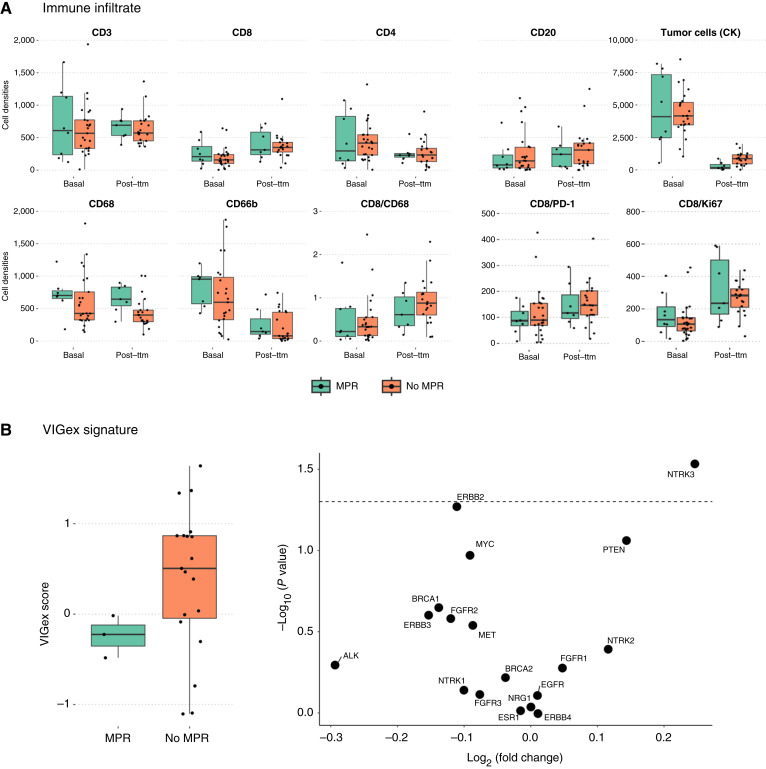
Tissue biomarker analyses and the VIGex score. **A,** Immune tumor contexture of the basal samples and surgical specimens in patients with MPR (*n* = 8) and without MPR (*n* = 25). **B,** Gene expression profile on surgical specimens, integrated within the VIGex score, and individual gene expression profile on surgical specimens. CK, cytokeratin; Post-ttm, posttreatment.

The immune infiltrate of tumors was compared at baseline and after neoadjuvant treatment in patients with MPR (*n* = 8) and without MPR (*n* = 25). No statistical differences were observed in CD3, CD4, and CD8 lymphocytes, neither in activated CD8 lymphocytes (CD8^+^PD-1^+^ and CD8^+^Ki67^+^). Neutrophils (CD66b) and tumor cells (cytokeratin) significantly decreased in posttreatment samples compared with pretreatment samples (Fig. 2A). When we evaluated whether neoadjuvant treatment induced the formation of tertiary lymphoid structures in all posttreatment resections, neither the presence nor the abundance of tertiary lymphoid structures was associated with MPR (Supplementary Fig. S2).

Transcriptomic analyses were performed on the surgical specimens (*n* = 22, including three patients with MPR). The VIGex signature denoting evidence for cellular immune responses (18) was not associated with response at surgery. However, patients with MPR showed higher expression levels of *NTRK3* mRNAs and tended to present lower levels of *HER2* transcripts (Fig. 2B).

**Figure 3.**
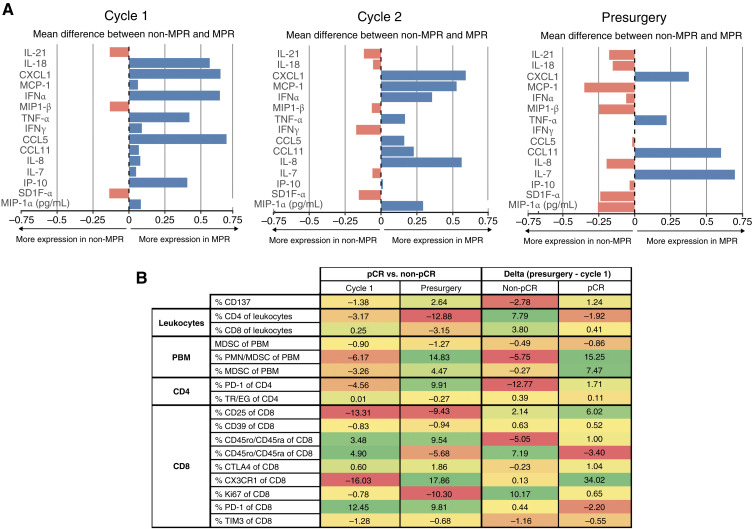
Analyses on circulating cytokines and leukocytes. **A,** Mean difference in peripheral cytokines (fold change) at cycle 1, cycle 2, and at presurgery evaluation between patients with MPR (*n* = 11) and without MPR (*n* = 25). **B,** Peripheral immune cells quantified as percentages and compared baseline and posttreatment results showing the difference between pCR (*n* = 3) and non-pCR (*n* = 8) patients, with the delta in patients with pCR as indicated. Positive (green) or negative (red) differences are color-coded, and the magnitude is reflected by the intensity of the color. MDSC, myeloid-derived suppressor cell; PBM, peripheral blood mononuclear; PMN, polymorphonuclear; TR/EG, regulatory T cells.

Analyses of circulating cytokines revealed increased IFNα concentration in serum at baseline in patients with MPR (*n* = 11) compared with the others (*n* = 25), whereas chemokine ligand 1 (CXCL1) levels remained consistently higher in responder patients across all three time points (Fig. 3A). Finally, we conducted a peripheral blood analysis by multicolor flow cytometry, which revealed that patients with pCR (*n* = 3) typically exhibit a higher baseline level of circulating PD-1^+^CD8^+^ cells as compared with non-pCR patients (*n* = 8). However, the dynamics of immune cell populations before and after neoadjuvant treatment were found to be similar between the two groups, except for CX3CR1^+^ CD8 cells, which were increased among patients with pCR (Fig. 3B).

### Safety

The most common AEs (any grade) were fatigue, neutropenia, and diarrhea. Grade ≥3 AEs occurred in 80% of patients, mainly primarily neutropenia (52.5%), fatigue (25.0%), and nausea/vomiting (12.5%). Grade ≥3 AEs related to avelumab were reported in 25.0% (10/40). The most common AEs of special interest for avelumab were of grade 1 to 2, mainly diarrhea (35%) and transaminase elevation (15%). No treatment-related deaths occurred up to the data cutoff. Overall, 95.0% (38/40) of patients completed avelumab in the neoadjuvant phase, whereas 37.5% (15/40) completed adjuvant avelumab. Dose reductions in chemotherapy were required for five patients during the neoadjuvant phase and eight patients during the adjuvant phase. A complete summary of the incidence of AEs can be found in Supplementary Table S4.

## Discussion

The MONEO trial evaluated the combination of neoadjuvant avelumab plus chemotherapy in 40 patients with locally advanced G/GEJ cancer. The primary endpoint of this study was not met, with a pCR rate of 21.1%. The safety profile was manageable, and compliance rates were as expected. Five patients could not proceed to surgery due to progression, three of them with peritoneal progression objectified at the surgery time. As diagnostic laparoscopy was not mandatory for initial staging, we presume that the three nonresected patients already had peritoneal spread since the beginning of the treatment.

Rates of pCR after neoadjuvant treatment vary across different clinical trials testing perioperative chemoimmunotherapy, ranging from 13% to 45% (9–11, 19–22). The international randomized phase 3 KEYNOTE-585 trial (9) evaluated the addition of the anti–PD-1 agent pembrolizumab to perioperative chemotherapy and showed improved pCR rates when compared with placebo plus chemotherapy (13% vs. 2%, respectively). However, this level of presurgical efficacy was insufficient to translate into better survival outcomes. The phase 3 MATTERHORN trial (11), which explored the combination of the anti–PD-L1 agent durvalumab with FLOT, showed a significant improvement in the pCR rate compared with placebo plus chemotherapy (19% vs. 7%, respectively), which translated into better event-free survival outcomes, according to a recent press-release (23). Despite the initial assumption of strong correlation between pathologic response and survival (24), current data challenge pCR as the best surrogate of survival in gastric cancer. In this sense, the MONEO trial shows favorable survival times (3-year PFS of 66% and 3-year OS of 69%), in line with published reports (9). Furthermore, the observed pCR rate (21%) in the MONEO is comparable with rates reported in the larger global studies mentioned above, thus questioning whether the predefined threshold of 16% for pCR with FLOT alone might have been too high outside Germany.

Interestingly, the MONEO trial demonstrates numerically increased rates of pCR and MPR in patients harboring tumors with higher PD-L1 CPS expression levels, which is also in line with reported data from similar studies of neoadjuvant chemoimmunotherapy. The MATTERHORN phase 3 trial reported increased responses in patients with higher PD-L1 tumor area positivity–positive tumors (11). Likewise, the phase 2 DANTE trial, which evaluated the addition of the anti–PD-L1 agent atezolizumab to FLOT, also reported more frequent pCR and MPR rates in tumors with higher PD-L1 CPS (10), and an exploratory phase 2 study evaluating the combination of the anti–PD-1 agent camrelizumab and the tyrosine kinase inhibitor antiangiogenic agent apatinib with chemotherapy also showed similar results (22). Finally, the MONEO trial tested synergism with avelumab, a Fc-active PD-L1 antibody, which could inhibit the PD-L1/PD-1 blockade and promote the Fc-mediated proinflammatory response. To the best of our knowledge, this strategy has been only tested in another single-arm, phase II study in combination with a similar chemotherapy regimen which related comparable clinical results (14% of pCR; ref. 25).

In the MONEO trial, MMR or HER2 tumor status was not associated with different rates of pathologic response, probably due to the few dMMR (n = 8) and HER2-positive (*n* = 3) cases included. However, other studies evaluating either chemoimmunotherapy combinations or chemo-free immunotherapy combinations in the perioperative setting showed a substantial superior activity in dMMR tumors (9–11, 20, 22, 26, 27).

Tumor tissue analysis showed no substantial differences in T-cell content between MPR and non-MPR patients. This is in accordance with the available transcriptomic data but contrasts with reports across other cancer types (28), including gastric cancer, for unknown reasons that may be related to the nature chemoimmunotherapy treatment (29). The synergistic value of the chemimmune combinations relies on the depletion of myeloid immunosuppressive cells and lymphopenia, which may reduce regulatory T cells, thus making room for the proliferation of effector T cells. In this regard, whether FLOT is the best regimen to be combined with PD-(L)1 checkpoint inhibitors becomes debatable due to the immunosuppressive effects of fluoropyrimidine and taxane. Notwithstanding, both neutrophil and (as expected) tumor cell levels were significantly more decreased within responder patients, consistent with the literature.

Discovery of predictive biomarkers in the blood would allow for noninvasive approaches for selection of patients to preoperative immunotherapy. In this sense, different studies show the potential value of ctDNA regarding treatment selection and survival prediction in G/GEJ cancer and other gastrointestinal malignancies (19, 20). In contrast, less data exist about the predictive value of type of circulating immune cell subsets in G/GEJ cancer (30). Our analysis showed that those patients who achieved a pCR rate tend to present more circulating basal PD-1^+^CD8^+^ cells, which probably simply reflects an already ongoing antitumor immune response. When examining dynamics of the immune cells, we further found a tendency of an increase in CX3CR1^+^ CD8^+^ cells in those patients experiencing pathologic responses. Such a phenomenon has already been described in lung cancer (31).

Other potentially noninvasive biomarkers measurable in blood samples apply to serum cytokine concentration profiles. Our analysis identified higher concentrations of IFNα in patients who eventually respond but not in the case of other cytokines such as IFNγ, which had already been correlated with positive response to immunotherapy in G/GEJ cancer and other malignancies (19, 32). Also, we observed a more pronounced decrease of CXCL1 in those responding patients which is considered an immunosuppressive factor (33). This could be indicative of the fact that the main cells producing CXCL1 are malignant cells themselves (34). In this regard, CXCL1 is considered a pro-cancer chemokine that, among other functions, enriches the tumor microenvironment with neutrophils, macrophages, and neutrophil extracellular traps (35, 36). Of note, no other protumor cytokines increased in the circulation of unresponsive patients.

Finally, from the safety perspective, the incidence of AEs in MONEO was slightly higher than other comparable studies (9, 10), with 80% of patients experiencing grade ≥3 AEs and 25% of patients experiencing grade ≥3 AEs related to avelumab. Of note, avelumab is a Fc-competent antibody, a feature that might be advisable in the tumor tissue but leading to more frequent infusion reactions (37).

Limitations of this study include the relatively small sample size and single-arm design, which prevent drawing definitive conclusions on the clinical and exploratory endpoint findings.

In conclusion, our study shows that the combination of neoadjuvant avelumab plus FLOT chemotherapy is safe and has relatively modest antitumor activity in G/GEJ adenocarcinoma. pCR rates were higher in patients whose tumors showed higher expression of PD-L1 CPS. Exploratory biomarkers showed potential for tumor and blood parameters to help in the selection of patients who may derive benefit from chemoimmunotherapy as a neoadjuvant treatment. Prospective randomized clinical trials including treatment decision stratification based on tissue and blood criteria are needed to set up the validation of the proposed biomarkers.

## Supplementary Material

Supplementary Figure S1 Supplementary Figure 1. Study flowchart.

Supplementary Figure S2 Supplementary Figure 2: Tertiary lymphoid structures in surgical tumor samples, analysed separately depending on tumor response.

Supplementary Table S1 Supplementary Table 1: Representativeness of study participants

Supplementary Table S2 Supplementary Table 2: Rates of pathological complete response (pCR) and non-pCR according to PD-L1 CPS cutoffs levels of 5 and 10.

Supplementary Table S3 Supplementary Table 3: Surrogacy between pCR and MPR status with overall survival

Supplementary Table S4 Supplementary Table 4: Incidence of adverse events during the study treatment.

Supplementary Methodology S1 Supplementary Methodology

Supplementary Data S1 Study protocol
