# Supplementary figures and images for: A Phase II Study of Perioperative Avelumab plus Chemotherapy for Patients with Resectable Gastric Cancer or Gastroesophageal Junction Cancer – The MONEO Study
Source: Clin Cancer Res. 2025 May 19;31(14):2890–8. doi: 10.1158/1078-0432.CCR-25-0369 (PMC12260514; doi:10.1158/1078-0432.CCR-25-0369)

## Supplementary Figures

**Supplementary Figure 1.** Study flowchart.

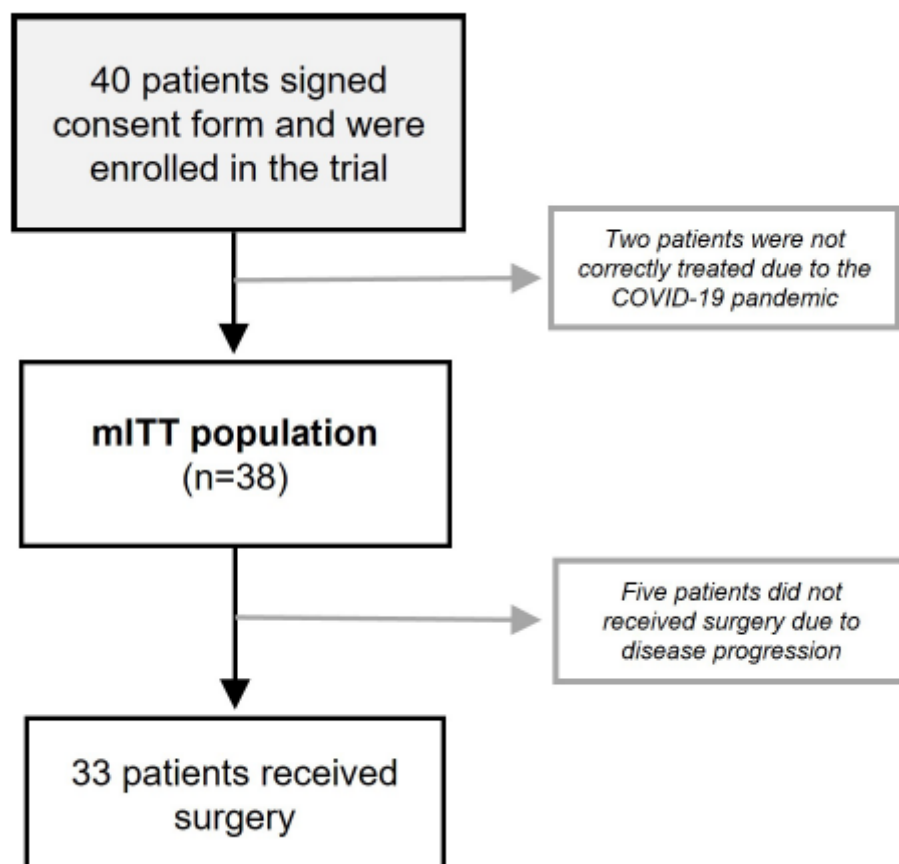

Supplement: Supplementary Figure S1 — Supplementary Figure 1. Study flowchart. [file ccr-25-0369_supplementary_figure_s1_suppfs1.pdf]
