# Supplementary material for: A Phase II Study of Perioperative Avelumab plus Chemotherapy for Patients with Resectable Gastric Cancer or Gastroesophageal Junction Cancer – The MONEO Study
Source: Clin Cancer Res. 2025 May 19;31(14):2890–8. doi: 10.1158/1078-0432.CCR-25-0369 (PMC12260514; doi:10.1158/1078-0432.CCR-25-0369)

Supplementary Figure 2: Tertiary lymphoid structures in surgical tumor samples, analysed separately depending on tumor response.

TLS analysis

PUBLISHED
April 2, 2024

	1a or 1b Response				
**Characteristic**	**Non-responder**, N = 21[1]	**Responder**, N = 7[1]	**OR**[2]	**95% CI**[2]	**p-value**
**TLS Presence**					
NO	2 (9.5%)	3 (43%)	—	—	
YES	19 (90%)	4 (57%)	0.14	0.01, 1.10	0.065
**Number of TLS**	5.0 (4.0, 9.0)	2.0 (0.0, 9.0)	0.92	0.75, 1.08	0.4

[1] n (%); Median (IQR)

[2] OR = Odds Ratio, CI = Confidence Interval

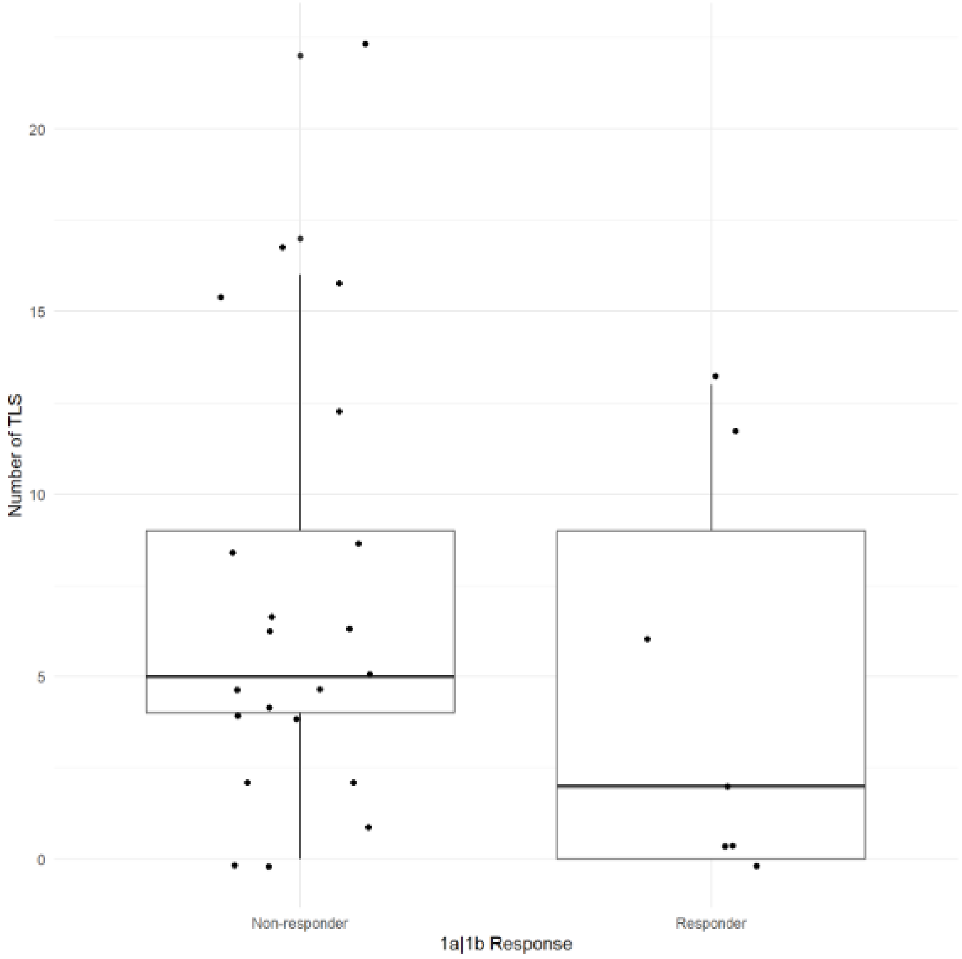

Supplement: Supplementary Figure S2 — Supplementary Figure 2: Tertiary lymphoid structures in surgical tumor samples, analysed separately depending on tumor response. [file ccr-25-0369_supplementary_figure_s2_suppfs2.pdf]
