# Supplementary material for: A Phase II Study of Perioperative Avelumab plus Chemotherapy for Patients with Resectable Gastric Cancer or Gastroesophageal Junction Cancer – The MONEO Study
Source: Clin Cancer Res. 2025 May 19;31(14):2890–8. doi: 10.1158/1078-0432.CCR-25-0369 (PMC12260514; doi:10.1158/1078-0432.CCR-25-0369)
Supplement: Supplementary Table S1 — Supplementary Table 1: Representativeness of study participants [file ccr-25-0369_supplementary_table_s1_suppts1.docx]

**Supplementary Table 1:** Representativeness of study participants

| Cancer type(s)/subtype(s)/stage(s)/condition | Gastric and gastroesophageal junction cancer  Locally advanced and resectable |
| --- | --- |
| **Considerations related to:** | |
| Sex | Incidence of gastric and gastroesophageal junction cancer in men is twice as high as in women. |
| Age | The average age of diagnosis is 68 years-old, although recent studies report an increase in non-cardia gastric cancer among young individuals, especially in low-incidence countries like Spain. |
| Race/Ethnicity | Gastric cancer disproportionately affects non-white racial and ethnic minority groups. |
| Geography | Gastric cancer displays substantial global variation in incidence; the highest rates are observed in Eastern Asia, Central and Eastern Europe and South America. |
| Other considerations | Gastric cancer (GC) represents the fifth more frequent tumor and the fourth cause of cancer-death, worldwide. |
| **Overall Representativeness of this study** | |
| The median age in this study was 64 years (range: 56-70 years). Most patients were male (55%). Most patients were not-hispanic, although 5 of them were hispanic. All patients were included in Western Europe, Spain. | |

Lordick F, Carneiro F, Cascinu S, Fleitas T, Haustermans K, Piessen G, Vogel A, Smyth EC; ESMO Guidelines Committee. Electronic address: clinicalguidelines@esmo.org. Gastric cancer: ESMO Clinical Practice Guideline for diagnosis, treatment and follow-up. Ann Oncol. 2022 Oct;33(10):1005-1020. doi: 10.1016/j.annonc.2022.07.004. Epub 2022 Jul 29. PMID: 35914639.

Bray F, Laversanne M, Sung H, Ferlay J, Siegel RL, Soerjomataram I, Jemal A. Global cancer statistics 2022: GLOBOCAN estimates of incidence and mortality worldwide for 36 cancers in 185 countries. CA Cancer J Clin. 2024 May-Jun;74(3):229-263. doi: 10.3322/caac.21834. Epub 2024 Apr 4. PMID: 38572751.

Shah SC, McKinley M, Gupta S, Peek RM Jr, Martinez ME, Gomez SL. Population-Based Analysis of Differences in Gastric Cancer Incidence Among Races and Ethnicities in Individuals Age 50 Years and Older. Gastroenterology. 2020 Nov;159(5):1705-1714.e2. doi: 10.1053/j.gastro.2020.07.049. Epub 2020 Aug 6. Erratum in: Gastroenterology. 2021 Apr;160(5):1904. doi: 10.1053/j.gastro.2021.03.035. PMID: 32771406; PMCID: PMC7680373.

Dong E, Duan L, Wu BU. Racial and Ethnic Minorities at Increased Risk for Gastric Cancer in a Regional US Population Study. Clin Gastroenterol Hepatol. 2017 Apr;15(4):511-517. doi: 10.1016/j.cgh.2016.11.033. Epub 2016 Dec 9. PMID: 27939654.
