# Supplementary material for: A Phase II Study of Perioperative Avelumab plus Chemotherapy for Patients with Resectable Gastric Cancer or Gastroesophageal Junction Cancer – The MONEO Study
Source: Clin Cancer Res. 2025 May 19;31(14):2890–8. doi: 10.1158/1078-0432.CCR-25-0369 (PMC12260514; doi:10.1158/1078-0432.CCR-25-0369)
Supplement: Supplementary Table S2 — Supplementary Table 2: Rates of pathological complete response (pCR) and non-pCR according to PD-L1 CPS cutoffs levels of 5 and 10. [file ccr-25-0369_supplementary_table_s2_suppts2.docx]

**Supplementary Table 2:** Rates of pathological complete response (pCR) and non-pCR according to PD-L1 CPS cutoffs levels of 5 and 10.

|  |  | pCR | non-pCR |
| --- | --- | --- | --- |
| CPS cut-off 5 | PD-L1 CPS < 5 | 13.3% (2/15) | 86.7% (13/15) |
|  | PD-L1 CPS ≥ 5 | 25.0% (4/16) | 75.0% (12/16) |
| CPS cut-off 10 | PD-L1 CPS < 10 | 15.8% (3/19) | 84.2% (16/19) |
|  | PD-L1 CPS ≥ 10 | 25.0% (3/12) | 75.0% (9/12) |

Legend: pCR: pathological complete response; PD-L1: programmed Death-Ligand 1; CPS: combined positive score
