# Supplementary material for: A Phase II Study of Perioperative Avelumab plus Chemotherapy for Patients with Resectable Gastric Cancer or Gastroesophageal Junction Cancer – The MONEO Study
Source: Clin Cancer Res. 2025 May 19;31(14):2890–8. doi: 10.1158/1078-0432.CCR-25-0369 (PMC12260514; doi:10.1158/1078-0432.CCR-25-0369)
Supplement: Supplementary Table S3 — Supplementary Table 3: Surrogacy between pCR and MPR status with overall survival [file ccr-25-0369_supplementary_table_s3_suppts3.docx]

**Supplementary Table 3:** Surrogacy between pCR and MPR status with overall survival

| Univariable Cox models | OS hazard ratio (95%CI) |
| --- | --- |
| - pCR (yes vs no) | 0.52 (0.06 – 4.33) |
| - MPR (yes vs no) | 0.33 (0.04 – 2.73) |

Legend: pCR: pathological complete response; MPR: major pathological response, OS: overall survival; CI: confidence interval
