# Supplementary material for: A Phase II Study of Perioperative Avelumab plus Chemotherapy for Patients with Resectable Gastric Cancer or Gastroesophageal Junction Cancer – The MONEO Study
Source: Clin Cancer Res. 2025 May 19;31(14):2890–8. doi: 10.1158/1078-0432.CCR-25-0369 (PMC12260514; doi:10.1158/1078-0432.CCR-25-0369)
Supplement: Supplementary Table S4 — Supplementary Table 4: Incidence of adverse events during the study treatment. [file ccr-25-0369_supplementary_table_s4_suppts4.docx]

**Supplementary Table 4:** Incidence of adverse events during the study treatment.

| **Type** | **All AEs** | | **AEs related to Avelumab** | |
| --- | --- | --- | --- | --- |
|  | **Any grade** | **Grade ≥3** | **Any grade** | **Grade ≥3** |
| **Any** | **40 (100%)** | **32 (80%)** | **29 (72.5%)** | **10 (25%)** |
| Fatigue | 33 (82.5%) | 10 (25%) | 18 (45%) | 3 (7.5%) |
| Neutropenia | 29 (72.5%) | 21 (52.5%) | 10 (25%) | 7 (17.5%) |
| Diarrhoea | 29 (72.5%) | 3 (7.5%) | 14 (35%) | 0 (0%) |
| Nausea/Vomiting | 25 (62.5%) | 5 (12.5%) | 10 (25%) | 0 (0%) |
| Neurotoxicity | 20 (50%) | 0 (0%) | 6 (15%) | 0 (0%) |
| Anorexia | 17 (42.5%) | 1 (2.5%) | 8 (20%) | 1 (2.5%) |
| Anaemia | 17 (42.5%) | 0 (0%) | 6 (15%) | 0 (0%) |
| Dysgeusia | 14 (35%) | 0 (0%) | 3 (7.5%) | 0 (0%) |
| Constipation | 10 (25%) | 0 (0%) | 0 (0%) | 0 (0%) |
| Mucositis oral | 10 (25%) | 0 (0%) | 1 (2.5%) | 0 (0%) |
| Abdominal pain | 10 (25%) | 0 (0%) | 2 (5%) | 0 (0%) |
| Alopecia | 9 (22.5%) | 0 (0%) | 2 (5%) | 0 (0%) |
| Dyspepsia | 8 (20%) | 0 (0%) | 2 (5%) | 0 (0%) |
| Arthralgia | 7 (17.5%) | 1 (2.5%) | 5 (12.5%) | 1 (2.5%) |
| Dysesthesia | 7 (17.5%) | 0 (0%) | 1 (2.5%) | 0 (0%) |
| Aspartate aminotransferase increased | 6 (15%) | 0 (0%) | 3 (7.5%) | 0 (0%) |
| Leukocytes count decreased | 5 (12.5%) | 3 (7.5%) | 2 (5%) | 1 (2.5%) |
| Hypertension | 5 (12.5%) | 2 (5%) | 1 (2.5%) | 0 (0%) |
| Insomnia | 5 (12.5%) | 0 (0%) | 0 (0%) | 0 (0%) |
| Dysphagia | 5 (12.5%) | 0 (0%) | 0 (0%) | 0 (0%) |
| Epigastralgia | 5 (12.5%) | 0 (0%) | 1 (2.5%) | 0 (0%) |
| Fever | 5 (12.5%) | 0 (0%) | 1 (2.5%) | 0 (0%) |
| Platelet count decreased | 4 (10%) | 1 (2.5%) | 0 (0%) | 0 (0%) |
| Alanine aminotransferase increased | 4 (10%) | 0 (0%) | 3 (7.5%) | 0 (0%) |
| Dizziness | 4 (10%) | 0 (0%) | 0 (0%) | 0 (0%) |
| Rash | 4 (10%) | 0 (0%) | 1 (2.5%) | 0 (0%) |
| Musculoeskeletal pain | 4 (10%) | 0 (0%) | 0 (0%) | 0 (0%) |
| Hypokalemia | 3 (7.5%) | 1 (2.5%) | 1 (2.5%) | 0 (0%) |
