# Supplementary material for: A Phase II Study of Perioperative Avelumab plus Chemotherapy for Patients with Resectable Gastric Cancer or Gastroesophageal Junction Cancer – The MONEO Study
Source: Clin Cancer Res. 2025 May 19;31(14):2890–8. doi: 10.1158/1078-0432.CCR-25-0369 (PMC12260514; doi:10.1158/1078-0432.CCR-25-0369)
Supplement: Supplementary Methodology S1 — Supplementary Methodology [file ccr-25-0369_supplementary_methodology_s1_suppms1.docx]

## Supplementary Methodology

### Detailed procedures for biomarker analyses:

**Immunohistochemistry staining and analysis**

Programmed death-ligand 1 (PD-L1) expression was centrally determined by immunohistochemistry (IHC) using DAKO PD-L1 (22C3) assay (RRID: AB_2833074). Combined positive score (CPS) is defined as the number of PD-L1–staining cells (tumor cells, lymphocytes and macrophages) divided by the total number of viable tumor cells and multiplied by 100. The upper bound of CPS is 100. Deficiency of mismatch repair (dMMR) proteins was also centrally evaluated by IHC. Tumors were considered dMMR if at least one of the following criteria was present: tumor showed loss of ≥1 MMR proteins MLH1 (IgG1, mouse monoclonal, clone: ES05, ready-to-use; Agilent, Cat# IR07961l, RRID: AB_3675711), MSH2 (IgG1, mouse monoclonal, clone: FE11, ready-to-use; Agilent, Cat# M363929-2, RRID: AB_2631353), MSH6 (IgG, rabbit monoclonal, clone: EP49, ready-to-use; Agilent, Cat# IR08661-2, RRID: AB_3675713) or PMS2 (mouse monoclonal, clone: A16-4, ready-to-use; Roche Diagnostics, Cat# 790-5094, RRID: AB_3669003). HER2 status was determined by IHC using VENTANA anti-HER2/neu (4B5) rabbit monoclonal primary antibody (VENTANA pathway HER2, clone: 4B5, Roche Diagnostics, Cat# 790-4493 RRID: AB_2921204), according to the FDA guidelines. HER2 scores of 2+ were further analyzed through silver in situ hybridization (SISH) using the Ventana INFORM HER2 Dual ISH DNA Probe Cocktail (Roche Diagnostics, Cat# 800-6043, RRID: AB_3675716).

**Multiplex immunofluorescence staining and analysis**

Multiplex immunofluorescence staining and analysis was performed as previously described on a Bond RX autostainer (Abengozar-Muela, Esparza et al. 2020, López-Janeiro, Villalba-Esparza et al. 2022). Four-microns-thick FFPE tissue sections were deparaffinized (Bond DeWax, Leica Biosystems) and rehydrated per standard protocols. Antigen retrieval was performed with Bond Epitope Retrieval Solution 1 (ER1, Leica Biosystems, Cat# AR9961) or 2 (ER2, Leica Biosystems, Cat# AR9640), followed by four sequential cycles of staining with each cycle including a 30-minute combined block and primary antibody incubation (Akoya antibody diluent/block, Akoya Biosciences, Cat# ARD1001EA), followed by a secondary HRP-conjugated polymer. Signal amplification was achieved with TSA-Opal fluorophores. Between staining cycles, tissue sections underwent heat-induced epitope retrieval to remove the primary/secondary-HRP antibody complexes before staining with the next antibody. The primary antibodies and corresponding fluorophores are anti-CD3 (rabbit polyclonal, IgG, ready-to-use, Agilent, Cat# IR503, RRID: AB_3094578) in Opal 480 (Akoya Biosciences, Cat# FP1500001KT); anti-CD8 (mouse monoclonal, clone C8/144B, ready-to-use, Agilent, product number GA62361-2, RRID:AB_3073940) in Opal 520 (Akoya Biosciences, Cat# FP1487001KT); anti- CD20 (IgG2α, mouse monoclonal, clone: L26, ready-to-use; Agilent, Cat# M075501-2, RRID: AB_2282030) in Opal 690 (Akoya Biosciences, Cat# FP1497001KT); anti-CD68 (mouse monoclonal, clone: PG-M1, ready-to-use; Agilent, Cat# M087601-2, RRID: AB_2892734) in Opal 620 (Akoya Biosciences, Cat# FP1495001KT); anti- CD66b (IgG1, mouse monoclonal, clone: ANC1D5, dilution: 1:200; LS-Bio, Cat# LS‑C357668, RRID: AB_3675718) in Opal 570 (Akoya Biosciences, Cat# FP1488001KT); and anti-cytokeratin (mouse monoclonal, clone AE1/AE3, ready-to-use, Leica Biosystems, Cat# NCL-L-AE1/AE, Cat# AE1/AE3-601-L-CE, RRID: AB_2924990) in Opal 780. We counterstained nuclei with Spectral DAPI (Akoya Biosciences, Cat# FP1490) and mounted the stained tissues with ProLong Diamond Antifade mounting medium (Thermo Fisher Scientific, Cat# P36965). The stained slides were scanned using the PhenoImager™ HT Automated Quantitative Pathology Imaging System (Akoya Biosciences). After image acquisition, unmixing of the spectral libraries was performed with inForm software (Akoya Biosciences, RRID: SCR_019155). Unmixed images were then imported into the open-source digital pathology software QuPath version 0.4.4 for analysis (RRID: SCR_018257). Whole tumor regions from each slide were analyzed for each sample. Marker expression was used to identify tumor cells (CK), T cell population expressing (CD3+), cytotoxic T cells (CD3+CD8+), B cells (CD20+), macrophages (CD68+), and neutrophils (CD66b+). CD4+ T cells were defined as CD3+ CD8-. Densities of each cell population were quantified and expressed as number of cells per mm2. Lymphoid aggregates were identified as tertiary lymphoid structures (TLS) if possessing organized T cell and B cell regions.

Immunohistochemistry analyses for CD8PD1 and CD8KI67: Double stain was performed on a Discovery Ultra autostainer. In brief, paraffin sections were cut at 4 μm, heat-induced antigen retrieval was carried out using Cell Conditioning 1 (CC1, Ventana Medical Systems, Cat# 06414575001) for 48 min at 95 °C (Ki-67/CD8 double) or 64 min at 95 °C (PD-1/CD8 double). Ki-67 was detected using clone 30-9 (rabbit monoclonal, ready-to-use; Ventana Medical Systems, Cat# 05278384001, RRID: AB_2631262). PD-1 was detected using clone NAT105 (mouse monoclonal, ready-to-use; Ventana Medical Systems, Cat# 07099029001, RRID: AB_3674657). The Ki67- and PD-1-bound anti-bodies were visualized using Discovery anti- NP AP (ready-to-use, Ventana Medical Systems, Cat# 07425325001) followed by the Discovery Yellow detection kit (Ventana Medical Systems, Cat# 07698445001), according to the manufacturer's recommendations. In the second sequence of the double-staining procedure, CD8 was detected using clone C8/144B (mouse monoclonal, ready-to-use, Agilent, product number GA62361-2, RRID:AB_3073940). CD8 was visualized using anti-mouse HQ (ready-to-use, Ventana Medical systems, Cat# 07017782001) followed by anti-HQ HRP (ready-to-use, Ventana Medical systems, Cat# K22062), followed by the Discovery Purple Detection kit (Ventana Medical Systems). Slides were counterstained with Hematoxylin and Bluing Reagent (Ventana Medical Systems). A Aperio CS2scanner from Leica Biosystems was used to scan the slides at a ×40 magnification. Densities of CD8+Ki67+ and CD8+PD-1+ were quantified and expressed as number of cells per mm2.

**Peripheral blood analyses**

Blood samples were obtained at the indicated time points. Part of the samples were preserved with TransFix™ (Invitrogen™ Cellular Antigen Stabilizing Reagent) to preserve myeloid cells until the analysis. Peripheral blood mononuclear cells were isolated by density gradient centrifugation with Ficoll-Paque™ (Cytiva). These PBMCs were used to study myeloid derived suppressor cell populations. The rest of the populations were studied directly in fresh blood samples. Erythrocytes were lysed with BD Pharm Lyse™ Lysing Buffer (BD biosciences).

For flow cytometry staining, cells were pretreated with 10μg/mL of human IgG to reduce nonspecific staining (Beriglobina P^©^, CSL Behring). Monoclonal antibodies to the human antigens were conjugated to fluorescein isothiocyanate (FITC), phycoerythrin (PE), PerCP-Cy5.5, allophycocyanin (APC), AlexaFluor 488, Alexa Fluor 647, APC-Cy7, Brillant Violet 510, Pacific Blue or Brillant Violet 421.

Anti-CD3 (UCHT1, RRDI: AB_2565849), anti-CD4 (OKT4, RRDI: AB_1186122), anti-CD8 (HIT8a, RRDI: AB_314112), anti-CD11b (ICRF44, RRDI: AB_314159), anti-CD14 (M5E2, RRDI: AB_2922652), anti-CD15 (H198, RRDI: AB_314200), anti-CD16 (368, RRDI: AB_2562085), anti-CD19 (6D5, RRDI: AB_3133642), anti-CD25 (BC96, RRDI: AB_10896914), anti-CD33 (HIM3-4, RRDI: AB_314344), anti-CD39 (2A1, RRDI: AB_2563265), anti-CD45RA (HI100, RRDI: AB_893357), anti-CD137 (4B4-1, RRDI: AB_2563830), anti-CX3CR1 (29A-1, RRDI: AB_2892303), anti-PD-1 (EH12-2H7, RRDI: AB_2910394), anti-CTLA4 (L3D10, RRDI: AB_10680785), anti-HLA-DR (L243, RRDI: AB_493586) and anti-TIM3 (F38-2F2, RRDI: AB_2565716) were purchased from Biolegend. Anti-CD56 (NCAM16.2, RRDI_AB_2732054), anti-Eomes (WD1928, RRDI: AB_2916484), anti-CD45R0 (UCHL1, RRDI: AB_10894205), anti-CCR7 (3D12, RRDI: AB_394352) and anti-Ki-67 (B56, RRDI: AB_647087) were obtained from BD Bioscience. The anti-Tbet (eBio4B10, RRDI: AB_11042699) and Foxp3 (FJK-16s, RRDI: AB_465935) were from Thermo Fisher Scientific and the anti-KLRG1 (2F1, RRDI: AB_637864) was from Santa Cruz. Intracellular staining (to study T-bet, Eomes, Foxp3 and Ki-67 expression) was performed with the True-Nuclear™ Transcription Factor Buffer Set (Biolegend) following the manufacturer’s protocol. Dead cells were identified using the ZombieNIR™kit (Biolegend). FACSCanto II cytometer was used for cell acquisition, and data analysis was performed using FACS DiVa (BD Biosciences) and FlowJo 7.2.1 (Tree Star Inc., San Carlos, CA).

CD8 T cells were defined as CD3^+^CD8^+^CD45^+^ cells. CD4 T cells were defined as CD3^+^CD4^+^CD45^+^ cells. PMN-MDSC were gated in CD11b^+^CD14^-^CD15^+^, M-MDSCs in CD11b^+^CD14^+^HLA-DR^-/lo^CD15^-^ and e-MDSC in Li^-^CD14^-^CD15^-^HLA-DR^-^CD33^+^.

Sera were obtained at the indicated time points by blood centrifugation and then were aliquoted and preserved at -80ºC until their analysis. Cytokines and chemokines were determined with a ProcartaPlex™ kit for Luminex technology (Cytokine & Chemokine Convenience 34-Plex Human ProcartaPlex™ Panel 1A. Invitrogen) following the manufacter instructions and using a MAGPIX™.

**Transcriptomic analyses of the surgery specimen**

VHIOε00 (Epsilon) is a targeted NGS custom panel based on a capture enrichment approach designed for the detection of gene fusion events, the analysis of individual gene expression level and the characterization of a gene expression signature scoring the inflammatory status of the tumor microenvironment (VIGEX) (Hernando-Calvo, Vila-Casadesús et al. 2023). RNA from tumor FFPE samples is obtained (Maxwell(R) CSC RNA FFPE Kit (Promega) (Maxwell RSC Instrument (RRID:SCR_025867)) and cDNA is synthesized, ligated to Illumina-adapters and enriched for targeted regions using biotinylated probes (Illumina Custom Enrichment Panel). After enrichment, libraries are sequenced using the Illumina Sequencing By Synthesis technology. Sequencing reads are aligned (STAR-2.7.10a (STAR (RRID:SCR_004463))) against the hg38 reference. Gene counts are quantified with htseq v2.0.2 (HTSeq (RRID:SCR_005514) and normalised with DESeq2 (DESeq2 (RRID:SCR_015687).

Abengozar-Muela, M., M. V. Esparza, D. Garcia-Ros, C. E. Vásquez, J. I. Echeveste, M. A. Idoate, M. D. Lozano, I. Melero and C. E. de Andrea (2020). "Diverse immune environments in human lung tuberculosis granulomas assessed by quantitative multiplexed immunofluorescence." Mod Pathol **33**(12): 2507-2519.

Hernando-Calvo, A., M. Vila-Casadesús, Y. Bareche, A. Gonzalez-Medina, F. Abbas-Aghababazadeh, D. Lo Giacco, A. Martin, O. Saavedra, I. Brana, M. Vieito, R. Fasani, J. Stagg, F. Mancuso, B. Haibe-Kains, M. Han, R. Berche, T. J. Pugh, O. Mirallas, J. Jimenez, N. S. Gonzalez, C. Valverde, E. Muñoz-Couselo, C. Suarez, M. Diez, E. Élez, J. Capdevila, A. Oaknin, C. Saura, T. Macarulla, J. C. Galceran, E. Felip, R. Dienstmann, P. L. Bedard, P. Nuciforo, J. Seoane, J. Tabernero, E. Garralda and A. Vivancos (2023). "A pan-cancer clinical platform to predict immunotherapy outcomes and prioritize immuno-oncology combinations in early-phase trials." Med **4**(10): 710-727.e715.

López-Janeiro, Á., M. Villalba-Esparza, M. E. Brizzi, D. Jiménez-Sánchez, I. Ruz-Caracuel, E. Kadioglu, I. Masetto, V. Goubert, D. Garcia-Ros, I. Melero, A. Peláez-García, D. Hardisson and C. E. de Andrea (2022). "The association between the tumor immune microenvironments and clinical outcome in low-grade, early-stage endometrial cancer patients." J Pathol **258**(4): 426-436.
