# Supplementary material for: A Phase II Study of Perioperative Avelumab plus Chemotherapy for Patients with Resectable Gastric Cancer or Gastroesophageal Junction Cancer – The MONEO Study
Source: Clin Cancer Res. 2025 May 19;31(14):2890–8. doi: 10.1158/1078-0432.CCR-25-0369 (PMC12260514; doi:10.1158/1078-0432.CCR-25-0369)
Supplement: Supplementary Data S1 — Study protocol [file ccr-25-0369_supplementary_data_s1_suppds1.pdf]

Phase II Study of Avelumab plus chemotherapy in the peri-operative treatment for patients with resectable Gastric cancer (GC) or Gastroesophageal Junction cancer (GEJC)

Clinical Study Protocol

VHIO code	VHIO19001
Short Title	MONEO Study
Phase	II
Investigational Product	Avelumab
EudraCT Number	2019-000782-21
Principal Investigator	Ignacio Melero, Maria Alsina
Sponsor	VHIO Vall d'Hebron Institute of Oncology<br>C/ Natzaret, 115-117<br>08035 Barcelona
Protocol Version and Date	Version 9, 21 May 2020

CONFIDENTIAL

The information contained in this document is confidential and the property of the VHIO. The information may not - in full or in part - be transmitted, reproduced, published, or disclosed to others than the applicable Competent Ethics Committee(s) and Regulatory Authority(ies) without prior written authorisation from the sponsor except to the extent necessary to obtain informed consent from those who will participate in the study.

Signature Page(s)

Study Number	
Study Title	Phase II Study of Avelumab plus chemotherapy in the peri-operative treatment for patients with resectable Gastric cancer (GC) or Gastroesophageal Junction cancer (GEJC)

The Sponsor-Investigator have approved the protocol version 9, dated 21 May 2020, and confirm hereby to conduct the study according to the protocol, current version of the World Medical Association Declaration of Helsinki, ICH-GCP guidelines or ISO 14155 norm if applicable and the local legally applicable requirements.

Sponsor representative:

Place/Date	Signature

Principal Investigator 1:

Place/Date	Signature

Principal Investigator 2:

Place/Date	Signature

Local Principal Investigator at study site*:

I have read and understood this trial protocol and agree to conduct the trial as set out in this study protocol, the current version of the World Medical Association Declaration of Helsinki, ICH-GCP guidelines or ISO 14155 norm and the local legally applicable requirements.

Site	Name and address of site
Principal investigator	Printed name of Principal investigator

Place/Date	Signature

**Note:* In multicentre studies, this page must be individually signed by all participating Local Principal Investigators.

TABLE OF CONTENTS

ABBREVIATIONS 8
STUDY SYNOPSIS 10
STUDY SCHEDULE 15

1. STUDY ADMINISTRATIVE STRUCTURE 19
1.1 SPONSOR 19
1.2 PRINCIPAL INVESTIGATOR(S) 19
1.3 INVESTIGATIONAL SITES 19
1.4 LABORATORY 19
1.5 MONITORING INSTITUTION 19
2. BACKGROUND INFORMATION 20
2.1 GASTRIC CANCER 20
2.2 IMMUNOTHERAPY AND THE PD-1/PD-L1 CHECKPOINT PATHWAY 20
2.3 AVELUMAB 21
2.4 RATIONALE FOR COMBINATION OF FLOT PLUS AVELUMAB 23
2.5 RATIONALE FOR MAINTENANCE THERAPY WITH AVELUMAB 24
2.6 BENEFIT/RISK ASSESSMENT 24
2.6.1 POTENTIAL BENEFITS 24
2.6.2 RISKS ASSOCIATED WITH AVELUMAB 24
2.6.3 OVERALL BENEFIT-RISK 25
2.7 KEY POINT: BIOMARKER ANALYSIS 25
3. STUDY OBJECTIVES 27
3.1 PRIMARY OBJECTIVE 27
3.2 SECONDARY OBJECTIVES 27
4. STUDY DESIGN 28
4.1 OVERALL STUDY DESIGN 28
4.2 STUDY SCHEMA 29
5. STUDY POPULATION 30
5.1 INCLUSION CRITERIA 30
5.2 EXCLUSION CRITERIA 31
5.3 CRITERIA FOR WITHDRAWAL / DISCONTINUATION OF PARTICIPANTS 32
6. TREATMENT OF SUBJECTS 34
6.1 TREATMENT PLAN SCHEDULE 34
6.2 DOSAGE AND ADMINISTRATION 34
6.2.1 AVELUMAB DOSAGE, ADMINISTRATION AND PRE-MEDICATION 34
6.2.1.1 SPECIAL PRECAUTIONS 35
6.2.2 FLOT CHEMOTHERAPY DOSAGE AND ADMINISTRATION 35
6.2.2.1 PRE-TREATMENT CONSIDERATIONS 36
6.2.2.2 ADMINISTRATION OF FLOT CHEMOTHERAPY REGIMEN 36
6.2.2.3 ANTI-EMETIC RECOMMENDATIONS 36
6.3 TOXICITY MANAGEMENT GUIDELINES 37
6.3.1 TOXICITY AND GRADING CRITERIA 37
6.3.2 FLOT CHEMOTHERAPY TOXICITY MANAGEMENT 37

6.3.2.2 DOSE DELAY AND MODIFICATION. GENERAL REMARKS 38
6.3.3 AVELUMAB TOXICITY MANAGEMENT 39
6.3.3.2 DOSE MODIFICATIONS FOR AVELUMAB TOXICITIES 39

6.4 DISTRIBUTION, PACKAGING AND LABELING OF THE INVESTIGATIONAL MEDICINAL PRODUCT 39

6.5 PREPARATION, HANDLING, AND STORAGE OF THE INVESTIGATIONAL MEDICINAL PRODUCT 40

6.6 INVESTIGATIONAL MEDICINAL PRODUCT ACCOUNTABILITY 41

6.7 CONCOMITANT MEDICATIONS AND THERAPIES 42

6.7.1 PERMITTED MEDICINES 42
6.7.2 PROHIBITED MEDICINES 43

6.8 SURGICAL CONSIDERATIONS 43

6.8.1 GASTRIC RESECTION 43
6.8.2 EXTENT OF LYMPHADENECTOMY 44
6.8.3 SURGICAL QUALITY CONTROL 47
6.8.4 OPERATIVE TECHNIQUE 47
6.8.5 POSTOPERATIVE PROCEDURES 48
6.8.5.2 PATHOLOGICAL ASPECTS 49

6.9 SUBJECT FOLLOW-UP 49

6.9.1 EXTENDED SAFETY FOLLOW-UP 49
7. STUDY PROCEDURES AND ASSESSMENTS 50

7.1 SCHEDULE OF ASSESSMENTS 50

7.1.1 PRE-TREATMENT EVALUATION: 50
7.1.2 ASSESSMENTS PRIOR TO STARTING C1 OF FLOT CHEMOTHERAPY PLUS AVELUMAB (PRE-OPERATIVE AND POST-OPERATIVE; DAY 1, OR UP TO 3 CALENDAR DAYS BEFORE THE CYCLE). 51
7.1.3 ASSESSMENTS PRIOR TO STARTING C2, C3 AND C4 OF FLOT CHEMOTHERAPY PLUS AVELUMAB (PRE- OPERATIVE AND POST-OPERATIVE; DAY 1, OR UP TO 3 CALENDAR DAYS BEFORE THE CYCLE). 51
7.1.4 ASSESSMENTS PRIOR TO SURGERY: 51
7.1.5 ASSESSMENTS DURING AVELUMAB (POST-OPERATIVE, POST FLOT CHEMOTHERAPY) 52
7.1.6 END OF TREATMENT 52
7.1.7 SAFETY FOLLOW-UP 53
7.1.8 NON-COMPLIANCE WITH PRE-OP FLOT AND/OR POST-OP FLOT 53
7.1.9 SCHEDULE OF ASSESSMENTS 53

7.2 DEMOGRAPHIC AND OTHER BASELINE CHARACTERISTICS 53

7.2.1 DEMOGRAPHIC DATA 53
7.2.2 DIAGNOSIS OF GASTRIC CANCER 54
7.2.3 MEDICAL HISTORY 54
7.2.4 VITAL SIGNS AND PHYSICAL EXAMINATION 54
7.2.5 CLINICAL LABORATORY ASSESSMENTS 54
7.2.6 CT OR MRI SCANS FOR TUMOR ASSESSMENT AT BASELINE 56

7.3 EFFICACY ASSESSMENTS 56

7.3.1 PATHOLOGICAL COMPLETE RESPONSE RATE 56
7.3.2 ASSESSMENT OF SECONDARY OUTCOMES 57
7.3.3 ASSESSMENT OF EXPLORATORY OUTCOMES 58

7.4 ASSESSMENT OF SAFETY 59

7.4.1 DEFINITIONS ADVERSE EVENT 59
SERIOUS ADVERSE EVENT 60
ADVERSE DRUG REACTION 60
SUSPECTED UNEXPECTED SERIOUS ADVERSE REACTION (SUSAR) 61
7.4.2 EVENTS NOT TO BE TREATED AS SAES 61
7.4.3 ADVERSE EVENTS OF SPECIAL INTEREST AND PREGNANCY 61
7.4.4 COLLECTION AND RECORDING OF ADVERSE EVENTS, INCL. SAES AND ADVERSE EVENTS OF SPECIAL INTEREST AND PREGNANCY 62

7.4.5 DEFINITION OF THE ADVERSE EVENT REPORTING PERIOD 63
7.4.6 PROCEDURE FOR REPORTING OF SERIOUS ADVERSE EVENTS, ADVERSE EVENTS OF SPECIAL INTEREST AND PREGNANCY 64
7.4.7 SAFETY REPORTING TO HEALTH AUTHORITIES, INDEPENDENT ETHICS COMMITTEES / INSTITUTIONAL REVIEW BOARDS AND INVESTIGATORS 64
• SERIOUS ADVERSE EVENTS 64
8. STATISTICAL METHODS 66

8.1 HYPOTHESIS 66

8.2 DETERMINATION OF SAMPLE SIZE 66

8.3 FEASIBILITY OF ENROLLING PROPOSED POPULATION 66

8.4 STATISTICAL CRITERIA OF TERMINATION OF TRIAL 67

8.5 PLANNED ANALYSES 67

8.5.1 DEFINITION OF STUDY POPULATIONS FOR ANALYSIS 67
8.5.2 GENERAL CONSIDERATIONS 67
8.5.3 ANALYSIS OF PRIMARY ENDPOINTS 67
8.5.4 ANALYSIS OF SECONDARY ENDPOINTS 68
8.5.5 ANALYSIS OF EXPLORATORY ENDPOINTS 69
8.5.6 ANALYSIS OF SAFETY ENDPOINTS 70

9. ADMINISTRATIVE ASPECTS 71

9.1 ETHICAL CONDUCT OF THE STUDY 71

9.1.1 INDEPENDENT ETHICS COMMITTEE 71
9.1.2 PATIENT INFORMATION AND INFORMED CONSENT 71

9.2 CONFIDENTIALITY 72

9.3 PROTOCOL AMENDMENTS 72

9.4 STORAGE OF SAMPLES 73

9.5 DATA HANDLING AND RECORD KEEPING 73

9.6 STUDY MONITORING 73

9.6.1 RESPONSIBILITIES OF THE INVESTIGATORS 73

9.7 AUDIT AND INSPECTION 74

9.8 CLINICAL STUDY REPORT 74

9.9 PUBLICATION POLICY 74

10. REFERENCES 75
11. APPENDICES 80

11.1 RECOMMENDATION FOR DOSE MODIFICATIONS AND TOXICITY MANAGEMENT FOR FLOT CHEMOTHERAPY 80

11.1.2 OXALIPLATIN NEUROTOXICITY 80
NEUROTOXICITY ≤ 7 DAYS >7 AND < 14 DAYS PRESENT BETWEEN CYCLES 80
11.1.3 OXALIPLATIN RENAL TOXICITY 81
CREATININE CLEARANCE OXALIPLATIN DOSE 81
11.1.4 DOCETAXEL LIVER TOXICITY 81
11.1.5 OTHER TOXICITIES FOR FLOT 82

11.2 DOSE MODIFICATIONS AND TOXICITY MANAGEMENT FOR AVELUMAB 82

11.2.1 INFUSION-RELATED REACTIONS 82
11.2.2 SEVERE HYPERSENSITIVITY REACTIONS AND FLUE-LIKE SYMPTOMS 83
11.2.3 TUMOR LYSIS SYNDROME 84
11.2.4 IMMUNE-RELATED ADVERSE EVENTS 85

Tables

Table 1. Study schedule 15
Table 2. FLOT Therapy Regimen Dose definition and number of cycles 35
Table 3. Doses of 5-HT3 Antagonists 37
Table 4. Hematology Laboratory Tests 55
Table 5. Serum chemistry Laboratory Tests....................55
Table 6. Urinalysis Tests * 55
Table 7. Coagulation Tests 55
Table 8. Pathological response criteria according to Becker scoring 57
Table 9. Oxaliplatin dose modification in case of neurotoxicity....................80
Table 10. Oxaliplatin dose modification in case of nephrotoxicity....................81
Table 11. Docetaxel dose modification in case of related liver toxicity....................81
Table 12. Treatment Modification for Symptoms of Infusion-related Reactions Associated with avelumab 82
Table 13. Management of Immune-related Adverse Events....................86

Figures

Figure 1. Study Design....................29
Figure 2. Treatment schedule 34
Figure 3. Gastric Lymph Node Stations....................46
Figure 4. Assessment and Initial Management of Tumor Lysis Syndrome 84

ABBREVIATIONS

5-FU	Fluorouracil
ADA	Anti-drug antibody
ADR	Adverse Drug Reaction
AE	Adverse Event
AESI	Adverse Event of Special Interest
ANCOVA	Analysis of Covariance
AST	Aspartate Aminotransferase
β-hCG	β-human chorionic gonadotropin
BOR	Best Overall Response
CI	Confidence Interval
CR	Complete Response
CT	Computed Tomography
CTCAE	Common Terminology Criteria for Adverse Events
ECG	Electrocardiogram
ECOG PS	Eastern Cooperative Oncology Group Performance Status
eCRF	Electronic Case Report Form
FFPE	Formalin-fixed Paraffin-embedded
FLOT	5-FU, Leucovorin, Oxaliplatin and Taxotere (Docetaxel)
CG	Gastric cancer
GCP	Good Clinical Practice
GEJ	Gastro-esophageal junction
GGT	Gamma-glutamyltransferase
HBV	Hepatitis B virus
HCV	Hepatitis C virus
IB	Investigator’s Brochure
ICF	Informed Consent Form
CH	International Council for Harmonization
IEC	Independent Ethics Committee
Ig	Immunoglobulin
Iv	Intravenous
IHC	Immunohistochemistry
IMP	Investigational Medicinal Product
irAE	Immune-related Adverse Event
IRC	Independent Review Committee
ITT	Intent-to-treat
IV	Intravenous(ly)

MedDRA	Medical Dictionary for Regulatory Activities
MRI	Magnetic Resonance Imaging
NCI	National Cancer Institute
ORR	Objective Response Rate
OS	Overall Survival
pCR	Pathological Complete Response
PD	Progressive Disease
PD-1	Programmed Death 1 (receptor)
PD-L (1 and 2)	Programmed Death Ligand (1 and 2)
PFS	Progression-free Survival
PP	Per-Protocol
PR	Partial Response
RECIST	Response Evaluation Criteria in Solid Tumors
RR	Response rate
SAE	Serious Adverse Event
SAP	Statistical Analysis Plan
SD	Stable Disease
SUSAR	Suspected Unexpected Serious Adverse Reactions
TSH	Thyroid-stimulating Hormone
WHO	World Health Organization

STUDY SYNOPSIS

**Sponsor**	VHIO Vall d´Hebron Institute of Oncology
**Study Title:**	Phase II Study of Avelumab plus chemotherapy in the peri-operative treatment for patients with resectable Gastric cancer (GC) or Gastroesphageal Junction cancer (GEJC)
**Short Title**	MONEO Study
**Protocol Version and Date:**	Version 9 of 21 May 2020
**Protocol number /EudraCT number**	VHIO19001/2019-000782-21
**Clinical Phase:**	Phase II
**Hypothesis:**	The addition of Avelumab to the perioperative chemotherapy in GC and GEJC patients may increase pathological responses by a synergic effect activating the immune response. Conclusively, the survival of these patients would improve.
**Objective(s):**	The **primary objective** is to investigate whether the addition of avelumab to FLOT chemotherapy (docetaxel, oxaliplatin and fluorouracil/leucovorin) improves efficacy in terms of pathological complete response (pCR) rate, in GC and GEJC patients compared to the historical data of chemotherapy alone in the neoadjuvant setting.<br><br>**Secondary objectives**<br><br>Secondary objectives are as follows:<br>• To evaluate the addition of avelumab to the perioperative chemotherapy in regard to the following:<br>o Overall survival (OS)<br>o Disease-free survival (DFS)<br>o Progression-free survival (PFS)<br>o Surgical resection rate (R0)<br>o Overall Response Rate (ORR) to neoadjuvancy

	• To determine the safety and tolerability of avelumab with FLOT chemotherapy.<br>• To perform a comprehensive analysis of biomarkers, as exploratory endpoints.
**Outcome(s):**	Primary efficacy endpoint:<br>• Pathological complete response (pCR) rate, where pCR is defined as the absence of residual tumor based on evaluation of the resected esophagogastric specimen according to Becker remission criteria [1].<br>Secondary efficacy endpoints:<br>• Overall survival (OS) [time frame: from the initial date of neoadjuvant chemotherapy to the date of death due to any cause. Patients without documentation of death at the time of analysis will be censored at the last follow-up date]. Estimated using Kaplan-Meier method.<br>• Disease-free survival (DFS) [time frame: from the surgery to the first observation of disease relapse or death due to any cause. Patients without an event prior to the time of analysis will be censored at the last relapse-free assessment]. Relapse is defined according to RECIST v1.1. Estimated using Kaplan- Meier method.<br>• Progression-free survival (PFS) [time frame: from the initial date of neoadjuvant chemotherapy to the date of first documentation of disease progression or death due to any cause, whichever occurs first. Patients without an event prior to the time of analysis will be censored at the last assessment that is stable disease (SD) or better]. Progression is defined according to RECIST v1.1. Estimated using Kaplan-Meier method.<br>• Surgical complete resection rate (R0). This is a complete macroscopic resection of the gross tumor with negative surgical margins.

	• Overall Response rate (ORR) to neoadjuvancy, as the proportion of subjects with complete response (CR)and partial response (PR), according to RECIST v1.1. [time frame: from the initial date of neoadjuvant chemotherapy to 3 years post- treatment).<br>Safety endpoints: safety of the combination of avelumab with FLOT chemotherapy (docetaxel, oxaliplatin and fluorouracil/leucovorin)<br>Exploratory Endpoints<br>• Pathological immune response (pIR)<br>• Characterization of the immune contexture<br>• Immunodynamic follow-up<br>• TCR clonality assessment
**Study design:**	This is an open-label, non-randomized, multicentric phase II clinical trial in subjects with operable gastric or GEJ adenocarcinoma. Tissue biopsies before and after treatment will be required. Blood samples will be required at different points of the treatment for biomarker analyses. Tumor imaging assessments will be performed at baseline, after the neoadjuvant treatment, and after finalizing the adjuvancy with avelumab/FLOT, and every 6 months thereafter to determine response to treatment. Clinical decision making will be based on Investigator assessment of the scans using RECIST v1.1. Safety of avelumab/FLOT will be monitored continuously by careful monitoring of all adverse events (AEs) and serious adverse events (SAEs) reported.
**Study population**	Patients with resectable gastric and GEJ cancer suitable for preoperative chemotherapy.
**Study Product / Intervention:**	Peri-operatory treatment consisting of four cycles (each cycle is 14 days) of neoadjuvant chemotherapy (docetaxel, oxaliplatin and fluorouracil/leucovorin) plus avelumab previous to surgery. Surgery should be scheduled 4 to 6 weeks after the last dose. Afterwards (4 to 10 weeks after surgery), four cycles of adjuvant therapy with the same schema, followed by avelumab up to 20 more cycles.<br>The dose, frequency and route of administration of study drugs is described below:

	Drug / Dose / Frequency of administration / Rout of administration:<br>Docetaxel / 50 mg/m2 / Day 1 Q2W / Intravenous<br>Oxaliplatin / 85mg/m2 / Day 1 Q2W / Intravenous<br>5-FU / 2600 mg/m2 / 24-hour, day 1 Q2W / Intravenous<br>Leucovorin / 200 mg/m2 / Day 1 Q2W / Intravenous<br>Avelumab / 10 mg/kg / Day 1 Q2W / Intravenous
**Control Intervention**	Not applicable.
**Number of Participants with Rationale:**	To assess the null hypothesis ofH_O: pCR rate ≤ p_O vs. the alternative of H_1: pCR rate ≥ p_1, the sample size is calculated according single-stage phase II design based on the exact binomial distribution. The historical pCR rate is estimated at p_O= 16% (based on FLOT schema as a historical control), and we estimate a promising pCR rate p_1 with the study's treatment at 33%. The study will accrue 30 evaluable patients (modified ITT; patients who undergo surgery) in order to detect such a difference with 82% power using one-sided type I error of 0.1. The null hypothesis will be rejected if at least 8 out of 30 patients achieved a pCR. 37 patients will be recruited considering a 10% drop-out and a potential 10% of screening failures, in order to have at least 30 evaluable patients.
**Study Duration**	Approximately 24 months of recruitment; 5 years of additional follow-up.
**Study Schedule:**	Final Protocol Approved: December 2018<br>First Subject First Visit (FSFV): May 2019<br>Last Subject Las Visit (LSLV): September 2021
**Principal Investigator(s):**	Ignacio Melero<br>CUN Navarra<br>Maria Alsina<br>VHIO
**Study Center(s):**	The trial will be conducted at 10 centers in Spain.

**Statistical Considerations:**	Summary tables (descriptive statistics and frequency tables) will be provided for all demographic, baseline and safety variables, as appropriate. Continuous variables will be summarized with descriptive statistics (mean, standard deviation, range, and median).<br>Ninety-five (95) percent confidence intervals (95% CI) may also be presented, as appropriate. Frequency counts and percentage of subjects within each category will be provided for categorical data.<br>The primary efficacy analysis (pCR) will be performed using the binomial test procedure. Additionally, for the pCR rate one-sided 90% CI will be also presented to be consistent with sample size calculation.<br>Secondary endpoints will be summarized with descriptive statistics.<br>Survival analysis will be performed to analyse OS, PFS and DFS. Kaplan-Meier curves will be presented and possible comparisons will be tested using the log-rank test or the Cox proportional hazard model for multivariate analysis, hazard ratios (HR) and their 95% confidence interval (CI95%) will be provided. Any survival analyses will be considered as exploratory.
**GCP Statement:**	This study will be conducted in compliance with the protocol, the current version of the Declaration of Helsinki, the ICH-GCP or ISO EN 14155 (as far as applicable) as well as all national legal and regulatory requirements.

STUDY SCHEDULE

Table 1. Study schedule

	Screening	Intervention Period				EOT	Follow-up		
Study Periods	**Pre-treatment**	**Pre-operative chemotherapy + Avelumab assessments**	**Pre- operative assessments**	**Post-operative chemotherapy + Avelumab assessments**	**Posterior Avelumab[c]**	**EOT visit**	**Assessments after completion of post-operative chemotherapy + Avelumab**		
							**Safety Follow-up**	**Long-term Follow-up**	
Visits – Time (day/month)	Within 21 days prior to first dose	C1, C2, C3, C4 (FLOT + avelumab) D1 (+/- 3 days) [a, b]	Prior to surgery	C1, C2, C3, C4 (FLOT + avelumab) D1 (+/- 3 days) [a]	C5 to C24 (+/-3 days) [a]	At decision of trial treatment discontinuation (+/-7 days)	Visit (30 days after last dose of avelumab +/-7 days)	3 monthly until 3 years post-surgery +/- 15 days	6 monthly from year 3 until 5 years post-surgery +/- 15 days
Written informed consent	X								
Inclusion/exclusion Criteria	X								
Demographics	X								
Medical History	X								
Physical Examination	X	X	X	X	X	X	X	X	X
Vital signs[1], BP, and oxygen saturation	X	X (only C1)	X	X (only C1)		X	X		
weight (height only at Screening)	X	X	X	X	X	X	X	X	X
12-lead ECG	X		X			X			
ECOG perf.	X	X	X	X	X	X	X	X	X

	Screening	Intervention Period				EOT	Follow-up		
Study Periods	**Pre-treatment**	**Pre-operative chemotherapy + Avelumab assessments**	**Pre- operative assessments**	**Post-operative chemotherapy + Avelumab assessments**	**Posterior Avelumab[c]**	**EOT visit**	**Assessments after completion of post-operative chemotherapy + Avelumab**		
							**Safety Follow-up**	**Long-term Follow-up**	
Visits – Time (day/month)	Within 21 days prior to first dose	C1, C2, C3, C4 (FLOT + avelumab) D1 (+/- 3 days) [a, b]	Prior to surgery	C1, C2, C3, C4 (FLOT + avelumab) D1 (+/- 3 days) [a]	C5 to C24 (+/- 3days) [a]	At decision of trial treatment discontinuation (+/-7 days)	Visit (30 days after last dose of avelumab +/- 7 days)	3 monthly until 3 years post-surgery +/- 15 days	6 monthly from year 3 until 5 years post-surgery +/- 15 days
Concomitant medications	X	X	X	X	X	X	X		
AE and SAE collection[2]	X	X	X	X	X	X	X		
**Intervention**									
Pre-operative chemotherapy (FLOT)		X							
Post-operative chemotherapy (FLOT)				X					
Avelumab		X		X	X				
**Samples and Laboratory Assessments**									
Hematology	X	X	X	X	X	X	X	X	X
Hemostaseology (aPTT, INR)	X	X (only C1)	X	X (only C1)	X (C7,13,19 & 24)	X	X	X	X
Serum chemistry	X	X	X	X	X	X	X	X	X
Urinalysis	X	X (only C1)	X	X (only C1)		X			
Creatine clearance	X	X	X	X		X	X	X	X
β-HCG Pregnancy Test	X	X		X	X	X	X	X[3]	

	Screening	Intervention Period				EOT	Follow-up		
Study Periods	**Pre-treatment**	**Pre-operative chemotherapy + Avelumab assessments**	**Pre- operative assessments**	**Post-operative chemotherapy + Avelumab assessments**	**Posterior Avelumab[c]**	**EOT visit**	**Assessments after completion of post-operative chemotherapy + Avelumab**		
							**Safety Follow-up**	**Long-term Follow-up**	
Visits – Time (day/month)	Within 21 days prior to first dose	C1 C2, C3, C4 (FLOT + avelumab) D1 (+/- 3 days) [a, b]	Prior to surgery	C1, C2, C3, C4 (FLOT + avelumab) D1 (+/- 3 days) [a]	C5 to C24 (+/- 3days) [a]	At decision of trial treatment discontinuation (+/-7 days)	Visit (30 days after last dose of avelumab +/- 7 days)	3 monthly until 3 years post-surgery +/- 15 days	6 monthly from year 3 until 5 years post-surgery +/- 15 days
HBV and HCV test	X								
T4, T3 and TSH	X	X (odd cycles)	X	X (odd cycles)	X (C7,13,19 and 24)	X	X	X	X
Tumor markers (mandatory CEA and CA19-9. Recomm. CA72.4)	X			X (only C1))	X (C7,13,19 and 24)	X		X	X
Tumor tissue (paraffin & frozen)	X [4]		X [4]					X (if relapse)	X (if relapse)
Blood samples for biomarkers		X (previous C1 and C2)	X	X (previous C1 and C2)				X (if relapse)	X (if relapse)
**Tumor evaluation / staging**									
Esophagogastroscopy and histology	X[6]								
[5]CT/MRI chest, abdomen (and pelvis for initial staging)	X		X (no more than 3 weeks before surgery)		X[5]	X[7]		X[5] (every 6 months)	X
EUS-consider If stage T1N0 suspected	X								

	Screening	Intervention Period				EOT	Follow-up		
Study Periods	**Pre-treatment**	**Pre-operative chemotherapy + Avelumab assessments**	**Pre- operative assessments**	**Post-operative chemotherapy + Avelumab assessments**	**Posterior Avelumab[c]**	**EOT visit**	**Assessments after completion of post-operative chemotherapy + Avelumab**		
							**Safety Follow-up**	**Long-term Follow-up**	
Visits – Time (day/month)	Within 21 days prior to first dose	C1, C2, C3, C4 (FLOT + avelumab) D1 (+/- 3 days) [a, b]	Prior to surgery	C1, C2, C3, C4 (FLOT + avelumab) D1 (+/- 3 days) [a]	C5 to C24 (+/- 3days) [a]	At decision of trial treatment discontinuation (+/-7 days)	Visit (30 days after last dose of avelumab +/- 7 days)	3 monthly until 3 years post-surgery +/- 15 days	6 monthly from year 3 until 5 years post-surgery +/- 15 days
Laparoscopy- recommended for T3/T4 tumours	X								
FDG-PET (at the discretion of the investigator)	X								

[a] There is a time window of +/- 3 calendar days for the administration of trial treatment. Also, the assessments required at each timepoint can be performed up to 3 calendar days before treatment administration.

[b] Lab and samples assessments from pre-operative C1D1 can be performed up to 7 calendar days before treatment administration.

[c] After completion of 4 cycles of adjuvant chemotherapy FLOT + avelumab, treatment with avelumab monotherapy will continue for up to 20 more cycles.

[1] Include heart rate, temperature, respiratory rate.

[2] A safety follow-up visit will be scheduled 30 days after the last dose of avelumab. All AEs will be documented until the 30-day Safety Follow-Up visit. After this visit, all SAEs and all treatment-related AEs, especially for the occurrence of new autoimmune events, need to be documented. Subjects with an ongoing SAE must be monitored and followed by the Investigator until stabilization or until the outcome is known, unless the subject is documented as "lost to follow-up. In addition, subjects will be followed for disease progression (CT / MRI scans every 6 months) or survival for up to 5 years after the last patient receives the last dose of avelumab.

[3] β-HCG Pregnancy Test during follow up only to be performed until 6 months after last dose of study treatment.

[4] Frozen tissue if possible

[5] Tumor imaging will be performed at baseline, after the neoadjuvant treatment, after finalizing the adjuvancy with avelumab/FLOT (C5D1 +/- 7days) and then every 6 months until year 5 (+/-7days).

[6] No time limit for the diagnostic esophagogastroscopy and biopsy.

[7] A CT scan should be repeated at time of treatment discontinuation (EOT visit) if it has not been performed on the previous 4 weeks.

1. STUDY ADMINISTRATIVE STRUCTURE

1.1 Sponsor

The Sponsor of this clinical trial is the Vall d'Hebron Institute of Oncology (VHIO).

A contract research organization (CRO), CRS Unit (Clinical Research Support Unit), will undertake the operational aspects of this trial.

1.2 Principal Investigator(s)

Ignacio Melero

Clínica Universitaria de Navarra

Av. de Pío XII, 36, 31008 Pamplona, Navarra, Spain

Maria Alsina

Vall d'Hebron Institute of Oncology

C/ Natzaret, 115-117, 08035 Barcelona, Spain

1.3 Investigational Sites

The trial will be conducted at 10 sites in Spain.

1.4 Laboratory

Blood samples for biomarker analyses will be sent to Clínica Universitaria de Navarra (CUN).

Tumor tissue (frozen and paraffin) will be sent separately to Clínica Universitaria de Navarra (CUN) and to Vall d'Hebron University Hospital (VHIO).

1.5 Monitoring institution

CRS Unit is the monitoring institution that has been assigned.

CRS Unit (Clinical research support Unit), Vall d' Hebron Institute of Oncology (VHIO) Hospital de la Vall d'Hebron

Antiga Escola d'Infermeria, Planta 7

P. Vall d'Hebron 119-129

08035 Barcelona Spain

Tlf. Office: +34 934 893 000 ext. 2432

2. BACKGROUND INFORMATION

2.1 Gastric Cancer

Gastric cancer (GC) and gastroesophageal junction cancer (GEJC) represents a worldwide problem [3]. With about one million cases in 2012, it is currently the fifth most common malignancy and the third leading cause of cancer-related mortality worldwide [4]. Radical surgery remains the gold standard of curative treatment for patients with resectable gastric cancer, but less than the 25% of the patients diagnosed of GC can be considered for resection. Even for patients with localized GC or GEJC the prognosis is poor. In Western countries, the 5-year survival rate of those patients treated with perioperative chemotherapy and surgery is approximately 35-45% [5,6].

GC is a heterogeneous disease, which has been well characterized by different molecular classifications [7-9]. Although being different, these classifications agree on considering the important role of the immune system in the GC tumorogenesis, especially in some subtypes, as the Ebstein-Barr virus (EBV) and the microsatellite instability (MSI) subtypes [7,8]. Moreover, a T-cell inflamed phenotype has been recently demonstrated to be present across all subtypes [10].

In this sense, different immune checkpoints inhibitors [11-18] have shown encouraging activity in refractory GC and GEJC patients, mainly in terms of response rate and overall survival. Although the majority of these studies have been developed in an unselected population, responses are somehow better in those patients with MSI and with higher programmed death ligand-1 protein (PD-L1) levels [17-19]. Furthermore, some immune-gene expression signatures with a correlation with the outcome have been proposed [20,21].

2.2 Immunotherapy and the PD-1/PD-L1 checkpoint pathway

PD-L1 is a member of the B7 family of ligands that inhibit T-cell activity through binding to the programmed cell death-1 (PD-1) receptor [22] and to CD80 [23]. PD-L1 expression is an adaptive response that helps tumours evade detection and elimination by the immune system. Expression of PD-L1 protein is induced by inflammatory signals that are typically associated with an adaptive immune response and can be found on both tumour cells (TC) and tumour-infiltrating IC. The binding of PD-L1 to PD-1 on activated T cells delivers an inhibitory signal to the T cells, preventing them from killing target TC and protecting the tumour from immune elimination [24]. PD-L1 may also inhibit T cells through binding to CD80, although the exact mechanism is still not elucidated [23,25].

The inhibitory mechanism described above is co-opted by tumours that express PD-L1 as a way of evading immune detection and elimination. The binding of an anti–PD-L1 agent to the PD-L1 receptor inhibits the interaction of PD-L1 with the PD-1 and CD80 receptors expressed on immune cells. This activity overcomes PD-L1–mediated inhibition of antitumor immunity.

PD-L1 is expressed in a broad range of cancers. Based on these findings, an anti–PD-L1 antibody could be used therapeutically to enhance antitumor immune responses in patients with cancer. Results of non-clinical and clinical studies of monoclonal antibodies (mAbs) targeting the PD-L1/PD-1 pathway have shown evidence of clinical activity and a manageable safety profile, supporting the hypothesis that an anti–PD-L1 antibody could be used to therapeutically enhance antitumor immune response in cancer patients [26-30] with responses that tend to be more pronounced in patients with tumours that express PD-L1 [31-33].

Currently, there are data from agents in the anti–PD-1/PD-L1 class showing clinical activity in a wide range of tumour types, which are also tested for efficacy in ongoing trials on GC [34].

2.3 Avelumab

Avelumab is an intravenously administered PD-L1 blocking human IgG1 lambda antibody that selectively binds to PD-L1 and competitively blocks its interaction with PD-1. Compared with anti-PD-1 antibodies that target T-cells, avelumab targets tumor cells and is therefore expected to have fewer side effects, including a lower risk of autoimmune-related safety issues, as blockade of PD-L1 leaves the PD-L2/PD-1 pathway intact to promote peripheral self-tolerance [35].

The nonclinical pharmacology investigations have shown that avelumab functionally enhances T-cell activation in vitro and significantly inhibits the growth of PD-L1 expressing tumors in vivo. In agreement with the hypothesis that PD-L1 neutralization acts to release antitumor T cells from immune suppression, the antitumor effects of avelumab in vivo were found to be primarily mediated by CD8+ T cells, as highlighted by the observation that the in vivo depletion of this cell type was sufficient to completely abrogate antitumor activity. Depletion of CD8+ T cells also eliminated the synergistic efficacy of avelumab when given in combination with radiotherapy, suggesting that this combination synergizes through cooperative immune-enhancing mechanisms. As a second mode of action, avelumab is capable of stimulating ADCC activity against PD-L1+ tumor cells in vitro and elimination of ADCC potential in vivo significantly reduced antitumor activity. For complete details of the in vitro and nonclinical trials, please refer to the Investigator's Brochure (IB).

Avelumab is FDA and EMA approved as Bavencio® (Merck KGaA, Darmstadt, Germany) for the metastatic Merkel cell carcinoma (MCC) based on multi-center clinical trial that reported a ORR of 29.5% and a median PFS of 2.6 months, showing that avelumab has a manageable safety profile with durable responses in patients with metastatic MCC who had progressed to chemotherapy [36]. In light of the recent data demonstrating the clinical efficacy of an anti-PD-L1 antibody in advanced gastric cancer, and given the clinical importance of PD-L1 expression in gastric cancer tumor cells [37] and the mode of action of avelumab, avelumab is being developed as a potential therapy for patients with various advanced solid tumors, including gastric cancer.

Data from two Phase I studies of avelumab in patients with GC/GEJC, have been reported to date:

- EMR100070-001: Phase I, open-label, multiple-ascending dose trial to investigate the safety, tolerability, PK, biological, and clinical activity of avelumab in subjects with metastatic or locally advanced solid tumors. [15]. In this trial, avelumab was administered to a large phase 1b cohort of patients with locally advanced or metastatic GC/GEJC unselected for PD-L1 expression. A total of 150 patients were enrolled, including a subgroup of 60 patients who received avelumab as 2L or later treatment. The confirmed ORR was 6.7%. According to the PD-L1 expression status (based on a ≥1% tumor cell cut-off; 73-10 assay), the ORR was 7.7% in PD-L1+ and 3.9% in PD-L1- tumors. In this subgroup, the median OS was 21.4 months.

The JAVELIN Solid Tumor trial also assessed avelumab maintenance treatment in a subgroup of 90 patients with advanced GC/GEJC without disease progression following 1L induction chemotherapy, representing the first study of an anti–PD-1/PD-L1 agent administered as maintenance treatment in any tumor type. The confirmed ORR was 6.7%, the same seen in the 2L subgroup; however, 2.2% of patients in the maintenance subgroup had complete responses, which were not seen in the 2L subgroup. In this subgroup median OS was 6.8 months. Avelumab was associated with an acceptable safety profile across both subgroups, including grade ≥3 TRAEs in 8.7% of patients. [15].

- EMR100070-002: Phase I trial to investigate the tolerability, safety, PK, biological, and clinical activity of avelumab in Japanese subjects with metastatic or locally advanced solid tumors, with expansion part in Asian subjects with GC [38]. Avelumab has also been studied in this phase 1 expansion cohort of Japanese patients with advanced GC/GEJC that progressed after chemotherapy (JAVELIN Solid Tumor JPN). In the 40 patients enrolled in the dose-expansion part, the ORR was 10% and the median OS was 9.1 months. Three of 40 patients had a grade 3 TRAE (7.5%).

Additionally, avelumab is in clinical development with two ongoing Phase III studies in patients with advanced gastric cancer:

- EMR100070-007: Phase III Open-label, Multicenter Trial of Maintenance Therapy With Avelumab (MSB0010718C) Versus Continuation of First-line Chemotherapy in Subjects With Unresectable, Locally Advanced or Metastatic, Adenocarcinoma of the Stomach, or of the Gastro-esophageal Junction [39]. In this clinical trial, avelumab was compared with best supportive care after response or stability to oxaliplatin and fluoropyrimidine, (JAVELIN Gastric 100, NCT2625610). This study did not meet the primary end point of demonstrating superior OS with avelumab maintenance vs continued chemotherapy/BSC. However, avelumab maintenance showed superiority when considering the duration of the response, in patients with no metastatic disease at randomization, and in the exploratory analysis in those PD-L1 CPS ≥ 1 patients. Avelumab also showed a better safety profile.

- EMR100070-008: Phase III Open-label, Multicenter Trial of Avelumab (MSB0010718C) as a Third-line Treatment of Unresectable, Recurrent, or Metastatic Gastric or Gastroesophageal Junction Adenocarcinoma [18]. The aim of this trial was to compare avelumab and BSC vs. paclitaxel or irinotecan and BSC in third-line treatment of AGCThe trial did not meet its pre-specified primary endpoint of superior OS for avelumab vs chemotherapy, but demonstrating similar efficacy in terms of OS with a better safety profile.

Finally, a phase II trial to evaluate the safety and efficacy of administering Avelumab, with cytotoxic FLOT chemotherapy for patients with operable GOA is currently ongoing (Peri-operative Immuno-Chemotherapy in Operable Oesophageal and Gastric Cancer, ICONIC Trial) [40].

This trial is in 2 stages: the first stage will establish the safe and tolerated maximum administered dose (MAD) of avelumab in combination with FLOT and the second stage will assess the efficacy of this combination therapy in achieving pathological complete response (pCR) and peri-operative safety. All patients will receive chemo-immunotherapy consisting of FLOT chemotherapy and the PD- L1 inhibiting monoclonal antibody Avelumab. Four cycles of two-weekly chemo-immunotherapy will be administered before surgery and four further cycles post-operatively in patients who are fit enough to receive further chemo-immunotherapy after surgery.

Refer to the current avelumab IB for a complete summary of non-clinical and clinical information including safety, efficacy and pharmacokinetics.

2.4 Rationale for combination of FLOT plus avelumab

Peri-operative chemotherapy with 5FU, oxaliplatin and docetaxel (FLOT) is a new standard of care in resectable GC or GEJC, and as such, the MONEO study protocol uses the FLOT regimen as standard neoadjuvant chemotherapy.

The FLOT4-AIO phase III trial compared perioperative FLOT chemotherapy with chemotherapy with epirubicin, cisplatin and fluorouracil or capecitabine (ECF/ECX) [2,41]. This trial demonstrated that perioperative FLOT improved outcomes in patients with resectable gastric cancer compared to perioperative ECF/ECX (Median OS 50 mth vs 35 mth, HR 0.77. P=0.012). The 3-year survival rates also favoured the FLOT arm (57% vs. 48%, respectively) [41]. In spite of the benefit of the FLOT schema, there is still a need to further improve outcomes by developing innovative therapeutic combinations.

Combination therapies integrate distinct immunotherapies, including immune checkpoint antagonists and cancer vaccines, with chemotherapy, radiation therapy, and targeted molecular therapy are under active investigation. Albeit cancer chemotherapy has historically been considered immune suppressive, it is now accepted that certain chemotherapies can increase tumour immunity by stimulating immunogenic death.

The demonstrated efficacy of the immune therapies in advanced GC and GEJC patients would probably be translated in the pre-operative setting by increasing the response rate of the tumor, and the percentage of cure. The possibility to combine immune checkpoint inhibitors with chemotherapy would reverse "cold" tumors (those with less peritumoral immune infiltration) to "hot" tumors [42], by a synergistic effect. Chemotherapy may stimulate the innate and the adaptive immune response through different mechanisms: (i) by promoting specific rearrangements on dying tumor cells, which render them visible to the immune system; (ii) by influencing the homeostasis of the hematopoietic compartment stimulating the release of immune cells; (iii) by reverting tumor-induced immunosuppressive mechanisms; and (iv) by exerting direct or indirect stimulatory effects on immune effectors [39,40]. Considering that approximately the 30% of GC and GEJC are thought to have important immune infiltrates within the tumor; the combined strategy would offer the opportunity to rescue the others.

2.5 Rationale for maintenance therapy with avelumab

Surgical treatment alone is inadequate to achieve long-term survival. In spite of the benefit of the perioperative chemotherapy, there is a non-depreciable amount of patients that relapse (either having a pathological response or not). In the FLOT trial, approximately 60% of the patients experienced a relapse within the 5 years after the surgery [41].

Immunotherapy has recently demonstrated a clinical benefit in the adjuvant setting in melanoma [43]. In GC and GEJC, an immunotherapy approach within the year after the surgery is plausible considering the lack of effective treatment options after the completion of the perioperative chemotherapy, and also in the context of the activity demonstrated in the metastatic setting.

2.6 Benefit/risk assessment

2.6.1 Potential benefits

The addition of the anti-PD-L1 therapy avelumab to perioperative chemotherapy may increase pathological responses by a synergic effect activating the immune response. Conclusively, the survival of these patients would improve.

2.6.2 Risks associated with avelumab

Available safety data indicate that avelumab is well tolerated and has a safety profile that is broadly consistent with other anti–PD-1/PD-L1 inhibitors [44-46].

The incidence of grade ≥3 TRAEs with anti–PD-1/PD-L1 monotherapy in patients with GC/GEJC ranges from approximately 10% to 20%, with the most common events including fatigue, anemia, and elevated alanine and aspartate aminotransferase levels. In addition, infusion-related reactions occur in approximately 13% of patients treated with avelumab; these reactions are typically low grade,

occurred after the first or second infusion, and rarely led to discontinuation [44].

Implemented risk mitigation measures for infusion-related reactions/hypersensitivity have been extended by a mandatory premedication with H1 blockers and acetaminophen for all subjects prior first 4 infusions of avelumab. Premedication with an antihistamine and with paracetamol (acetaminophen) approximately 30 to 60 minutes prior to each dose of first 4 infusions of avelumab is mandatory (for example, 25 to 50 mg diphenhydramine and 500-650 mg paracetamol [acetaminophen] IV or oral equivalent). This regimen may be modified based on local treatment standards and guidelines as appropriate.

Checkpoint inhibitor therapy is also associated with immune-related AEs (irAEs) that may affect rheumatic, gastrointestinal, skin, pulmonary, endocrine, neurological, hepatic, cardiac, and renal tissues [47]. In studies of patients with GC/GEJC, the most common grade ≥3 irAEs were pneumonitis and colitis. Compared with the rates of TRAEs with anti–PD-1/PD-L1 monotherapy, higher rates have been associated with anti–CTLA-4 antibodies and combination regimens [48].

Further information on these risks can be found in the current version of the avelumab IB.

2.6.3 Overall benefit-risk

One ongoing study is evaluating the safety of avelumab in combination with FLOT chemotherapy as neoadjuvant therapy to explore potential dose-limiting toxicities or dose reductions of standard therapies that could jeopardize the expected outcome of patients (NCT03399071)[40]. Furthermore, the preliminary results of another trial with a similar schema (NCT03288350) had been already presented at the International Gastric Cancer Congress in May 2019. The authors demonstrated the safety of the combination of avelumab plus the modified DCF (docetaxel, cisplatin, 5-fluorouracil) also in a perioperative setting. The addition of avelumab to standard FLOT chemotherapy can add the specific toxicity of avelumab monotherapy to chemotherapy regimen. For this purpose, a safety interim analysis will be conducted after the first three patients have finalized the first neoadjuvant cycle. Safety of the combination will be confirmed if no related (to the combination) deaths, neither G4 CTCAE toxicities, neither G3 CTCAE toxicities that can be safely managed by the investigator within 3 weeks occur. Drs. Melero and Alsina, together with the PIs of the participant centers, will hold this safety interim analysis.

If the hypothesis of the trial is demonstrated, avelumab may increase not only the primary end point of this trial, the pathological complete response (pCR), but also may increase the long-term benefit of the activation of immune system and increase the disease-free survival (DFS) and the probability to cure the disease (OS).

2.7 Key Point: biomarker analysis

This study has been raised with the idea of a close immune-monitoring. With this strategy, a full

understanding of the effect of avelumab in combination with chemotherapy and the real role of the immune response in the patients will be perfectly assessed.

3. STUDY OBJECTIVES

3.1 Primary Objective

The primary objective is to investigate whether the addition of avelumab to the neoadjuvant chemotherapy (docetaxel, oxaliplatin and fluorouracil/leucovorin) improves efficacy in terms of pathological complete response (pCR) rate, in GC and GEJC patients, compared with the historical controls where neoadjuvant chemotherapy alone was administrated.

3.2 Secondary Objectives

Secondary objectives are as follows:

- To evaluate the addition of avelumab to the perioperative chemotherapy in regard to the following:
    - Overall survival (OS)
    - Disease-free survival (DFS)
    - Progression-free survival (PFS)
    - Surgical complete resection rate (R0)
    - Overall response rate (ORR)
- To determine the safety and tolerability of avelumab with FLOT chemotherapy.
- To perform a comprehensive analysis of biomarkers (exploratory endpoints)

4. STUDY DESIGN

4.1 Overall study design

This is an open-label, non-randomized, multicentric phase II clinical trial in subjects with operable GC or GEJC. The primary objective is to investigate whether the addition of avelumab to the neoadjuvant chemotherapy (docetaxel, oxaliplatin and fluorouracil/leucovorin) leads to improve pCR rate in comparison with FLOT regimen alone. A pathological complete response is defined as the absence of histopathological evidence of viable tumor cells at the site of the original tumor.

Considering a drop-out of 10% and a 10% of screening failure rate, a total of 37 subjects will be enrolled. Treatment will consist on perioperatory treatment of four cycles (each cycle is 14 days) of neoadjuvant chemotherapy (docetaxel, oxaliplatin and fluorouracil/leucovorin) plus avelumab previous to surgery. Surgery is recommended to be scheduled 4 to 6 weeks after the last dose. Afterwards (4 to 10 weeks after surgery), four cycles of adjuvant therapy with the same schema, followed by avelumab alone up to 20 more cycles.

The patient recruitment period of the study will last approximately 24 months. The maximum treatment period for each subject on study includes the pre-operative therapy, the surgical procedure and the post-operative therapy. The primary endpoint will be assessed after the surgery and secondary the endpoints OS, DFS, PFS and long term safety profile will be assessed during each year of follow-up.

A safety interim analysis will be conducted after the first three patients have finalized the first neoadjuvant cycle. Safety of the combination will be confirmed if no related (to the combination) deaths, neither G4 CTCAE toxicities, neither G3 CTCAE toxicities that can be safely managed by the investigator within 3 weeks occur.

Tumor measurements by computed tomography (CT) scan or magnetic resonance imaging (MRI) will be performed at baseline, after the neoadjuvant treatment, and after finalizing the adjuvancy with avelumab/FLOT, and every 6 months thereafter to determine response to treatment. Clinical decision making will be based on Investigator assessment of the scans using RECIST v1.1.

Tissue biopsies (two, pre- and post-neoadjuvant treatment) and collection of blood samples at different time-points during treatment will be processed for the biomarker analyses. Safety of avelumab/FLOT will be monitored continuously by careful monitoring of all adverse events (AEs) and serious adverse events (SAEs) reported.

All left-over material remaining after the predefined translational studies will be stored at VHIO (Barcelona) and CUN (Pamplona). When future analysis will be performed, regulatory approvals will

be obtained.

4.2 Study Schema

The trial design schematic is presented in Figure 1.

Figure 1. Study Design

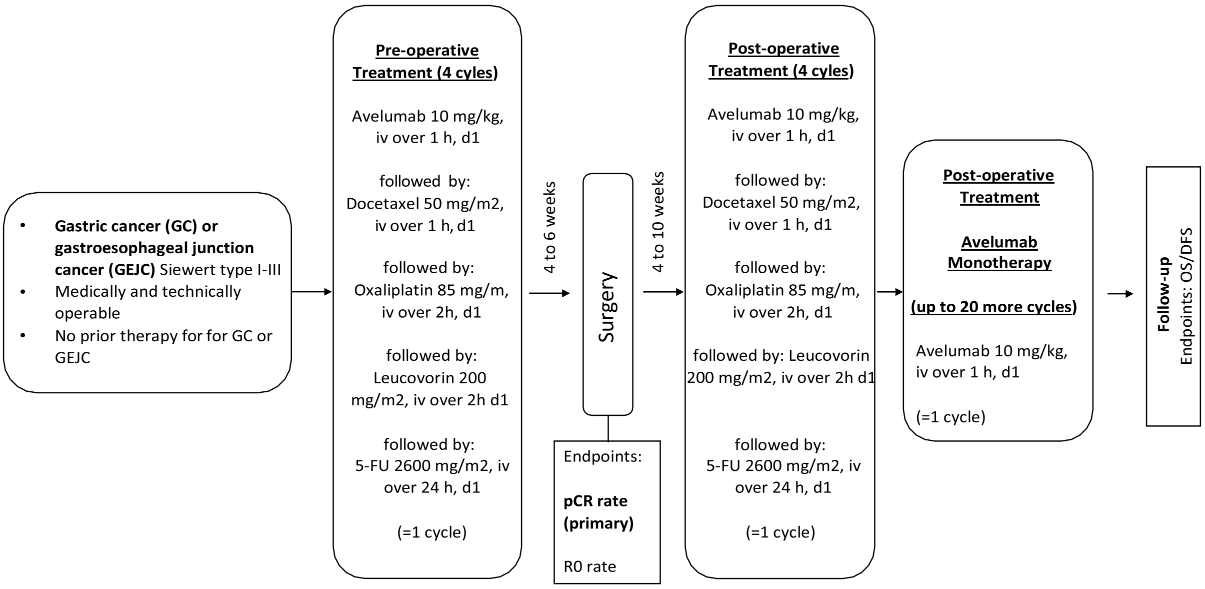

PRE-OPERATIVE TREATMENT

Four cycles of avelumab IV 10 mg/Kg day 1 Q2W with standard dose FLOT chemotherapy (5-Fluorouracil 2600 mg/m² IV 24 h IV day 1 Q2W, Leucovorin 200 mg/m² IV day 1 Q2W, Oxaliplatin 85 mg/m² IV day 1 Q2W, Docetaxel 50 mg/m² IV day 1 Q2W).

SURGERY

Acceptable resections are total gastrectomy, subtotal gastrectomy and esophago-gastrectomy (Ivor-Lewis; for GEJC extending up to 2cm into the lower esophagus). The minimum extent of the operation should be a D1+ lymph node dissection, although a D2 is recommended.

POST-OPERATIVE TREATMENT

Four cycles of avelumab IV 10 mg/Kg day 1 Q2W with standard dose FLOT chemotherapy (5-Fluorouracil 2600 mg/m² IV 24 h IV day 1 Q2W, Leucovorin 200 mg/m² IV day 1 Q2W, Oxaliplatin 85 mg/m² IV day 1 Q2W, Docetaxel 50 mg/m² IV day 1 Q2W). Afterwards, avelumab 10 mg/Kg day 1 Q2W monotherapy up to 20 more cycles (total adjuvancy=24 cycles).

5. STUDY POPULATION

Subjects who meet the inclusion and not meet the exclusion criteria will be eligible for participation in this study. Prior to performing any trial assessments not part of the subject's routine medical care, the Investigator will ensure that the subject or the subject's legal representative has provided written informed consent following the procedure described in Section 9.1.2.

5.1 Inclusion criteria

1. Histologically proven, gastric or GEJ adenocarcinoma (Siewert I-III).
2. Availability of two paraffin blocks from the diagnostic endoscopic biopsy (and a fresh biopsy if possible), and another tumor block (paraffin) from the surgical specimen. In some sites, a fresh tumor sample will be required.
3. Have evaluable disease as defined by RECIST 1.1 and determined by investigator assessment, with the absence of distant metastases on CT scan of thorax, abdomen and pelvis.
4. Patient medically fit and amenable to gastrectomy/esophagectomy with curative intent as confirmed by a multidisciplinary team discussion.
5. UICC tumor stage Ib (T1N1 only, T2N0 not eligible) to IIIC, as defined by CT, according to the 7th AJCC Edition.
6. Age ≥ 18 years.
7. WHO performance status 0-1.
8. Adequate organ function (assessed within 7 calendar days prior treatment initiation):
    a. White blood cell count (WBC) ≥ 3 x 10^9 /L
    b. Absolute neutrophil count (ANC) ≥ 1.5 x 10^9 /L
    c. Platelets ≥ 100 x 10^9 /L
    d. Estimated glomerular filtration rate should be ≥ 50 ml/min
    e. Total bilirubin within normal limits (if the patient has documented Gilbert's disease ≤ 1.5 x ULN or direct bilirubin ≤ ULN).
    f. Aspartate transaminase (AST) and alanine transaminase (ALT) ≤ 2.5 x ULN.
9. In case of anticoagulation, investigator and patient should agree to replace any oral anticoagulation by subcutaneous administration of low-molecular weight heparin in equivalent doses before treatment start;
10. For women who are not postmenopausal (> 12 months of non-therapy induced amenorrhea) or surgically sterile (absence of ovaries and/or uterus): agreement to remain abstinent or use

single or combined contraceptive methods that result in a failure rate of < 1% per year during the treatment period and for at least 12 months after the last treatment dose

11. For men: agreement to remain abstinent or use a condom plus an additional contraceptive method that together result in a failure rate of < 1% per year during the treatment period and for at least 12 months after the last dose of study treatment. Abstinence is only acceptable if it is in line with the preferred and usual lifestyle of the patient. Periodic abstinence (e.g. calendar, ovulation, symptothermal, or postovulation methods) and withdrawal are not acceptable methods for contraception.

12. For all female patients who are not confirmed postmenopausal (> 12 months of non-therapy induced amenorrhea) or surgically sterile (absence of ovaries and/or uterus) a negative serum pregnancy test (β-human chorionic gonadotropin [β-hCG]) result should be available before treatment and within 7 days from treatment start should be performed. Female patients should not be breast feeding.

13. Written informed consent must be given according to ICH/GCP, and national/local regulations.

5.2 Exclusion criteria

The presence of any one of the following exclusion criteria will lead to exclusion of the participant:

1. Other histology different from adenocarcinoma.
2. Has had previous therapy for gastric or GEJ cancer.
3. Known hypersensitivity to the components of anti-PD-L1, docetaxel, oxaliplatin, fluorouracil/leucovorin.
4. Known dihydropyrimidine dehydrogenase (DPD) deficiency.
5. Previous malignancy within the last 5 years, except for adequately treated cervical carcinoma in situ, localized non-melanoma skin cancer, or other curatively treated cancer without impact on the patient’s overall prognosis according to the judgment of the investigator.
6. Any psychological, familial, sociological or geographical condition potentially hampering compliance with the study protocol and follow-up schedule; those condition should be discussed with the patient before registration in the trial.
7. History of clinically significant comorbidities.
8. Patients medically unfit for FLOT chemotherapy, according to the local guidance.
9. Active autoimmune disease that has required systemic treatment in past 2 years (i.e. with use

of disease modifying agents, corticosteroids or immunosuppressive drugs). Replacement therapy (e.g., thyroxin, insulin, or physiologic corticosteroid replacement therapy for adrenal or pituitary insufficiency, etc.) is not considered a form of systemic treatment.

10. Diagnosis of immunodeficiency or is receiving systemic steroid therapy or any other form of immunosuppressive therapy within 7 days prior to the first dose of trial treatment. Current use of immunosuppressive medication, EXCEPT for the following: a. intranasal, inhaled, topical steroids, or local steroid injection (e.g., intra-articular injection); b. Systemic corticosteroids at physiologic doses ≤ 10 mg/day of prednisone or equivalent; c. Steroids as premedication for hypersensitivity reactions (e.g., CT scan premedication). History or evidence of interstitial lung disease or active, non-infectious pneumonitis.
11. Active infection requiring systemic therapy.
12. Known history of Human Immunodeficiency Virus (HIV) (HIV 1/2 antibodies) or Active Hepatitis B (e.g., HBsAg reactive) or Hepatitis C (e.g., HCV RNA [qualitative] is detected). Test for HBV and HCV are required for the screening.
13. Received a live vaccine within 30 days of planned start of study therapy. Note: Seasonal influenza vaccines for injection are generally inactivated flu-vaccines and are allowed; however intranasal influenza vaccines are live attenuated vaccines, and are not allowed.
14. Prior organ transplantation including allogenic stem-cell transplantation.
15. Known prior severe hypersensitivity to investigational product or any component in its formulations, including known severe hypersensitivity reactions to monoclonal antibodies (NCI-CTCAE v4.0 Grade ≥ 3).
16. Persisting toxicity related to prior therapy (NCI-CTCAE v4.0 Grade > 1); however, alopecia, sensory neuropathy Grade ≤ 2, or other Grade ≤ 2 not constituting a safety risk based on investigator's judgment are acceptable.
17. Other severe acute or chronic medical conditions including immune colitis, inflammatory bowel disease, immune pneumonitis, pulmonary fibrosis or psychiatric conditions including recent (within the past year) or active suicidal ideation or behaviour; or laboratory abnormalities that may increase the risk associated with study participation or study treatment administration or may interfere with the interpretation of study results and, in the judgment of the investigator, would make the patient inappropriate for entry into this study.
18. Pregnant women and lactating females are excluded from this study.

5.3 Criteria for withdrawal / discontinuation of participants

In consenting to this trial, patients agree to receive study treatment, attending trial follow-up,

assessment and data collection. If a patient expresses the wish to withdraw from the trial treatment, site staff should explain the importance of maintaining follow-up.

A patient may withdraw, or be withdrawn, from trial treatment for the following reasons:

- Unacceptable toxicity
- Disease Progression
- Intercurrent illness which prevents further follow-up
- Investigator discretion
- Withdrawal of consent for treatment by patient

In this case sites must be clear on what the patient's intentions are, does the patient wish to withdraw from the trial treatment and procedures however allow follow-up data to be collected or does the patient wish to withdraw not only from the trial treatment and procedures but also from providing follow-up data. Patients may re-consent if they change their mind following withdraw to resume participation on this trial.

6. TREATMENT OF SUBJECTS

6.1 Treatment plan schedule

Subjects will receive **four pre-operative cycles of FLOT plus avelumab (8 weeks)** at two-weekly intervals pre-operatively (surgery should be performed within 4 to 6 weeks of the end of the last cycle) and **four post-operative cycles of FLOT plus avelumab (8 weeks)** (should be commenced between 4 to 10 weeks after surgery). Subjects then will continue to receive avelumab monotherapy for up to 20 more cycles (total adjuvant treatment cycles=24), every two weeks (figure 2).

Figure 2. Treatment schedule

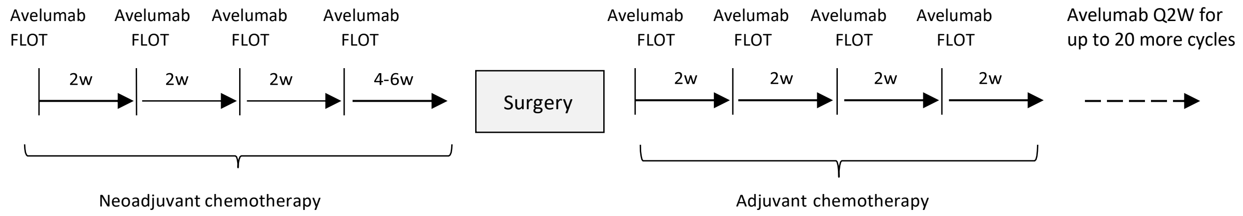

FLOT: 5-fluorouracil, oxaliplatin, docetaxel, leucovorin

6.2 Dosage and administration

6.2.1 Avelumab dosage, administration and pre-medication

Avelumab drug product is a sterile, clear, and colorless concentrate for solution intended for intravenous (IV) infusion. The drug is presented at a concentration of 20 mg/mL in single-use glass vial containing 200 mg of avelumab.

Subjects will receive an IV infusion of avelumab at a dose of 10 mg/kg (over the duration of 1 hour).Premedication with an antihistamine and with paracetamol (acetaminophen) approximately 30 to 60 minutes prior to each dose of avelumab is highly recommended (for example, 25 to 50 mg diphenhydramine and 500 to 650 mg paracetamol [acetaminophen] IV or oral equivalent). This regimen may be modified based on local treatment standards and guidelines as appropriate.

The dose of avelumab will be calculated based on the weight of the subject determined within 72 hours prior to the day of drug administration. The dose of avelumab used for the previous

administration can be repeated if the change in the subject's weight is 10% or less than the weight used for the last dose calculation.

Subjects will receive avelumab followed by FLOT chemotherapy once every two weeks for four cycles before surgery and for four cycles post-operatory. Then, treatment with avelumab will continue every two weeks up to one year.

Avelumab will be stopped if confirmed disease progression per RECIST v1.1, significant clinical deterioration (clinical progression), unacceptable toxicity, withdrawal of consent, or if any criterion for withdrawal from the trial or trial treatment is fulfilled.

6.2.1.1 Special precautions

Immediate access to an intensive care unit or equivalent environment and appropriate medical therapy (including epinephrine, corticosteroids, IV antihistamines, bronchodilators, and oxygen) must be available for use in the treatment of infusion-related reactions. Infusion of avelumab will be stopped in case of Grade ≥ 2 infusion-related, allergic, or anaphylactoid reactions. Following first avelumab infusion, subjects must be observed for 2h post infusion for potential infusion- related reactions. The observation period for the following avelumab infusions can be modified at the investigator's discretion, but an observation period of 2h is also required in the first avelumab administration without pre-medication (in case investigator decides to stop administering pre-medication at some point).

As with all monoclonal antibody therapies, there is a risk of allergic reaction. Avelumab should be administered in a setting that allows for immediate access and administration of therapy for severe allergic/hypersensitivity reactions, such as the ability to implement immediate resuscitation measures. Steroids (dexamethasone 10 mg), epinephrine (1:1000 dilution), allergy medications (antihistamines), or equivalents should be available for immediate access.

If hypersensitivity reaction occurs, the subject must be treated according to the best available medical practice. Guidelines for management of infusion-related reactions and severe hypersensitivity and flu-like symptoms according to the NCI are found in Sections 6.3.3. A complete guideline for the emergency treatment of anaphylactic reactions according to the Working Group of the Resuscitation Council (United Kingdom) can be found at https://www.resus.org.uk/pages/reaction.pdf. Subjects should be instructed to report any delayed reactions to the Investigator immediately.

6.2.2 FLOT chemotherapy dosage and administration

Table 2. FLOT Therapy Regimen Dose definition and number of cycles

**Product Description**	**Dosage Form**	**Dose**	**Dosing Frequency**
5-Fluorouracil (5-FU)	IV	2600 mg/m²	Over 24 hours on Day 1 every 2 weeks for 4 cycles pre and post-operatively
Leucovorin	IV	200 mg/m²	On Day 1 every 2 weeks for 4 cycles pre and post-operatively

Oxaliplatin	IV	85 mg/m²	On Day 1 every 2 weeks for 4 cycles pre and post-operatively
Docetaxel	IV	50 mg/m²	On Day 1 every 2 weeks for 4 cycles pre and post-operatively

Dose calculation of all chemotherapy drugs is based on body surface area. The preparation and dose calculation of all chemotherapy drugs will be performed as per site common practice. The body surface area will be calculated by using the actual body weight. Patients with a BSA in excess of 2.2m2 will be capped at a BSA of 2.2 m^2.

In the event of a weight change of ≥ 10%, treatment doses should be recalculated. There will be no intrapatient dose escalation.

Subjects will receive trial treatment until confirmed disease progression per RECIST v1.1, significant clinical deterioration (clinical progression), unacceptable toxicity, withdrawal of consent, or if any criterion for withdrawal from the trial or trial treatment is fulfilled.

6.2.2.1 Pre-treatment considerations

Insertion of a central venous access device (CVAD). A CVAD (i.e. PICC line or Infusaport) is strongly recommended for use with the FLOT regimens.

6.2.2.2 Administration of FLOT chemotherapy regimen

The administration of FLOT chemotherapy regimen will be as follows:

- Administer docetaxel in 250ml NaCl 0.9% over 1 hour.
- Administer oxaliplatin in 500ml glucose 5% over 2 hours.
- Administer Leucovorin in 250ml NaCl 0.9% over 2 hours.
- Commence 5-fluorouracil via an infusion pump as a continuous intravenous infusion over 24 hours.

Similar schemas of treatment can be considered if they are the standard of care in each center, previous consultation to the sponsor.

6.2.2.3 Anti-emetic recommendations

- Dexamethasone 8mg PO Day -1 to prevent fluid retention and allergic reactions.
- Dexamethasone 8mg IV Day 1 and then 8mg PO days 2 to 4, for both anti emetic effects and to prevent fluid retention
- A 5-HT3 Antagonist IV or PO (dose specified below).

- similar schemas of antiemetics can be considered if they are the standard of care in each center, previous consultation to the sponsor

Table 3. Doses of 5-HT3 Antagonists

Ondansetron - Zofran	IV - 8mg day 1 / Oral - 8mg bd for 5 doses
Granisetron - Kytril	IV - 3mg* day 1 / Oral - 2mg days 2 and 3
Dolasetron - Anzemet	IV - 100mg day 1 / Oral - 200mg days 2 and 3
Tropisetron - Navoban	IV - 5mg day 1 / Oral - 5mg days 2 and 3

Additional anti-emetics (such as aprepitant) may be given with moderate emetogenic risk chemotherapy regimens as per local instutional practice but are not mandatory.
Administration of IV 5-HT3 Antagonists

- Dolasetron - give as a slow IV over 30 sec or dilute to 50mL sodium chloride 0.9% and infuse over 15 min.
- Granisetron - dilute in 20 - 50mL sodium chloride 0.9% and infuse over 5 min.
- Ondansetron - give as a slow IV over 2-3 min or dilute in 100mL sodium chloride 0.9% and infuse over 15 min (doses greater than 8mg should be infused to decrease visual disturbances).
- Tropisetron - give as a slow IV over 2-3 min or dilute in 100mL sodium chloride 0.9% and infuse over 15 min.

Combination preparations of 5-HT3 antagonists and NK1 receptor antagonists (e.g. netupitant/palonosetron) may be used if available.

6.3 Toxicity management guidelines

6.3.1 Toxicity and grading criteria

All toxicities will be graded by investigators according to the National Cancer Institute- Common Terminology Criteria for Adverse Events (NCI-CTCAE) version 4.0 (November 27, 2017).

6.3.2 FLOT chemotherapy toxicity management

6.3.2.1 Expected effects and approximate times that these effects will be seen

Infusional 5FU:

a) Emesis: throughout exposure, b) Mucositis: from 7-10 days, c) Diarrhoea: from 7-10 days, d) Myelosuppression: Within 7-10 days, e) Hand-foot syndrome: Cumulative from 4-8 weeks, f) Photosensitivity, g) Coronary artery spasm.

Note: patients with dihydropyrimidine dehydrogenase (DPD) deficiency are at risk of increased and potentially fatal toxicity

<u>Oxaliplatin</u>

a) Anaphylaxis: immediate, b) Emesis: within 12 hours, c) Cold-induced parasthesiae: within 24 to 48 hours, d) Laryngo-pharyngeal dysaesthesia: within 24 to 48 hours, e) Myelosuppression: within 7-10 days, f) Mucositis: from 7-10 days, g) Diarrhoea: from 7-10 days, h) Peripheral neuropathy: cumulative from 4-8 weeks.

<u>Docetaxel</u>

a) Emesis: within 12 hours, b) Mucositis: from 7-10 days, c) Diarrhoea: from 7-10 days, d) Myelosupression: Within 7-10 days, e) Skin rash, f) Arthralgia and myalgia, g) Peripheral edema, h) Peripheral neuropathy, i) Skin reaction, j) Nail disorder.

6.3.2.2 Dose delay and modification. General remarks

Instructions for treatment delays and dose modifications for adverse events are specified below and in Appendix 1. Adverse events will be graded according to NCI-CTCAE v4.0.

In general, toxicities of severity grade 1 only will not lead to any dose reduction or cycle delay. All study treatment (FLOT and Avelumab) should be withheld during adverse events of severity Grade 3-4 that are related to any of the FLOT drugs, and should not be restarted until the adverse event has resolved to Grade 0-2, except where otherwise specified. Day 1 treatment may be delayed for a maximum of 28 days. If the adverse event has not resolved to Grade 0-2 after delaying day 1 treatment for 28 days, then the causative drug should be permanently discontinued (all FLOT components or only the one(s) related to the AE). Treatment should not be delayed or modified for alopecia of any grade.

If commencement of a planned cycle (i.e.: Day 1 of treatment) is delayed, the first day of treatment of that cycle is still referred to as Day 1.

In case of toxicity requiring dose modification, the dose modification should reflect the causal relationship to the respective drug(s). E.g., if the toxicity is unequivocally caused by only one drug, a dose modification of other drugs is not required. If a patient experiences several adverse events with differing recommendations, then the most conservative modification (i.e.: results in the longest delay and lowest dose) should be used. Specified dose reductions apply to all subsequent doses of study drug unless otherwise specified.

In case of acute allergic reactions of grade 3 or 4, the causative agent should be discontinued permanently. In case of grade 1 or 2, it is up to the physician to continue treatment without dose modification, if this is in the best interest of the patient.

Recommendation for dose modifications for FLOT chemotherapy associated toxicities are provided in

Appendix 1 (Section 11.1). Nevertheless, dose modification for FLOT chemotherapy can be done by per local practice.

6.3.3 Avelumab toxicity management

6.3.3.1 General remarks

Subjects receiving avelumab must be observed for 2 hours post infusion on the first administration, in an area with resuscitation equipment and emergency agents. For the following administrations, the observation period can be modified at the investigator's discretion, but an observation period of 2h is also required in the first avelumab administration without pre-medication (in case investigator decides to stop administering pre-medication at some point). At all times during avelumab or maintenance chemotherapy treatment, immediate emergency treatment of an infusion-related reaction or a severe hypersensitivity reaction according to institutional standards must be assured. In order to treat possible hypersensitivity reactions, for instance, dexamethasone 10 mg and epinephrine in a 1:1000 dilution or equivalents should always be available along with equipment for assisted ventilation.

Infusion of avelumab will be stopped in case of Grade ≥ 2 hypersensitivity, inflammatory response, or infusion-related reaction. The treatment recommendations for infusion-related reactions, severe hypersensitivity reactions, and tumor lysis syndrome according to the NCI are as outlined in Appendix 1 (Sections 11.2.1, 11.2.2 and 11.2.3, respectively).

Investigators should also monitor subjects closely for potential immune-related adverse events (irAEs), which may first manifest after weeks of treatment. Such events may consist of persistent rash, diarrhea and colitis, autoimmune hepatitis, arthritis, glomerulonephritis, cardiomyopathy, or uveitis and other inflammatory eye conditions. The spectrum of hypothetical irAEs also includes formation of auto-antibodies like antinuclear antibodies (ANAs) or antineutrophil cytoplasmic antibodies (ANCAs). Refer to Appendix 1 (Section 11.2.4) for details on the management of irAEs.

Avelumab treatment may be withheld due to toxicity for a maximum of 90 days. If treatment cannot be resumed by then, avelumab should be permanently discontinued.

6.3.3.2 Dose modifications for avelumab toxicities

Dose modifications for avelumab treatment associated toxicities are provided in Appendix 1 (Section 11.2).

6.4 Distribution, packaging and labeling of the Investigational Medicinal Product

The investigational Medicinal Product in this trial is avelumab only. As docetaxel, oxaliplatin and 5-fuorouracil/leucovorin are generally available and established for the routine treatment of gastric cancer, they will be prescribed by the treating physician, as this prescription is within the

framework of standard usage.

Avelumab is formulated as a 20 mg/mL solution and is supplied by the Sponsor in single-use glass vials, stoppered with a rubber septum and sealed with an aluminum polypropylene flip-off seal.

All IMPs will be packaged and labeled in accordance with all applicable regulatory requirements and Good Manufacturing Practice Guidelines. Avelumab will be packed in boxes each containing 1 vial. The information on the trial treatment will be in accordance with approved submission documents.

Avelumab will be shipped in transport cool containers (2°C to 8°C) that are monitored with temperature control devices.

FLOT chemotherapy agents will be supplied by the study center, according to local laws and regulations.

6.5 Preparation, Handling, and Storage of the Investigational Medicinal Product

The contents of the avelumab vials are sterile and nonpyrogenic, and do not contain bacteriostatic preservatives. Any spills that occur should be cleaned up using the facility’s standard cleanup procedures for biologic products.

Avelumab must be stored at 2°C to 8°C until use, with a temperature log maintained daily. All medication boxes supplied to each trial site must be stored carefully, safely, and separately from other drugs.

Avelumab stored at room temperature (23°C to 27°C) or at elevated temperatures (38°C to 42°C) for extended periods is subject to degradation. Avelumab must not be frozen. Rough shaking of avelumab must be avoided.

For application in this trial, avelumab must be diluted with 0.9% saline solution (sodium chloride injection).Alternatively a 0.45% saline solution can be used if needed. It is recommended that the diluted avelumab solution is used immediately. If not used immediately, the diluted drug product can be stored up to 8 hours at room temperature or up to 24 hours at 2°C to 8°C. Detailed information on infusion bags and medical devices to be used for the preparation of the dilutions and subsequent administration will be provided in the IPMP.

Avelumab must not be used for any purpose other than the trial. The administration of avelumab to subjects who have not been enrolled into the trial is not covered by the trial insurance.

Any unused portion of the solution should be discarded in biohazard waste disposal with final disposal by accepted local and national standards of incineration.

Storage, handling, preparation, and disposal of IMP should be according to local institutional

guidelines.

6.6 Investigational Medicinal Product Accountability

The Investigator is responsible for ensuring accountability for avelumab and maintenance chemotherapy, including reconciliation and maintenance of drug records.

- Upon receipt of trial treatment, the Investigator (or designee) will check for accurate delivery and acknowledge receipt by signing (or initialing) and dating the documentation provided by the Sponsor and returning it to the Sponsor. A copy will be retained for the Investigator Site File.
- The dispensing of the trial treatment will be carefully recorded on the appropriate drug accountability forms provided by the Sponsor and an accurate accounting will be available for verification by the clinical research associate (CRA) at each monitoring visit.

Trial treatment accountability records will include:

1. Confirmation of trial treatment delivery to the trial site.
2. The inventory at the site of trial treatment provided by the Sponsor and prepared at the site
3. The use of each dose by each subject
4. The disposition of unused trial treatment
5. Dates, quantities, batch numbers, expiry dates and (for trial treatment prepared at the site) formulation, as well as the subjects' trial numbers.

- The Investigator should maintain records that adequately document

1. That the subjects were provided the doses specified by the clinical trial protocol/amendment(s)
2. That all trial treatment provided by the Sponsor was fully reconciled.

Unused trial treatment must not be discarded or used for any purpose other than the present trial. Any trial treatment that has been dispensed to a subject must not be re-dispensed to a different subject.

The CRA will periodically collect the trial treatment accountability forms and will check all returns (both unused and used containers) before authorizing their destruction by the trial site.

At the conclusion or termination of this trial, trial site personnel and the CRA will conduct a final product supply inventory on the investigational drug accountability forms and all unused containers will be destroyed. Instructions for destruction of product will be provided to the site. The clinical trial monitor will be supplied with a copy for filing of the investigational drug

accountability forms. This documentation must contain a record of clinical supplies used, unused, and destroyed and shall include information on:

- All administered units
- All unused units
- All destroyed units (during the trial)
- All destroyed units at the end of the trial
- Date of destruction(s)
- Name and signature of the Investigator/pharmacist.

It must be ensured at each trial site that the trial treatment is not used:

- After the expiry date
- After the retest date unless the trial treatment is reanalyzed and its retest date extended.

This is to be closely monitored by the Clinical Trial Monitor.

6.7 Concomitant Medications and Therapies

All concomitant medications taken by the subject during the trial, from the date of signature of informed consent are to be recorded in the appropriate section of the eCRF, noting the name, dose, duration, and indication of each drug. Nondrug interventions and any changes to a concomitant medication or other intervention should also be recorded in the eCRF.

6.7.1 Permitted Medicines

Any medications (other than those excluded by the clinical trial protocol) that are considered necessary to protect subject welfare and will not interfere with the trial treatment may be given at the Investigator’s discretion.

Other drugs to be used for prophylaxis, treatment of hypersensitivity reactions, and treatment of fever or flu-like symptoms.

Anti-nauseant and dexamethasone pre-medication and other supportive medications (i.e. therapy for pain, mucositis etc) will be as per institutional practice.

The Investigator will record all concomitant medications taken by the subject during the trial, from the date of signature of informed consent, in the appropriate section of the eCRF.

Any additional concomitant therapy that becomes necessary during the trial and any change to concomitant drugs must be recorded in the corresponding section of the eCRF, noting the name, dose, duration, and indication of each drug.

Rescue medications may be administered to address ineffective treatment, anticipated adverse reactions, or anticipated emergency situations.

Medicinal products known to prolong the QTc interval must be used with caution in subjects receiving oxaliplatin.

Growth factors (granulocyte colony stimulating factor or granulocyte macrophage colony stimulating factor) and erythropoietin and darbepoetin alpha may be prescribed at the Investigator's discretion

6.7.2 Prohibited Medicines

As stated for the exclusion criteria in Section 5.2, subjects must not have previous therapy for gastric or GEJ cancer. In addition, the following treatments must not be administered during the trial:

- Concurrent immunotherapy or cytotoxic chemotherapy other than defined by the protocol, or other experimental pharmaceutical products. Short-term administration of systemic steroids (that is, for allergic reactions or the management of irAEs) is allowed.

If the administration of a non-permitted concomitant drug becomes necessary during the trial, the subject will be withdrawn from trial treatment (the Sponsor may be contacted to discuss whether the trial treatment must be discontinued).

Medications other than those specifically excluded in this trial (see above) may be administered for the management of symptoms associated with the administration of avelumab or chemotherapy as required. These might include analgesics, anti-nausea medications, antihistamines, diuretics, antianxiety medications, and medication for pain management, including narcotic agents.

Any additional concomitant therapy that becomes necessary during the trial and any change to concomitant drugs must be recorded in the corresponding section of the eCRF, noting the name, dose, duration, and indication of each drug.

6.8 Surgical Considerations

It is strongly recommended that patients who are considered to have a primary tumour that is T3 / T4 should have a diagnostic laparoscopy to exclude overt peritoneal metastasis. If peritoneal washings are performed, positive washings without evidence of peritoneal metastasis are not an exclusion from the trial. The decision to participate or not will be at the discretion of the treating clinicians.

Following completion of the neoadjuvant therapy, patients should be restaged with CT of the chest and abdomen. If there is no evidence of systemic disease and the patient is considered fit for surgery, a resection should occur 4-6 weeks from the completion of the neoadjuvant therapy.

6.8.1 Gastric resection

The acceptable resections are a total gastrectomy, a distal subtotal gastrectomy or an esophago-

gastrectomy (Ivor-Lewis esophago-gastrectomy for GEJ cancers extending up to 2cm into the lower oesophagus). It is considered that the operation of choice should be a D2 gastrectomy but the minimum approach is a D1+ gastrectomy aiming for complete resection of the primary cancer and its draining nodes (defined below). There should be a gastric margin of 5cm. For proximal lesions there should be 3cm margin including the esophageal margin. For distal lesions there should be a minimum of 2cm of the duodenum resected.

Cancers involving the lower esophagus requiring a thoracotomy should have a minimum esophageal margin >2cm. A frozen section is encouraged where clinical concern for margin involvement. The thoracotomy may be in association with a total gastrectomy and extended oesophageal resection or as an Ivor-Lewis esphago-gastrectomy with a laparotomy and right thoracotomy. For a gastrectomy, the greater omentum should be removed enbloc in the resection. The resection may include adjacent organs if they are considered to be involved with disease. Splenectomy and resection of the pancreatic tail are not expected as a routine and will be performed in relation to the site of the tumour and an adequate resection. Primary cancers at the fundus or upper half of the greater curve should have a splenectomy to ensure the N1 nodes (station 10) are adequately cleared.

Splenectomy is only recommended for tumours that involve the upper greater curve/fundus of the stomach or if the distal pancreas/splenic hilum are involved with disease. Pancreatic resection is only required if the distal pancreas is directly involved with the tumour.

If an R0 resection is not possible the operating surgeon will decide on the appropriate management for the patient.

An R0 resection is not considered possible if:

- There is tumour infiltration into the head of the pancreas.
- N3 nodal metastasis (refers to Japanese nodal classification and not TNM staging)
- Peritoneal metastasis if not directly adjacent to the primary, that can not be included in the resection.

6.8.2 Extent of Lymphadenectomy

Resection Definitions – According to Japanese Research Society for Gastric Cancer Classification 2010

Distal Gastrectomy:

D1: 1,3,4d,4sb,5,6,7

Minimum requirement – D1+: as above, 8a, 9

D2: as above, 12, 11p (dissection to posterior gastric artery)

<u>Total Gastrectomy:</u>

D1: 1,2,3,4sa,4sb,4d,5,6,7

Minimum requirement – D1+: as above, 8a,9,11p

D2: as above, 10,11d,12a (Station 10 will include splenectomy if cancer on the fundus or upper third of the greater curve)

<u>Esophago-gastrectomy for GEJ cancer into the lower 2cm of esophagus:</u>

D1: 1,2,3,4sa,4sb,7

Minimum requirement – D1+: as above, 8a,9,11p,19,20,111

Figure 3. Gastric Lymph Node Stations

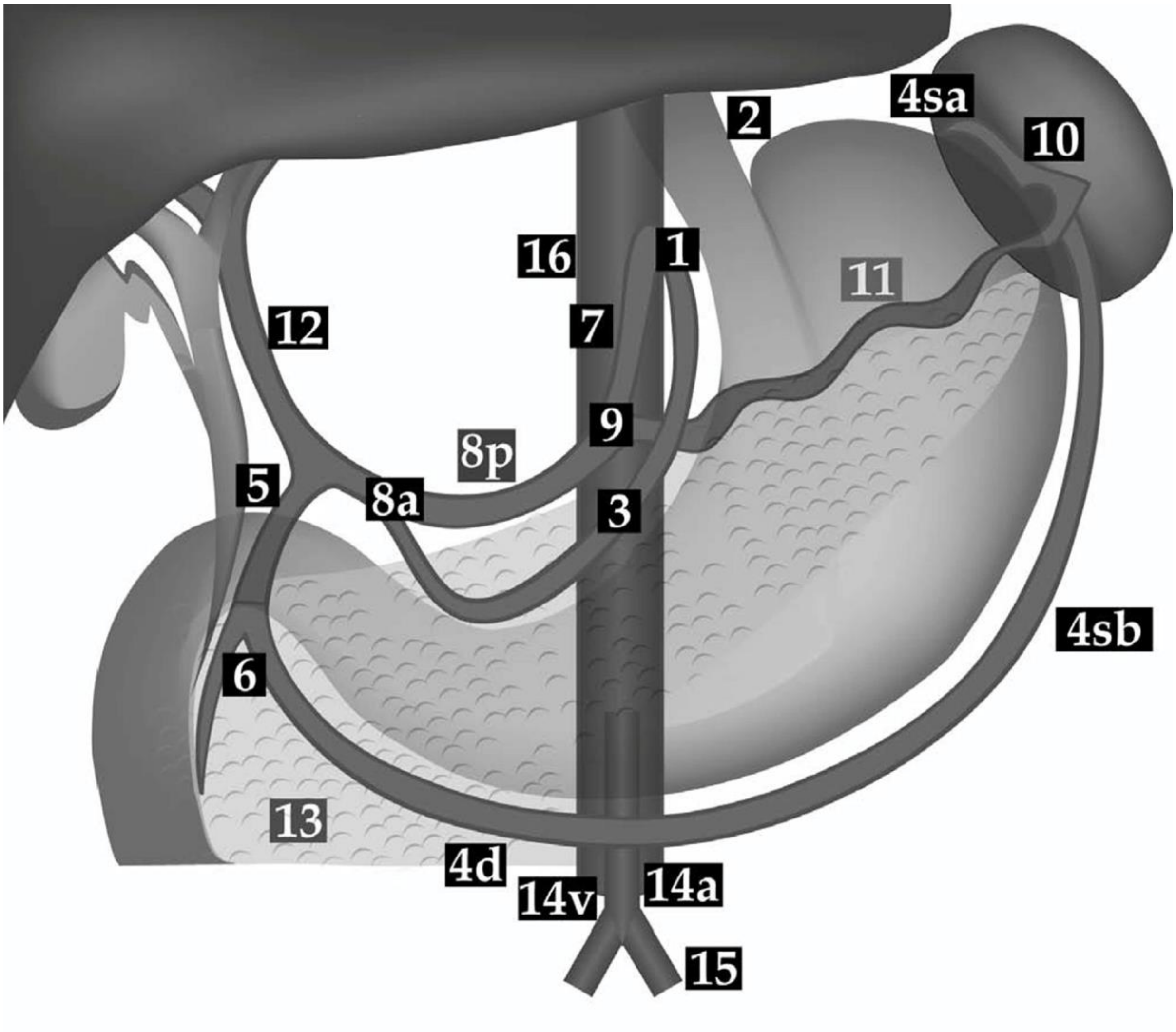

<u>Gastric Lymph Node Stations</u>

1. Right cardio-esophageal
2. Left cardio-esophageal
3. Lesser curve

4sa. Short gastric
4sb. Left gastroepiploic
4d. Right gastroepiploic
5. Suprapyloric
6. Infrapyloric
7. Left gastric
8a. Common hepatic (anterior)
8p. Common hepatic (posterior)
9. Coeliac axis
10. Splenic hilum
11d. Splenic arterial – distal
11p. Splenic arterial - proximal
12. Hilar
13. Retropancreatic
14a. Superior mesenteric arterial
14v. Superior mesenteric venous
15. Transverse mesocolon
16. Para-aortic

Nodes relevant to esophago-gastrectomy

19. Infradiaphramatic
20. Esophageal hiatus
110. Lower para-esophageal
111. Supra diaphagragmatic
112. Posterior mediastinal

6.8.3 Surgical Quality Control

Each surgeon who agrees to participate in the trial will need to have reviewed the surgical protocol. In addition, each surgeon will complete an operation form (as per local guidance) which will indicate the extent of surgery, the lymph node stations resected and the reconstruction.

In addition, the pathology will be reviewed and used as a surrogate for adequacy of resection. It is expected that at least 15 lymph nodes will be resected during a radical gastrectomy for carcinoma.

6.8.4 Operative Technique

Description of the advised methodology, although equivalent local practices are acceptable.

Peritoneal Lavage: Following a laparotomy, a lavage with saline is done to take samples for a cytology testing. 200mls of Normal Saline are placed into the peritoneal cavity and 10 mls are aspirated and sent for cytology.

A Duodenal Kocher maneuver is performed to evaluate the lymph node stations 13 and 16 behind the pancreas and the aortic region. If the nodes are suspicious they should be sent for frozen section. If involved, the resection will be considered palliative.

An omentectomy should be performed (unless esophago-gastrectomy). The omentum is taken off the transverse colon. In general there is no vascular connection between the anterior and posterior layer of the mesocolon of the transverse colon. Therefore, these sheets may be separated without any bleeding. The anterior sheet should remain ‘en bloc’ with the greater omentum. The dissection is continued to the splenic flexure. The left gastroepiploic vessels are ligated close to the origin on the splenic vessels.

To the right the dissection approaches the root of the mesentery to nodal station 6 and for a distal gastrectomy dissection of station 14v. The right gastroepiploic vein and artery are ligated taking the tissue around them. The posterior pylorus is defined as is the gastro-duodenal artery leading to the station 5 nodes (these nodes need to be removed for a Distal gastrectomy / D1)

To the left the anterior sheet of the mesocolon extends to the pancreas to allow the omental bursa resection.

After division of the gastro-hepatic ligament, along the liver and ligation of the right gastric artery, the tissue medial the hepatic artery proper is removed (station 12). The duodenum is now divided. The stomach is retracted to the left.

For a Distal gastrectomy / D2 resection stations 5, 8a, 9 and 11p (to the origin of the posterior gastric artery) are dissected from right to left and from the pancreas towards the root of the left gastric artery. When the retroperitoneal tissue around the celiac axis is cleared and the left gastric artery is divided

the dissection of this region is completed. The tissue to the right of the cardia (node station 1) is dissected enbloc with the left gastric tissue (node station 3, 7)

For a Total Gastrectomy / D2 resection the dissection the posterior gastric branch is ligated and station 11d is dissected. The short gastric vessels are ligated and the fundus mobilised which will include node station 2.

For tumours of the upper and mid greater curve the spleen should be removed (stations 4sa and 10 will be removed).

If pancreaticosplenectomy is to be performed, mobilisation of the pancreas body and tail following the division of the mesocolon sheets will enhance the facility of splenectomy in a later stage. Complete mobilisation of the spleen is done after the lymph node dissection of the celiac axis region is completed. The entire specimen of stomach, spleen and pancreas can be lifted from the retroperitoneum, leaving the left adrenal gland in situ. Division of the pancreas is done at the level of the inferior mesenteric vein or celiac axis. The pancreas is clamped, divided and the duct is ligated after which the stump is closed.

For patients with a carcinoma of the cardia or gastroesophageal junction that require a thoracic approach to resect the esophagus, the proximal third of the stomach should be resected after ligation of the left gastric vessels and preserving the right gastric and right gastro-epiploic arteries. This will include stations 1,2,3,4sa,4sb and 7 for a D1 dissection. The addition of stations 8a,9,11p,19 and 20 (the latter two around the hiatus) make this D1+. Because this approach implies lower esophageal involvement the fat pad above the diaphragm should be included in the resection (station 111). This can be performed from below the diaphragm (having opened the hiatus) or from within the thoracic component of the operation.

The thoracic component will typically be completed using a right sided thoracotomy with resection of the lower esophagus. Typically the resection is taken to at least the level of the azygous vein but this will be at the discretion of the surgeon. The creation of the gastric tube, following resection of the upper third of the stomach, and the anastomosis will be at the discretion of the treating surgeon.

6.8.5 Postoperative procedures

6.8.5.1 Reconstruction

Following a total or partial gastrectomy, reconstruction with a Roux en Y loop of non-irradiated jejunum to make an anastomosis to the esophagus or proximal stomach is the recommended technique. It will be up to the surgeon to choose his preferred reconstruction and the technique of closing it (hand sutured or stapled).

Following an Ivor-Lewis, esophago-gastrectomy the reconstruction of choice is a gastric tube

anastomosed to the esophagus in the thorax. However the formal reconstruction and anastomosis technique is at the discretion of the operating surgeon.

Also the use of a feeding jejunostomy and the management of a naso-gastric (jejunal) tube decompression is at the surgeon's discretion.

6.8.5.2 Pathological Aspects

Prior to sending the specimen to the Pathology department, the proximal and distal aspects of the resection specimen, as well as the lesser and greater curvatures of the stomach, should be clearly identified by sutures and/or appropriate diagrams. Any lymph nodes not removed 'en bloc' with the specimen should be placed in appropriately labelled containers to ensure that their specific lymph node location is clear to the pathologist. These procedures will assist the receiving pathologist in orienting the specimen, and in the correct identification of nodal stations and margins. Surgeons should continue the routine practice in their institution. It would be desirable to dissect out individual lymph node stations but is accepted that this may not be practical.

6.9 Subject Follow-up

Participation in this study is voluntary; subjects may withdraw from treatment or from the entire study at any time. Subjects who stop study treatment prior to the time recommended in the protocol will continue follow-up visits according to the protocol. Every effort must be made to contact all patients for their follow-up visits. If a patient is unable to be contacted by local study coordinators, then their general practitioner, medical records, and other national databases may be used to ascertain vital status. Patients will provide written/oral informed consent to permit access to their health information.

6.9.1 Extended safety follow-up

Given the potential risk for delayed immune-related toxicities, safety follow-up must be performed within the first 30 days after the last dose of avelumab administration.

Long-term follow-up visits will occur until 5 years after the surgery (3 monthly until 3 years; and 6 monthly from Year 3 until 5 years).

7. STUDY PROCEDURES AND ASSESSMENTS

7.1 Schedule of assessments

A complete Schedule of Assessments for the Screening period and treatment is provided in Table 1.

Prior to performing any trial assessments not part of the subject's routine medical care, the Investigator will ensure that the subject or the subject's legal representative has provided written informed consent according to the procedure described in Section 9.2.

7.1.1 Pre-treatment evaluation:

To be performed within 21 days prior to study enter unless otherwise specified.
Required assessments/tasks:

- Patient Information and written Informed consent
- Demographics data, medical history and physical examination, body weight, height, blood pressure, oxygen saturation by pulse oximetry at rest and after exertion, and vital signs (heart rate, temperature, respiratory rate)
- ECOG Performance status
- 12-lead ECG
- Hematology, coagulation, full serum chemistry, urinalysis, β-HCG Pregnancy Test, HBC and HCV test, T4/T3 and TSH.
- Tumor markers (mandatory CEA and CA 19-9. Recommended CA72.4)
- Creatinine clearance
- Fresh frozen (FF) tumor sample and formalin-fixed, paraffin-embedded (FFPE). Only FFPE is acceptable when FF tissue is not available.
- Review of prior/concomitant medications.

Tumour staging investigations:

There is no time limit for the diagnostic esophagogastroscopy and biopsy. All other staging investigations that are used to confirm eligibility of patient for the study should be performed within 21 days prior to first dose.

- Staging investigations include:

- CT scan chest, abdomen and pelvis
- Laparoscopy is strongly recommended for patients with T3/T4 primary tumours.

- FDG-PET scanning should be performed at the investigator discretion.
- Endoscopic ultrasound (EUS) should be considered where available and performed if a stage 1a tumour is suspected (patient excluded if confirmed).

7.1.2 Assessments prior to starting C1 of FLOT chemotherapy plus avelumab (pre-operative and post-operative; day 1, or up to 3 calendar days before the cycle).

- Physical examination, body weight, blood pressure, oxygen saturation by pulse oximetry at rest and after exertion, and vital signs (heart rate, temperature, respiratory rate) and ECOG performance status.
- Hematology, full serum chemistry, coagulation, urinalysis and T4, T3, TSH. *Note: before C1 of neoadjuvant treatment, this assessment can be performed up to 7 calendar days before C1D1.*
- Blood samples for biomarkers.
- ECOG performance status
- AE and SAE collection. Investigators assess the toxicity grades according to NCI-CTCAE v4.0 Toxicity will be scored on the basis of the worst grade experienced in the preceding cycle to assess suitability to continue to the next cycle, including before first cycle to assess baseline toxicities.
- Record all concomitant medication use.

7.1.3 Assessments prior to starting C2, C3 and C4 of FLOT chemotherapy plus avelumab (pre-operative and post-operative; day 1, or up to 3 calendar days before the cycle).

- Physical examination, body weight
- ECOG performance status
- Hematology, serum chemistry , T4, T3 and TSH (C3 pre and post-operative)
- Blood sample for biomarkers (only prior to C2)
- AEs and SAEs collection and toxicity assessment (NCI – CTCAE v4.0)
- Record all concomitant medication use.

7.1.4 Assessments prior to surgery:

Surgery should be performed within 4 to 6 weeks of the end of the last FLOT plus avelumab cycle.

- Physical examination, body weight, blood pressure, oxygen saturation by pulse oximetry at rest and after exertion, and vital signs (heart rate, temperature, respiratory rate).
- ECOG Performance status
- 12-lead ECG

- Hematology, full serum chemistry, coagulation, urinalysis, T4, T3 and TSH
- AEs and SAEs collection and toxicity assessment (NCI-CTCAE v4.0)
- Tumour Restaging:

Following completion of preoperative treatment, patients should be restaged to ensure that there are no signs of progressive disease and that the primary tumour is deemed resectable. **Restaging should be performed with CT of the chest and abdomen. This assessment should be carried out regardless of whether the patient continues on protocol treatment.**

Other investigations such as esophagogastroscopy, laparoscopy, EUS and PET can be performed as clinically indicated.

Patients should also undergo anaesthetic assessment for surgery.

7.1.5 Assessments during avelumab (Post-operative, post FLOT chemotherapy)

- Physical examination and body weight
- ECOG Performance status
  - Hematology, coagulation (C7, 13, 19 and 24), serum chemistry. T3, T4 and TSH (C7, C13, C19 and C24)
  - Tumor markers (mandatory CEA and CA 19-9. Recommended CA72.4) should be performed on C7, C13, C19 and C24.
  - AEs and SAEs collection and toxicity assessment (NCI-CTCAE v4.0)
  - Record all concomitant medication use.
  - Tumor imaging will be performed after finalizing the adjuvancy with avelumab/FLOT and then every 6 months until year 5.

7.1.6 End of treatment

An End of treatment (EOT) visit should be performed at time when it is decided that the patient discontinues the study treatment. There is a time window of +/- 7 calendar days to perform this visit. The visit will include the following assessments:

- Physical examination, body weight, blood pressure, oxygen saturation by pulse oximetry at rest and after exertion, and vital signs (heart rate, temperature, respiratory rate)
- ECOG Performance status
- 12-lead ECG
- Hematology, coagulation, full serum chemistry, urinalysis, β-HCG Pregnancy Test, T4/T3 and

TSH.

- Creatinine clearance
- Tumor markers (mandatory CEA and CA 19-9. Recommended CA72.4)
- AEs and SAEs collection and toxicity assessment (NCI-CTCAE v4.0)
- Record all concomitant medication use
- Tumor imaging, unless there has been an imaging assessment in the last 4 weeks prior to treatment discontinuation.

7.1.7 Safety Follow-up

After the completion of the therapy, all subjects will have subsequent Safety Follow-up Visits approximately every 3 months (during the first 3 years after surgery), and then every 6 months (up to 5 years after surgery).

The visit will include the following full assessment of safety parameters:

- Physical examination and body weight
- ECOG Performance status
- Hematology, coagulation full serum chemistry, T3, T4 and TSH
- AEs and SAEs collection and toxicity assessment (NCI-CTCAE v4.0)
- Record all concomitant medication use

Safety Follow-up call must be monitored and followed by the Investigator until stabilization or until the outcome is known, unless the subject is documented as “lost to follow-up”. Any SAE assessed as related to must be reported whenever it occurs, irrespective of the time elapsed since the last administration of IMP.

7.1.8 Non-compliance with pre-op FLOT and/or post-op FLOT

Analysis will be by intention to treat. If non-compliant with treatment, follow-up data will continue to be collected on these patients. Data collected will include treatment received and disease status, responses and recurrence dates.

7.1.9 Schedule of Assessments

The trial Schedule of Assessments are presented in *Table 1*.

7.2 Demographic and Other Baseline Characteristics

7.2.1 Demographic Data

At Screening, the following demographic data will be collected: date of birth, sex (gender), race, and ethnicity.

7.2.2 Diagnosis of Gastric Cancer

The tumor disease information that will be documented and verified at the Screening visit for each subject includes

- Detailed history of the tumor, including pathological diagnosis, histology, grading, and staging in accordance with the International Union Against Cancer Tumor Node Metastasis Classification of Malignant Tumors at diagnosis;
- All therapy used for prior treatment of the tumor (including surgery, radiotherapy and chemotherapy, immunotherapy);
- Current cancer signs and symptoms

7.2.3 Medical History

In order to determine the subject's eligibility to the trial, a complete medical history of each subject will be collected and documented during Screening, which will include, but may not be limited to, the following:

- Past and concomitant diseases and treatments.
- All medications (including herbal medications) taken and procedures carried out within 21 days prior to Screening.

For the trial entry, all of the subjects must fulfil all inclusion criteria described in Section 5.3.1, and none of the subjects should have any exclusion criterion from the list described in Section 5.3.2.

7.2.4 Vital Signs and Physical Examination

Vital signs including body temperature, respiratory rate, heart rate (after 5-minute rest), arterial blood pressure (after 5-minute rest) and oxygen saturation by pulse oximetry at rest and after exertion will be recorded at trial entry.

A complete physical examination (including, in general, appearance, dermatological, head/neck, pulmonary, cardiovascular, gastrointestinal, genitourinary, lymphatic, musculoskeletal system, extremities, eyes, nose, throat, and neurologic status) will be performed and the results documented.

The ECOG Performace status, body weight and height, and 12-lead ECG will be assessed and performed at screening.

7.2.5 Clinical Laboratory Assessments

The following clinical laboratory tests will be performed (see the Schedule of Assessments for the

timepoints of each test):

Table 4. Hematology Laboratory Tests

Absolute lymphocyte count	White blood cell count and differential count
Absolute neutrophil count	Red blood cell (RBC) morphology
Hematocrit	Reticulocytes
Hemoglobin	Mean corpuscular haemoglobin (MCH)
Platelet count	Mean corpuscular volume
Red blood cell (RBC) count	Mean corpuscular haemoglobin concentration (MCHC)

Table 5. Serum chemistry Laboratory Tests

Albumin	Glucose
Alkaline phosphatase	Lactate dehydrogenase (LDH)
Alanine aminotransferase (ALT)	Lipase
Amylase	Phosphorus/Phosphates
Aspartate aminotransferase (AST)	Magnesium
gamma-glutamyltransferas (GGT)	Potassium
BUN/total urea	Sodium
Calcium	Total bilirubin
Chloride	Total protein
Cholesterol	Uric acid
Creatinine Kinase	Triglycerides
Creatinine	
CRP	

Table 6. Urinalysis Tests *

Bilirubin	Urobilinogen
Blood	pH
Glucose	Protein
Ketones	Specific gravity
Leukocyte esterase	Colour and appearance
nitrite	

* Microscopic evaluation should be performed if considered appropriate per local practice)

Table 7. Coagulation Tests

Activated partial thromboplastin time (aPTT)	Prothrombin time/INR

7.2.6 CT or MRI Scans for Tumor Assessment at Baseline

Baseline imaging will be performed to establish baseline disease status of target and non-target lesions according to RECIST v1.1.

Acceptable modalities include CT scans (chest, abdomen, and pelvis), CT chest together with MRI of the abdomen and pelvis, or positron emission tomography/CT scans. The use of IV contrast is preferred unless there is a history of allergy or other risk in the opinion of the Investigator (chest X-ray is not acceptable and other imaging modalities may be performed at the discretion of the Investigator and as clinically indicated). Baseline tumor burden should be determined. In general, lesions detected at Screening/baseline need to be followed using the same imaging methodology and preferably the same imaging equipment at subsequent tumor evaluation visits.

7.3 Efficacy Assessments

7.3.1 Pathological complete response rate

Pathological complete response (pCR) rate, where pCR is defined as the absence of residual tumor based on evaluation of the resected esophagogastric specimen according to Becker remission criteria [1].

Pathological assessment

The pathologist will evaluate the tumour for histopathological type, grade, localization in the stomach, size, depth of invasion, evidence of vascular or lymphatic invasion, resection margins, lymph node metastases (including number assessed, number involved and location would be desirable but not an absolute requirement), and the degree of tumour necrosis. Histological type and grade will be determined according to 2000 WHO classification, and stage will be determined according to AJCC/UICC TNM guidelines (7th Edition).

The pathologist should be made aware of all clinical details relevant to the management of the case. Information regarding prior treatment of the tumour, and radiographic findings relevant to tumour response should be provided to the pathologist when requested, or where these are relevant to an understanding of tissue changes present in the resection specimen.

Microscopic assessment will be performed on the basis of review of hematoxylin-and-eosin-stained sections from formalin-fixed and paraffin-embedded tissue blocks. Macroscopic sampling of the tumour, or site of tumour, should be performed so as to demonstrate the full extent of possible tumour involvement. Where a pathologic complete response is suspected, the pathologist should ensure that the affected area of the stomach is fully sampled to ensure absence of residual tumour.

To determine the response of the tumour to neoadjuvant therapy, the surgical specimen should be assessed both macroscopically and microscopically for the extent of viable tumour cells, and the extent of tumour regression, as evidenced by non-viable tumour, fibrosis and/or tumour cell-free mucin deposits.

Pathological extent of original tumour and residual viable tumour

The pathologist should report the greatest cross sectional area of viable tumour within the specimen, in terms of actual area (mm2), or by reporting size in two dimensions (mm). The pathologist should also estimate (if possible) the size of the original tumour prior to neoadjuvant therapy by identifying areas of probable tumour regression. It is recognised that this assessment can only be an estimate of the actual pre-treatment tumour size. Determination of Pathological Response (PR).

Pathological response refers to the extent of reduction in tumour size following preoperative treatment, as determined by macroscopic and microscopic assessment of the tumour. It will be based on the pathological assessment of the original tumour size and the extent of residual viable tumour, as described above. Pathological response was scored using the criteria of Becker et al [1].

Table 8. Pathological response criteria according to Becker scoring

Grade 1a	0% residual tumour	Complete response
Grade 1b	< 10% residual tumour per tumour bed	Subtotal tumour regression
Grade 2	10 - 50% residual tumour per tumour bed	Partial tumour regression
Grade 3	> 50% residual tumour per tumour bed	Minimal or no tumour regression

Central Pathology Review

Central pathology reviews will take place to standardise the evaluation of the gastrectomy specimen. Once reviewed, the slides will be returned to the patient's local pathology laboratory. The Reviewer will issue a report which will be sent to the investigator and the Trial Centre.

7.3.2 Assessment of secondary outcomes

- Overall survival (OS)

[time frame: from the initial date of neoadjuvant avelumab- chemotherapy to the date of death due to any cause. Patients without documentation of death at the time of analysis will be censored at the last follow-up date]. Estimated using Kaplan-Meier method.

- Disease free survival (DFS)

Disease-free survival (DFS) [time frame: from the surgery to the first observation of disease relapse or death due to any cause. Patients without an event prior to the time of analysis will be censored at the

last relapse-free assessment]. Estimated using Kaplan-Meier method. Relapse will be determined according to RECIST v1.1 assessed as per investigator assessment.

- Progression-free survival (PFS)

Progression-free survival (PFS) [time frame: from the initial date of neoadjuvant avelumab-chemotherapy treatment to the date of first documentation of disease progression or death due to any cause, whichever occurs first. Patients without an event prior to the time of analysis will be censored at the last assessment that is stable disease (SD) or better]. Estimated using Kaplan-Meier method.

Progression is defined according to RECIST v1.1 assessed as per investigator assessment.

- Surgical complete resection (R0)

This is complete macroscopic resection of gross tumor with negative surgical margins. The margins assessed include proximal (esophageal, gastric), distal (gastric for proximal resection or duodenum) and lateral margins (posterior cardia, GEJ, esophageal and nodal tissue on the lesser curve) of the specimen. The margin will be deemed involved (R1 resection) if there is carcinoma seen at or within 1mm of the margin. It will be deemed uncertain if there are malignant cells within 10mm of the margin and clear otherwise. The presence of carcinoma at a true serosal surface will be noted but not considered an involved surgical margin. The R0 resection rates will be compared with historical cohorts.

- Clinical Overall Response Rate (ORR)

The clinical overall response rate (ORR) is defined as the proportion of all subjects achieving complete response (CR) or partial response (PR) according to Response Evaluation Criteria in Solid tumors (RECIST) v1.1 and as adjudicated by the local investigator. The CR was defined as the disappearance of all target lesions (TLs) and the non-target lesions (NTLs). PR is defined as at least a 30% decrease in the sum of the longest diameter (LD) of TLs, taking as reference baseline sum LD.

7.3.3 Assessment of exploratory outcomes

- Pathological immune response (pIR)
    - The pathological immune response is the parameter that evaluates the effect of the immunotherapy not only by measuring the response of the tumor cells (ypTNM), but also the response of the immune system. Type and amount of the lymphocyte infiltrate will be evaluated using the Galon et al. immunoscore [49]. This analysis of the immunoprofile in the tumor area will likely identify other prognostic markers that may be histology dependent.
- Characterization of the immune contexture

  - The immune contexture may yield relevant information for prognosis and prediction of the treatment response. Determined of the density, composition, functional state and organization of the leukocyte infiltrate of the tumor, as well as the tumor microsatellite instability and mutational gene signatures will be explored,

- Immunodynamic follow-up
  - Despite this clinical efficacy of anti-PD1 and anti-PDL1 strategies, systemic and intratumoral immune effects are not clearly correlated with systemic dose, toxicity and efficacy. Thus, the conventional assessment with standard pharmacokinetics and pharmacodynamics seems not to be useful for the evaluation of immune strategies. The immunodynamic follow-up does take into account the changes that occur in the blood, by measuring the type of cytokines and TILs, and within the tumor, by evaluating the immune infiltrate [50].

- TCR clonality assessment
  - Lymphocyte maturation is a fascinating process marked by immunophenotypic changes, as well as discrete and regulated molecular events. An important part of this molecular 'evolution' involves somatic alteration of the germline configuration of the T cell receptor (TCR) genes to a unique configuration, in order to permit development of a clone of T cells with an extracellular receptor specific to a given antigen. In this sense, T cell clonality testing has important clinical and research value, because it can provide specific and reproducible assessment of clonal diversity in T cell proliferations [51].

7.4 Assessment of Safety

The safety profile of the trial treatments will be assessed through the recording, reporting, and analyzing of Baseline medical conditions, AEs, physical examination findings, including vital signs, and laboratory tests.

Comprehensive assessment of any apparent toxicity experienced by the subject will be performed throughout the course of the trial, from the time of the subject's signature of informed consent. Safety follow-up will be done in the periodical assessments defined in Section 7.1.6.

Trial site personnel will report any AE, whether observed by the Investigator or reported by the subject (see Section 7.4.6).

7.4.1 Definitions

Adverse Event

An Adverse Event (AE) is any untoward medical occurrence in a patient or clinical investigational subject administered a pharmaceutical product and which does not necessarily have a causal relationship with this treatment. An AE can therefore be any unfavourable or unintended sign

(including an abnormal laboratory finding), symptom, or disease temporally associated with the use of a medicinal investigational product, whether or not considered related to the medicinal product. An adverse event is any adverse change (developing or worsening) from the patient's pre-treatment condition, including intercurrent illness.

Serious Adverse Event

A Serious Adverse Event (SAE) or serious adverse reaction (SAR) is any untoward medical occurrence that at any dose:

- Results in death.
- Is life-threatening (Note: The term "life-threatening" refers to an event in which the subject is at risk of death at the time of the event, not an event that hypothetically might have caused death if it was more severe).
- Requires inpatient hospitalisation or prolongation of existing hospitalisation (Note: Hospitalisation regardless of length of stay, even if the hospitalisation is a precautionary measure for continued observation. Hospitalisations for a pre-exisiting condition, including elective procedures that have not worsened, do not constitute an SAE).
- Results in persistent or significant disability or incapacity.
- Is a congenital anomaly/birth defect.
- Is an important medical event*.

*Note: Medical and scientific judgement should be exercised in deciding whether expedited reporting is appropriate in cases of important medical events that may not be immediately life- threatening or result in death or hospitalisation but may jeopardise the subject or may require intervention to prevent one of the other outcomes listed in the definition above. These should also usually be considered serious.

Examples of such events are intensive treatment in an emergency room, or at home for allergic bronchospasm; blood dyscrasias or convulsions that do not result in hospitalisation; or development of drug dependency or drug abuse, or malignant tumours when they are histologically different from the primary tumour.

There are exceptions for the TOPGEAR trial only, see section 6.2.2 for further information.

Adverse drug reaction

An adverse drug reaction (ADR) is All noxious and unintended responses to a medicinal product related to any dose should be considered adverse drug reactions. The phrase 'responses to a medicinal product' means that a causal relationship between a medicinal product and an adverse event is at

least a reasonable possibility, i.e. the relationship cannot be ruled out.

Suspected unexpected serious adverse reaction (SUSAR)

A SUSAR is a SAR that is classified as 'unexpected' i.e. a SAR the nature or severity of which is not consistent with the information about the medicinal product in question set out in the summary of product characteristics or investigational brochure for that product. All SUSARs related to the study drugs will be reported to the regulatory authorities by the CTC. A SUSAR may include events related to chemotherapy, immunotherapy or surgery.

7.4.2 Events not to be treated as SAEs

Due to the seriousness of the disease in this study and the similarity of protocol treatments to standard care, certain conditions/events defined as SAEs will be excluded from expedited reporting as SAEs:

- Progression of disease is not to be regarded as an SAE
- Death due to progressive disease is not to be regarded as an SAE
- Elective hospitalisation and/or surgery for treatment of gastric cancer or its complications
- Elective hospitalisation to simplify treatment or study procedures
- Events that are unrelated to any of the study treatments (including chemotherapy, immunotherapy or surgery)
- Events that are related to study treatments (including chemotherapy, immunotherapy or surgery) but expected, unless an adverse event of special interest as listed below.

*Note: events that are unrelated to study drugs, but are believed to be related to the surgery or radiation therapy must be treated as SAEs and reported in an expedited fashion as noted below, although these may not require reporting to the local regulatory authorities.

7.4.3 Adverse Events of Special Interest and Pregnancy

A number of adverse events of special interest have been identified. These include:

- Cardiac disorders grades 3 and above
- Specified surgical complications:
  - Leak: anastomotic
  - Re-operation
  - Sepsis ≥ grade 3

In addition, in the event of a pregnancy occurring during the course of the study, the event must be reported and the subject must be withdrawn from study drug immediately. Pregnancies occurring up

to 6 months after the completion of the study drug must also be reported to the investigator. The investigator should counsel the patient, discuss the risks of continuing with the pregnancy and the possible effects on the foetus. The subject must be followed during the entire course of the pregnancy and postpartum period. Parental and neonatal outcomes must be recorded even if they are completely normal.

Pregnancy occurring in the partner of a patient participating in the study and up to 90 days after the completion of the test drug should also be reported. The partner should be counselled and followed as described above.

While the specified adverse events of special interest and pregnancy may not meet the criteria for SAEs, they should be reported in an expedited fashion, as noted below.

7.4.4 Collection and Recording of Adverse Events, incl. SAEs and Adverse Events of Special Interest and Pregnancy

All AEs must be documented in the appropriate section of the CRF by the investigator. SAEs, adverse events of special interest and pregnancies will be reported through CRF (W3NEXUS) immediately after their awareness.

The NCI-CTCAE v4.0 will be used to classify and grade the intensity of adverse events and their relationship to study drug administration.

The following aspects must be recorded for each event in the CRF:

For all adverse events;

- A description of the AE in medical terms according to NCI-CTCAE v 4.0, not as reported by the subject;
- The grade as assessed by the investigator according to the definitions in NCI-CTCAE v 4.0:
    - Grade 1 = mild
    - Grade 2 = moderate
    - Grade 3 = severe
    - Grade 4 = life-threatening or disabling
    - Grade 5 = death related to AE

In addition, the following aspects must also be recorded for all SAEs, adverse events of special interest and pregnancy;

- The date of onset (start date)
- The date of recovery (stop date)

- The causal relationship to the study treatment (chemotherapy, immunotherapy, surgery) as assessed by the investigator; the decisive factor in the documentation is the temporal relation between the AE and the study treatment. The following judgements of the causality to study drug are to be used:
  - Unrelated = There is not a temporal relationship to study treatment administration (too early, too late, or study treatment not administered), or there is a reasonable causal relationship between another drug, concurrent disease, or circumstance and the AE.
  - Related: It is at least possible that the event is related to the study treatment.
- The expectedness of the event, pertaining to whether the event is consistent with the expected side-effect profile of the study treatment, as judged by the investigator and with reference to the current investigator brochures and product information. Events which are consistent with the known side-effect profile, but which are associated with an unexpected outcome should be classified as unexpected.
- Action taken on study treatment (ceased, interrupted, dose reduced, no action).
- The outcome according to the following definitions:
  - Recovered
  - Recovered with sequelae
  - Ongoing
  - Death
- Seriousness: yes or no (If yes, SAE report needs to be completed)

In case of SAEs it must be indicated whether the SAE is the leading event, i.e. the primary medical reason for SAE reporting.

If in any one subject the same AE occurs on several occasions, then the AE in question must be documented and assessed anew each time.

7.4.5 Definition of the Adverse Event Reporting Period

Adverse Events and serious adverse events will be reported from the time of signature of informed consent until 30 days following the last dose of any drug from the protocol treatment schedule or until the start of subsequent systemic therapy for the disease under study, if earlier.

Any SAE assessed as related to the trial treatment must be reported whenever it occurs, irrespective of the time elapsed since the last administration.

During the course of the study all AEs and SAEs should be proactively followed up for each patient. Every effort should be made to obtain a resolution for all events, even if the events continue after discontinuation/study completion. It is the responsibility of the investigator that any necessary additional therapeutic measures and follow-up procedures are performed.

7.4.6 Procedure for Reporting of Serious Adverse Events, Adverse Events of Special Interest and Pregnancy

In the event of any new SAE, adverse events of special interest and Pregnancies occurring during the reporting period, the Investigator must immediately (within a maximum 24 hours after becoming aware of the event) inform the Sponsor or its designee using the CRF platform.

The Investigator must respond to any request for follow-up information (for example, additional information, outcome, final evaluation, other records where needed) or to any question the Sponsor/designee may have on the AE within the same timelines as those noted above for initial reports. This is necessary to ensure prompt assessment of the event by the Sponsor or designee and (as applicable) to allow the Sponsor to meet strict regulatory timelines associated with expedited safety reporting obligations.

7.4.7 Safety Reporting to Health Authorities, Independent Ethics Committees / Institutional Review Boards and Investigators

Safety reporting:

The Sponsor-Investigator primary responsibilities for safety reporting are to identify and follow-up on Serious Adverse Events (SAEs) experienced by participants in the study and to forward the information to the local regulatory authorities and EMD Serono, as required by local regulations (for regulatory reporting) and as required by the ISS agreement (for reporting to EMD Serono).

The following reportable events must be submitted to the Sponsor within 24 hours (or immediately for death or life-threatening events). [The Sponsor will assume responsibility for submitting the reportable event to EMD Serono as well as ensuring that any local reporting requirements are completed in parallel.

- **Serious Adverse Events**
- **Exposure during Pregnancy or Breastfeeding (even if not associated with an adverse event)**
- **Occupational exposure (even if not associated with an adverse event)**
- **Potential drug-induced liver injury (Hy's Law cases): These events are considered important medical events and should be reported as SAEs.**

Contact information for safety-related issues in case there are problems with the CRF platform:

VHIO
VHIO Safety e-mail address: crsu_fcv@vhio.net

Tlf: +34 932 543 450 ext. 8614

To enable VHIO to comply with regulatory reporting requirements, all initial SAE reports should always include the following minimal information: an identifiable patient (SeqID), a suspect medicinal product if applicable, an identifiable reporting source, the description of the medical event and seriousness criteria, as well as the causality assessment by the investigator. Complete information of any reported serious adverse event must be returned within 7 calendar days of the initial report. If all information is not received within this deadline, VHIO will make a written request to the investigator.

Queries sent out by the VHIO need to be answered within 7 calendar days.

8. STATISTICAL METHODS

8.1 Hypothesis

The primary hypothesis to be tested in this single-arm phase II clinical trial is whether the addition of avelumab to the FLOT schema improves the pCR compared to the historical pCR for chemotherapy alone in the neoadjuvant setting. p0 is the pCR rate of the historical data for patients who receive only chemotherapy (FLOT schema). In contrast, p1 is the estimated pCR rate for the study treatment.

The null hypothesis is that the true pCR rate (p) is equal or less than p_0, while the alternative hypothesis is that the true pCR rate is equal or greater than p1.

$$H_0: \ p \leq p_0 \qquad \text{vs.} \qquad H_1: p \geq p_1$$

8.2 Determination of Sample Size

To test the null hypothesis of $H_0: \ p \leq p_0$, the sample size is calculated according single-stage phase II design based on the exact binomial distribution. The pCR of the historical data is estimated at 16% (based on FLOT schema) and the pCR rate in the study treatment is estimated at 33%. The study will accrue up to 30 evaluable patients in order to reject the null hypothesis with 82% power using one-sided type I error of 0.1. The null hypothesis will be rejected if at least 8 out of 30 evaluable patients achieved a pCR. 37 patients will be recruited considering a 10% drop-out and a potential 10% of screening failures.

Power calculation for various pCR rates are shown in the next table:

True pCR rate	Probability to reject H_0
35%	88%
33%	82%
30%	72%
25%	49%

8.3 Feasibility of enrolling proposed population

The prognosis of resectable GC and GEJC patients is poor, and perioperative chemotherapy has been established as the standard of care, when feasible. Perioperative chemotherapy has shown to improve the survival of these patients, probably by increasing the pathological regression of the tumor. PD1/PDL1 checkpoints inhibitors have shown very interesting activity in terms of a high response rate, in the metastatic setting.

Taking into account the still poor outcome of these patients treated with the standard of care, the feasibility of the recruitment in this phase II study with avelumab would be easy and promising.

8.4 Statistical criteria of termination of trial

Data cut-off for the primary analysis will happen following after the last patient included in the study has performed the gastric surgery and pathology report is available. The subsequent tumour evaluations will take place every 6 months to define DFS, PFS and OS.

8.5 Planned Analyses

8.5.1 Definition of study populations for analysis

- The primary endpoint and DFS will be assessed in the intention to treat (ITT) population.
- The other endpoints will be assessed in the intention to treat (ITT) population.
- Safety population: All study patients who receive at least 1 dose of study treatment. The safety analysis will be based on an all patients treated.

8.5.2 General considerations

Summary tables (descriptive statistics and frequency tables) will be provided for all demographic variables, baseline variables, non-time to event efficacy variables (unless specified otherwise), and safety variables, as appropriate. Continuous variables will be summarized with descriptive statistics (mean, standard deviation, range, and median).

Ninety-five (95) percent confidence intervals (95% CI) may also be presented, as appropriate. Frequency counts and percentage of subjects within each category will be provided for categorical data. Additionally, for the pCR rate one-sided 90% CI will be also presented to be consistent with sample size calculation.

8.5.3 Analysis of Primary Endpoints

Pathological complete response (pCR) rate:

The primary clinical endpoint for this study is efficacy as assessed by pCR rate and will be analysed based on the percentage of patients who obtained a pathological complete response. As mentioned before, pathologic complete response (grade 1a) is defined as no evidence of residual tumour based on pathological review of the surgical specimen.

Number of patients with pCR and percentage will be provided. The proportion of patients achieving a complete pathological complete response rate will be compared with the historical pCR rate (estimated at 16%, based on FLOT schema) using the conditional binomial exact test. The two-sided 95% CI and one-sided 90%CI for the estimate pCR will be presented.

8.5.4 Analysis of Secondary Endpoints

- Overall survival (OS):

The overall survival (OS) is defined as the time (in months) from the initial date of the study first treatment cycle to the date of death, regardless of the actual cause of the subject's death. For subjects who are still alive at the time of data analysis or who are lost to follow-up, OS time will be censored at the last recorded date that the subject is known to be alive (date of last contact, last visit date, date of last trial treatment administration, or date of last scan, whichever is the latest) as of the data cut-off date for the analysis. If the date of last known status of alive or death date is after the data cut-off date, subjects will be censored at the data cut-off date.

OS will be analysed according to the Kaplan-Meier method. Kaplan-Meier survival curve will be reported, along with associated 95% CI. In addition, OS a for various time points (e.g. 3-year OS rate) will be presented together with 95% CIs. The analyses will be performed on an intention to treat basis.

- Disease-free survival (DFS):

Disease-free survival (DFS) is defined as the time (in months) from the surgery to the date of the first documented disease relapse or death from any cause. Relapse will be determined according to RECIST v1.1 as per investigator assessment.

Patients without an event prior to the study close-out date will be censored at the last relapse-free assessment. If a patient receives a subsequent anti-cancer therapy without prior documentation of disease progression, the patient will be censored at the date the patient was last seen for tumour assessment before starting the new chemotherapy. DFS will be evaluated using Kaplan-Meier methods and a Kaplan-Meier curve will be drawn. Estimates of the median DFS and for various time points (e.g. 3-year DFS rate) will be presented together with 95% CIs.

- Progression-free survival (PFS)

Progression-free survival (PFS) is defined as the time (in months) from the initial date of neoadjuvant chemotherapy to the date of first documentation of disease progression or death due to any cause, whichever occurs first. Progression is defined according to RECIST v1.1 assessed as per investigator assessment.

Patients without an event prior to the time of analysis will be censored at the last assessment that is stable disease (SD) or better. PFS will be evaluated using Kaplan-Meier methods and a Kaplan-Meier curve will be drawn. Estimates of the median PFS and for various time points (e.g. 3-year PFS rate) will be presented together with 95% CIs.

- Surgical complete resection rate (R0):

Number and percentage of patients achieving a complete resection rate in the ITT population,

assessed within the surgery and by the pathologist, as referred in section 7.3.2. Patients who did not undergo surgery will be included in the evaluation and considered as not having achieved R0 resection.

- Overall Response Rate (ORR):

Proportion of all subjects achieving complete response (CR) and partial response (PR) according to Response Evaluation Criteria in Solid tumors (RECIST) v1.1 and as adjudicated by the local investigator.

8.5.5 Analysis of Exploratory Endpoints

- Pathological immune response (pIR) (immunoscore)

The pathological inmune response will be evaluated in the surgical tissue, by central pathology review. The immunoscore described by Galon et al. [49] will be applied, taking into consideration the two lymphocyte populations (CD3/CD45RO, CD3/CD8 or CD8/CD45RO) both in the core of the tumor and the invasive margin. The Immunoscore provides a score ranging from Immunoscore 0 (I0) when low densities of both cell types are found in both regions, to Immunoscore 4 (I4) when high densities are found in both regions.

Population: Operated patients that had performed at least one cycle of avelumab/FLOT neoadjuvant treatment.

- Characterization of infiltrating immune contexture

Multiplexed fluorescence immunohistochemistry assays designed to detect expression of: *i)* key lymphocyte markers such as PD-1, CD137, LAG-3, CD4, CD8, and FOXP3; and *ii)* myeloid cell markers such as Arginase, CD68, CD11b, CD33, CD14, CD66b will be used to characterize the immune contexture. Fluorescence images will acquired on the Vectra Polaris platform (Perkin Elmer). Data analysis will be performed with inForm software (Perkin Elmer). The status of PD1 and PDL1 receptors in diagnostic tissue and in tissue from surgery will be also assessed. Furthermore, the microsatellite instability status and a nanostring immune panel will be run in the surgical tissue. Population: Operated patients that had performed at least one cycle of avelumab/FLOT neoadjuvant treatment.

- Immunodynamic follow-up

Immunodynamic follow-up will be assessed by analyzing the blood samples obtained at different timepoints of the study protocol (see Table 1). ELISA test will be performed for cytokine analyzes and TILs phenotype assessment. Population: Patients that had perform at least one cycle of avelumab/FLOT neoadjuvant treatment.

TIL expansion (with an stablisment of tumor lines) as well as a predefined immune nanostring pannel will be performed on the surgery tissue of 10 patients. Population: 10 operated patients that had performed the four cycles of avelumab/FLOT neoadjuvant treatment.

- TCR clonality assessment

TCR clonality assessment in the surgical tissue of the patients will be evaluated by the Adaptative test.

Population: Operated patients that had performed at least one cycle of avelumab/FLOT neoadjuvant treatment.

8.5.6 Analysis of Safety Endpoints

Safety analyses will be based on the Safety Analysis Set. Adverse events and serious adverse events, laboratory test results, physical examination findings and vital signs, and their changes from baseline will be summarized using descriptive statistics.

Toxicity profile will be described per patient to show all the AE information about grade, relationship to study treatment and severity. Toxicity will be evaluated according NCI-CTCAE v4.0 criteria.

9. ADMINISTRATIVE ASPECTS

9.1 Ethical conduct of the study

The current clinical trial will be conducted in accordance with the protocol, the principles established in the current revised version of the Declaration of Helsinki and the applicable regulatory requirements, particularly the ICH Tripartite Harmonized Guidelines for good clinical practice and the Royal Decree on Clinical Trials 1090/2015, by which clinical trials with medications are regulated in Spain, which fully incorporates the stipulations of European Clinical Trials Regulation (Regulation (EU) No. 536/2014) and any applicable Regulatory requirement relative to the dispositions of the Member States for the application of GCP (CPMP/ICH/135/95) to clinical trials of medications for human use.

The sponsor has contracted an insurance policy to cover the responsibilities of the investigator and other parties participating in the study, according to the applicable Spanish legislation.

The sponsor commits to responsible publication of both the positive and negative results from its clinical trials as required by all governing regulatory and health authorities.

9.1.1 Independent Ethics Committee

ICH guidelines require that approval be obtained from Health Authorities and an Ethics Committee before human subjects can participate in research studies. Prior to the trial onset, the clinical trial protocol, will be submitted together with its associated documents (such as the ICF) to the responsible IEC for its favourable opinion or approval.

The clinical trial protocol and any applicable documentation (e.g., Investigational Medicinal Product Dossier, Subject Information, and the ICF) will be also submitted or notified to the Health Authorities in accordance with all local and national regulations for each site. The clinical study will only be started when both the Health Authorities and an Ethics Committee have considered that the expected benefits for the trial subject and society justify the risks; in addition, the trial will only be continued if compliance with this criterion is constantly supervised.

All Ethics Committee approvals should be signed by the Ethics Committee Chairman or designee and must identify the Ethics Committee name and address, the clinical protocol by title and/or protocol number and the date approval and/or favourable opinion was granted. Documentation of all Health Authorities and Ethics Committee approvals and of the Ethics Committee compliance with ICH E6 will be maintained by the site and will be available for review.

Amendments to this clinical trial protocol will also be submitted to the concerned IEC, before implementation of substantial changes.

9.1.2 Patient Information and Informed consent

The investigators will explain to each participant the nature of the study, its purpose, the procedures involved, the expected duration, the potential risks and benefits and any discomfort it may entail. Each participant will be informed that the participation in the study is voluntary and that he/she may withdraw from the study at any time and that withdrawal of consent will not affect his/her subsequent medical assistance and treatment.

The participant must be informed that his/her medical records may be examined by authorised individuals other than their treating physician.

All participants for the study will be provided a participant information sheet and a consent form describing the study and providing sufficient information for participant to make an informed decision about their participation in the study.

The patient information sheet and the consent form will be submitted to the Ethics Committee to be reviewed and approved. The formal consent of a participant, using the approved consent form, must be obtained before the participant is submitted to any study procedure.

The participant should read and consider the statement before signing and dating the informed consent form, and should be given a copy of the signed document. The consent form must also be signed and dated by the investigator (or his designee) and it will be retained as part of the study records.

9.2 Confidentiality

Each patient is assigned a unique patient study number at enrolment. In trial documents the patient's identity is coded by patient study number as assigned at enrolment. The local investigator will keep a subject enrolment and identification log that contains the key to the code, i.e. a record of the personal identification data linked to each patient study number. This record is filed at the investigational site and should only be accessed by the investigator and the supporting site staff, and by representatives of the sponsor or a regulatory agency for the purpose of monitoring visits or audits and inspections. The Investigator and the Sponsor agree to adhere to the principles of personal data confidentiality in relation to the patients, Investigator, and its collaborators involved in the study.

9.3 Protocol amendments

Changes to the clinical trial protocol will be documented in written protocol amendments. Any substantial (major) amendments to the protocol must be submitted in writing to the Ethics Committee and the Health Authorities for approval before the changes proposed in the amendment are implemented. Depending on the magnitude of the change, the recruitment may be temporally halted. Protocol changes introduced to eliminate an impending and obvious risk may be implemented immediately, but must subsequently be documented in an amendment, reported to the Ethics

Committee and be submitted to the relevant Health Authorities within the required timeframe.

The sponsor does not have to notify non-substantial (minor) amendments to the Health Authorities or the Ethics Committee. However, any non-substantial amendments will be recorded and contained in the documentation when it is subsequently submitted, for example in the subsequent notification of a substantial amendment. Documentation of any non-substantial amendments will be available on request for inspection at the trial site or the sponsor premises as appropriate.

Any amendment that could affect the subject’s agreement to participate in the trial requires additional informed consent prior to implementation.

9.4 Storage of samples

Storage of biological samples on site is subject to the site’s guidelines; samples may be labelled with the patients identifying information (e.g. name, hospital record number). Samples that are shipped to another facility (e.g. a central laboratory) for a purpose as described in this protocol or for additional scientific research, should be stripped from any identifying information and labelled with a code (trial name or number and patient study number as assigned at enrolment).

9.5 Data Handling and Record Keeping

Trial data will be recorded on the (e-)CRFs provided. All required data entry fields will be completed. Data corrections will be done according to the instructions provided. The investigator will be asked to confirm the accuracy of completed CRFs by signing key CRFs as indicated.

Source documents pertaining to the trial must be maintained by investigational sites. Source documents may include a subject's medical records, hospital charts, clinic charts, the investigator's subject study files, as well as the results of diagnostic tests such as X-rays, CT scans, laboratory tests, and electrocardiograms. The investigator's copy of the case report forms serves as part of the investigator's record of a subject's study-related data.

All study-related documentation will be maintained for 15 years following completion of the study.

9.6 Study Monitoring

Data from this study will be monitored by CRS Unit. Monitoring will include centralised review of CRFs and other study documents for protocol compliance, data accuracy and completeness. Monitoring may include monitoring visits to investigational sites during for source data verification, review of the investigator’s site file and drug handling records. The monitor will be given access to source documents, CRFs and other study-related documents. By signing the informed consent form, the subject gives authorization to access to their medical records and the study data.

9.6.1 Responsibilities of the investigators

The Investigator(s) undertake(s) to perform the study in accordance with ICH Good Clinical Practice Guidelines.

The Investigator is required to ensure compliance with respect to the investigational drug schedule, visit schedule and procedures required by the protocol. The Investigator agrees to provide all information requested in the Case Report Form in an accurate manner according to the instructions provided.

9.7 Audit and Inspection

Authorised representatives of the sponsor, a regulatory authority, or an Ethics Committee may perform audits or inspections at the centre, including source data verification, so direct access to the source data and documents will be granted by the participating sites and investigators. The purpose of an audit or inspection is to systematically and independently examine all study related activities and documents, to determine whether these activities were conducted, and data were recorded, analysed, and accurately reported according to the protocol, Good Clinical Practice (GCP), guidelines of the International Conference on Harmonisation (ICH), and any applicable regulatory requirements. The investigator/ site staff should contact the site monitor immediately if contacted by a regulatory agency about an inspection at the centre.

9.8 Clinical Study Report

Following completion of the study, a clinical study report (CSR) compliant with the requirements of ICH E3 will be prepared. In compliance with the regulations, the final report will be produced within one year of completing the study.

9.9 Publication Policy

The first publication will be a publication of the results of the analysis of the primary endpoints that

will include data from all trial sites.

The Investigator will inform the Sponsor in advance about any plans to publish or present data from the trial. Any publications and presentations of the results (abstracts in journals or newspapers, oral presentations, etc.), either in whole or in part, by Investigators or their representatives will require presubmission review by the Sponsor.

10. REFERENCES

1. Becker K, Mueller JD, Schulmacher C, Ott K, Fink U, Busch R, Böttcher K, Siewert JR, Höfler H. Histomorphology and grading of regression in gastric carcinoma treated with neoadjuvant chemotherapy. Cancer. 2003;98(7):1521-301. Goode, E.F. and Smyth, E.C. Immunotherapy for gastroesophageal cancer. J Clin Med. 2016;5:E84.

2. Al-Batran SE, Hofheinz RD, Pauligk C, Kopp HG, Haag GM, Luley KB, et al. Histopathological regression after neoadjuvant docetaxel, oxaliplatin, fluorouracil, and leucovorin versus epirubicin, cisplatin, and fluorouracil or capecitabine in patients with resectable gastric or gastro-oesophageal junction adenocarcinoma (FLOT4-AIO): results from the phase 2 part of a multicentre, open-label, randomised phase 2/3 trial. Lancet Oncol. 2016;17(12):1697-1708.

3. Smyth EC, Verheij M, Allum W, Cunningham D, Cervantes A, Arnold D; ESMO Guidelines Committee. Gastric cancer: ESMO Clinical Practice Guidelines for diagnosis, treatment and follow-up. Ann Oncol. 2016;27(suppl 5):v38-v49.

4. Torre LA, Bray F, Siegel RL, Ferlay J, Lortet-Tieulent J, Jemal A. Global cancer statistics, 2012. CA Cancer J Clin. 2015;65(2):87-108.

5. Cunningham D, Allum WH, Stenning SP, Thompson JN, Van de Velde CJ, Nicolson M, et al. Perioperative chemotherapy versus surgery alone for resectable gastroesophageal cancer. N Engl J Med. 2006;355:11-20.

6. Ychou M, Boige V, Pignon JP, Conroy T, Bouché O, Lebreton G, et al. Perioperative chemotherapy compared with surgery alone for resectable gastroesophageal adenocarcinoma: an FNCLCC and FFCD multicenter phase III trial. J Clin Oncol. 2011;29:1715-1721.

7. Network CGAR. Comprehensive molecular characterization of gastric adenocarcinoma. Nature. 2014;513: 202-209.

8. Cristescu R, Lee J, Nebozhyn M, Kim KM, Ting JC, Wong SS, et al. Molecular analysis of gastric cancer identifies subtypes associated with distinct clinical outcomes. Nat Med. 2015;21:449-456.

9. Lei Z, Tan IB, Das K et al. Identification of molecular subtypes of gastric cancer with different responses to PI3-kinase inhibitors and 5-fluorouracil. Gastroenterology. 2013;145:554-565.

10. Maron SB LJ, Hovey R, Bao R, Gajewski TF, Ji Y, Seiwert TY, et al. Molecular Characterization of T-Cell-Inflamed Gastroesophageal Carcinoma in T-cell-inflamed gastric carcinoma. In Society for Immunotherapy of Cancer (SITC). 2017.

11. Muro K, Bang Y, Shankaran V et al. LBA15A phase 1B study of pembrolizumab (PEMBRO; MK- 3475) in patients (Pts) with advanced gastric cancer. Annals of Oncology 2014; 25: mdu438. 415.

12. Le DT, Bendell JC, Calvo E et al. Safety and activity of nivolumab monotherapy in advanced and metastatic (A/M) gastric or gastroesophageal junction cancer (GC/GEC): Results from the CheckMate-032 study. J Clin Oncol 2016; 34: 6.

13. Segal N, Hamid O, Hwu W et al. 1058PDA phase I multi-arm dose-expansion study of the anti-programmed cell death-ligand-1 (Pd-L1) antibody Medi4736: preliminary data. Annals of Oncology 2014; 25: iv365-iv365.

14. Herbst RS, Gordon MS, Fine GD et al. A study of MPDL3280A, an engineered PD-L1 antibody in patients with locally advanced or metastatic tumors. In ASCO Annual Meeting Proceedings. 2013; 3000.

15. Chung HC, Arkenau HT, Lee J, Rha SY, Oh DY, Wyrwicz L, et al. Avelumab (anti-PD-L1) as first- line maintenance (1L mn) or second-line (2L) therapy in patients with advanced gastric or gastroesophageal junction cancer (GC/GEJC): updated phase Ib results from the JAVELIN Solid Tumor trial [abstract]. In: Proceedings of the American Association for Cancer Research Annual Meeting 2018; 2018 Apr 14-18; Chicago, IL. Philadelphia (PA): AACR; Cancer Res 2018;78(13 Suppl):Abstract nr CT111.

16. Kang YK, Boku N, Satoh T, Ryu MH, Chao Y, Kato K, et al. Nivolumab in patients with advanced gastric or gastro-oesophageal junction cancer refractory to, or intolerant of, at least two previous chemotherapy regimens (ONO-4538-12, ATTRACTION-2): a randomised, double-blind, placebo-controlled, phase 3 trial. Lancet. 2017 Dec 2;390(10111):2461-2471.

17. Shitara K, Özgüro❑lu M, Bang YJ, Di Bartolomeo M, Mandalà M, Ryu MH, et al. Pembrolizumab versus paclitaxel for previously treated, advanced gastric or gastro-oesophageal junction cancer (KEYNOTE-061): a randomised, open-label, controlled, phase 3 trial. Lancet. 2018 Jul 14;392(10142):123-133.

18. Bang YJ, Ruiz EY, Van Cutsem E, Lee KW, Wyrwicz L, Schenker M, et al. Phase 3, randomised trial of avelumab versus physician's choice of chemotherapy as third-line treatment for patients with advanced gastric or gastro-oesophageal junction cancer: primary analysis of JAVELIN Gastric 300. Ann Oncol. 2018 Jul 24.

19. Muro K BY, Shankaran V, et al. Relationship between PD-L1 expression and clinical outcomes in patients (Pts) with advanced gastric cancer treated with the anti-PD-1 monoclonal antibody pembrolizumab (Pembro; MK-3475) in KEYNOTE-012. J Clin Oncol 2015; 33 (Suppl 3, abstr 3).

20. Ribas A, Robert C, Hodi FS et al. Association of response to programmed death receptor 1 (PD-1) blockade with pembrolizumab (MK-3475) with an interferon-inflammatory immune gene signature. In ASCO Annual Meeting Proceedings. 2015; 3001.

21. Bang Y, Chung H, Shankaran V et al. LBA-04Clinical outcomes and their correlation with gene

expression in patients with advanced gastric cancer treated with pembrolizumab (MK-3475): KEYNOTE-012. Annals of Oncology 2015; 26: iv118-iv118.

22. Keir ME, Butte MJ, Freeman GJ, Sharpe AH. PD-1 and its ligands in tolerance and immunity. Annu Rev Immunol. 2008;26:677-704.

23. Butte MJ, Keir ME, Phamduy TB, Freeman GJ, Sharpe AH. PD-L1 interacts specifically with B7-1 to inhibit T cell proliferation. Immunity. 2007;27:111-22.

24. Zou W, Chen L. Inhibitory B7-family molecules in the tumour microenvironment. Nat Rev Immunol. 2008;8(6):467-77.

25. Paterson AM, Brown KE, Keir ME, Vanguri VK, Riella LV, Chandraker A, et al. The PD L1:B7-1 pathway restrains diabetogenic effector T cells in vivo. J Immunol. 2011;187:1097 105.

26. Brahmer JR, Tykodi SS, Chow LQM, Hwu WJ, Topalian SL, Hwu P, et al. Safety and activity of anti-PD-L1 antibody in patients with advanced cancer. N Engl J Med. 2012 Jun;366(26):2455-65

27. Hirano F, Kaneko K, Tamura H, Dong H, Wang S, Ichikawa M, et al. Blockade of B7-H1 and PD-1 by monoclonal antibodies potentiates cancer therapeutic immunity. Cancer Res. 2005;65(3):1089-96.

28. Okudaira K, Hokari R, Tsuzuki Y, Okada Y, Komoto S, Watanabe C, et al. Blockade of B7-H1 or B7-DC induces an anti-tumor effect in a mouse pancreatic cancer model. Int J Oncol. 2009 Sep;35(4):741-9.

29. Topalian SL, Hodi FS, Brahmer JR, Gettinger SN, Smith DC, McDermott DF, et al. Safety, activity, and immune correlates of anti-PD-1 antibody in cancer. N Engl J Med. 2012;366:2443-54.

30. Zhang C, Wu S, Xue X, Li M, Qin X, Li W, et al. Anti-tumor immunotherapy by blockade of the PD-1/PD-L1 pathway with recombinant human PD-1-IgV. Cytotherapy. 2008;10(7):711-9.

31. Powles T, Eder JP, Fine GD, Braiteh FS, Loriot Y, Cruz C, et al. MPDL3280A (anti-PD-L1) treatment leads to clinical activity in metastatic bladder cancer. Nature. 2014 Nov 27;515(7528):558- 62.

32. Rizvi N, Brahmer J, Ou S-H, Segal NH, Khleif SN, Hwu WJ. Safety and clinical activity of MEDI4736, an anti-programmed cell death-ligand-1 (PD-L1) antibody, in patients with nonsmall cell lung cancer (NSCLC). J Clin Oncol 2015;33:Abstract 8032.

33. Segal NH, Ou S-HI, Balmanoukian AS, Fury MG, Massarelli E, Brahmer JR, et al. Safety and efficacy of MEDI4736, an anti-PD-L1 antibody, in patients from a squamous cell carcinoma of the head and neck (SCCHN) expansion cohort. J Clin Oncol 2015;33:Abstract 3011.

34. Chivu-Economescu M, Matei L, Necula LG, Dragu DL, Bleotu C, Diaconu CC. New therapeutic options opened by the molecular classification of gastric cancer. World J Gastroenterol. 2018 May 14;24(18):1942-1961.

35. Latchman Y, Wood CR, Chernova T, et al. PD-L2 is a second ligand for PD-1 and inhibits T cell activation. Nature Immunol. 2001;2(3):261-8.

36. Kaufman H, Russell JS, Hamid O, Bhatia S, Terheyden P, D'Angelo SP, Shih KC, Lebbe C, Linette GP, Milella M, et al. Avelumab (MSB0010718C; anti-PD-L1) in patients with metastatic Merkel cell carcinoma previously treated with chemotherapy: Results of the phase 2 JAVELIN Merkel 200 trial. J Clin Oncol. 2016;34:9508–9508.

37. Oki E, Okano S, Ando K, et al. HER2 and programmed death-1 ligand-1 (PD-L1) expression in gastric carcinoma. J Clin Oncol. 2014;32(No 15_suppl (May 20 Supplement)):e15041.

38. Doi T, Iwasa S, Muro K, Satoh S, Hironaka T, Esaki T, et al. Phase 1 trial of avelumab (anti-PD- L1) in Japanese patients with advanced solid tumors, including dose expansion in patients with gastric or gastroesophageal junction cancer: the JAVELIN Solid Tumor JPN trial. Gastric Cancer. 2018: 1-11.

39. Markus H. Moehler, Julien Taïeb, Jayne S. Gurtler, Huiling Xiong, Jenny Zhang, Jean-Marie Cuillerot, and Narikazu Boku. Maintenance therapy with avelumab (MSB0010718C; anti-PD-L1) vs continuation of first-line chemotherapy in patients with unresectable, locally advanced or metastatic gastric cancer: The phase 3 JAVELIN Gastric 100 trial. Journal of Clinical Oncology 2016 34:15_suppl, TPS4134-TPS4134.

40. Mansukhani S, Davidson M, Gillbanks A, Peckitt C, Musallam A, Begum R, et al. Iconic: Peri-operative immuno-chemotherapy in operable oesophageal and gastric cancer. Journal of Clinical Oncology 2018 36:15_suppl, TPS4139-TPS4139.

41. Al-Batran SE, Homann N, Schmalenberg H, Kopp HG, Haag GM, et al. Perioperative chemotherapy with docetaxel, oxaliplatin, and fluorouracil/leucovorin (FLOT) versus epirubicin, cisplatin, and fluorouracil or capecitabine (ECF/ECX) for resectable gastric or gastroesophageal junction (GEJ) adenocarcinoma (FLOT4-AIO): A multicenter, randomized phase 3 trial. Journal of Clinical Oncology 2017 35:15_suppl, 4004-4004.

42. Pardoll DM. The blockade of immune checkpoints in cancer immunotherapy. Nature Reviews Cancer 2012; 12: 252-264.

43. Eggermont AM, Chiarion-Sileni V, Grob JJ, Dummer R, Wolchok JD, Schmidt H. Adjuvant ipilimumab versus placebo after complete resection of high-risk stage III melanoma (EORTC 18071): a randomised, double-blind, phase 3 trial. Oncol 2015;16: 522–30.

44. Kelly K, Heery CR, Patel MR. et al. Avelumab (MSB0010718C; anti-PD-L1) in patients with advanced cancer: Safety data from 1300 patients enrolled in the phase 1b JAVELIN Solid Tumor trial. J Clin Oncol2016; 34(Suppl 15): Abstract 3055

45. Weber JS, Yang JC, Atkins MB, Disis ML. Toxicities of immunotherapy for the practitioner. J Clin

Oncol. 2015; 33(18): 2092–2099.

46. Spain L, Diem S, Larkin J. Management of toxicities of immune checkpoint inhibitors. Cancer Treat Rev 2016; 44: 51–60.

47. Haanen JBAG, Carbonnel F, Robert C, Kerr KM, Peters S, Larkin J, et al. Management of toxicities from immunotherapy: ESMO Clinical Practice Guidelines for diagnosis, treatment and follow- up. Ann Oncol 2017;28. iv119-42.

48. Postow MA. Managing immune checkpoint-blocking antibody side effects. Am Soc Clin Oncol Educ Book 2015:76–83.

49. Galon J, Mlecnik B, Bindea G, Angell HK, Berger A, Lagorce C, et al. Towards the introduction of the 'Immunoscore' in the classification of malignant tumours. J Pathol. 2014;232(2):199-209.

50. Kohrt HE, Tumeh PC, Benson D, Bhardwaj N, Brody J, Formenti S, et al. Immunodynamics: a cancer immunotherapy trials network review of immune monitoring in immuno-oncology clinical trials. J Immunother Cancer. 2016;4:15.

51. Mahe E, Pugh T, Kamel-Reid S. T cell clonality assessment: past, present and future. J Clin Pathol. 2018;71(3):195-200.

52. Howard SC, Jones DP, Pui CH. The tumor lysis syndrome. N Engl J Med. 2011;364(19):1844-54.

11. APPENDICES

11.1 Recommendation for dose modifications and toxicity management for FLOT chemotherapy

11.1.1 Hematotoxicity

On day 1 of each cycle, FLOT chemotherapy can be administered if:

- leukocytes are at ≥ 3.0 x 109/l1 and
- thrombocytes are at ≥ 100 x 109/l and
- relevant non-hematologic toxicity is < grade 2 and
- in the absence of fever or a relevant infection

If above mentioned criteria are not met on day 1, patients should receive the supportive care required according to local protocols until all requirements for treatment continuation are met.

Notes:
1. For FLOT therapy, the overall number of leukocytes is relevant, not the neutrophils. Treatment can be continued if leukocytes are at ≥ 3.0 x 109/l or higher, independently of the number of neutrophils – provided the patient has no infection, fever or other side effects that are possibly neutropenia-related.

2. Granulocyte-colony stimulating factor [G-CSF] may be used as secondary prophylaxis for patients who had febrile neutropaenia or treatment interruptions because of neutropaenia or leukopaenia, or per local guidelines practise [2].

Patients who experience febrile neutropenia (despite the use of G-CSF), or thrombocytopenia causing bleeding, or any other hematological dose limiting toxicities (DLT, investigator decision) will receive a dose reduction of docetaxel and oxaliplatin to 75% of the initial dose. Subsequent dose limiting toxicities lead to a further dose reduction to 50% of the initial dose level. If DLTs reoccur at the 50% dose level, the investigator may remove one or both of the drugs (investigator decision).

11.1.2 Oxaliplatin neurotoxicity

Dose adaptations for peripheral neurotoxicity are provided in Table 9.

Table 9. Oxaliplatin dose modification in case of neurotoxicity

Neurotoxicity	≤ 7 days	>7 and < 14 days	Present between cycles
Cold-induced dysaesthesia	no change	no change	no change
Paraesthesia	no change	no change	reduction to 75%

Paraesthesia with pain	no change	reduction to 75%	stop oxaliplatin* continue docetaxel/5-FU/LV
Paraesthesia with functional impairment	no change	reduction to 50%	stop oxaliplatin* continue docetaxel/5-FU/LV

* Usually, discontinuation will be permanently. Nevertheless, administration of oxaliplatin can be resumed (e.g. after complete recovery from the related symptoms) if the investigator decides that this is in the best interest of the patient and if he does not expect the toxicity to reoccur. Abbreviation: LV, leucovorin.

11.1.3 Oxaliplatin renal toxicity

Creatinine clearance should be calculated or measured at baseline and prior to each cycle of chemotherapy. Calculations can be made according to local practice.. If creatinine clearance is less 50mls/min then the dose reduction of oxaliplatin should be according to table 10 below.

Table 10. Oxaliplatin dose modification in case of nephrotoxicity

**Creatinine clearance**	**Oxaliplatin dose**
≥ 50 mls /min	100%
30-49.9ml/min	75%
< 30 mls/min	Omit oxaliplatin

11.1.4 Docetaxel liver toxicity

Dose adaptations for docetaxel liver toxicity are provided in Table 11.

Table 11. Docetaxel dose modification in case of related liver toxicity

**Liver function tests**		**Docetaxel dose**
**Bilirubin**	**Hepatic aminotransferases (ALT, AST)**	
> ULN - 1.5 x ULN		Reduce dose by 25%
> 1.5 x ULN		Reduce dose by 50%
> 3 x ULN	> 2.5 x ULN	Omit docetaxel

11.1.5 Other toxicities for FLOT

If non-hematologic toxicities ≥ grade 3 appear, the dose of the chemotherapeutic agent most likely responsible for the observed toxicity should be reduced to 75% of the initial dose (for all further cycles). This procedure may apply to grade 2 toxicities upon investigator's decision.

In case of repeated toxicity, a further dose reduction to 50% of the initial dose should be performed. If the toxicity re-occurs at the 50% dose level, the investigator should remove the relevant drug(s) or stop the whole treatment.

11.2 Dose modifications and toxicity management for avelumab

11.2.1 Infusion-related Reactions

Symptoms of infusion-related reactions are fever, chills, rigors, diaphoresis, and headache. Treatment modification for symptoms of infusion-related Reactions are provided in Table 12.

Table 12. Treatment Modification for Symptoms of Infusion-related Reactions Associated with avelumab

**NCI-CTCAE Grade v4.03**	**Treatment Modification for Avelumab**
**Grade 1 – mild**<br>• Mild transient reaction; infusion interruption not indicated; intervention not indicated.	• Decrease the avelumab infusion rate by 50% and monitor closely for any worsening.<br>• The total infusion time for avelumab should not exceed 120 minutes.
**Grade 2 – moderate**<br>• Therapy or infusion interruption indicated but responds promptly to symptomatic treatment (for example, antihistamines, NSAIDs, narcotics, IV fluids); prophylactic medications indicated for ≤ 24 hours.	• Stop avelumab infusion.<br>• Resume infusion at 50% of previous rate once infusion-related reaction has resolved or decreased to at least Grade 1 in severity, and monitor closely for any worsening.

**Grade 3 or Grade 4 – severe or life-threatening**<br>• Grade 3: Prolonged (for example, not rapidly responsive to symptomatic medication and/or brief interruption of infusion); recurrence of symptoms following initial improvement; hospitalization indicated for clinical sequelae.	• Stop the avelumab infusion immediately and disconnect infusion tubing from the subject.<br>• Subjects have to be withdrawn immediately from avelumab treatment and must not receive any further avelumab treatment.
**NCI-CTCAE Grade v4.03**	**Treatment Modification for Avelumab**
• Grade 4: Life-threatening consequences; urgent intervention indicated.	

IV=intravenous, NCI-CTCAE=National Cancer Institute-Common Terminology Criteria for Adverse Event, NSAIDs=nonsteroidal anti-inflammatory drugs

- If avelumab infusion rate has been decreased by 50% or interrupted due to an infusion reaction, it must remain decreased for the next scheduled infusion. If no infusion reaction is observed in the next scheduled infusion, the infusion rate may be returned to baseline at the subsequent infusions based on investigator’s medical judgment.

11.2.2 Severe Hypersensitivity Reactions and Flue-like Symptoms

If hypersensitivity reaction occurs, the subject must be treated according to the best available medical practice. A complete guideline for the emergency treatment of anaphylactic reactions according to the Working Group of the Resuscitation Council (United Kingdom) can be found at https://www.resus.org.uk/pages/reaction.pdf. Subjects should be instructed to report any delayed reactions to the Investigator immediately.

Symptoms include impaired airway, decreased oxygen saturation (< 92%), confusion, lethargy, hypotension, pale or clammy skin, and cyanosis. These symptoms can be managed with epinephrine injection and dexamethasone. Subjects should be placed on monitor immediately, and the intensive care unit should be alerted for possible transfer if required.

For prophylaxis of flu-like symptoms, 25 mg indomethacin or comparable nonsteroidal anti-inflammatory drug dose (e.g., 600 mg ibuprofen, 500 mg naproxen sodium) may be administered 2 hours before and 8 hours after the start of each dose of avelumab IV infusion. Alternative

treatments for fever (for example, paracetamol/acetaminophen) may be given to subjects at the discretion of the Investigator.

11.2.3 Tumor Lysis Syndrome

In addition, because avelumab can induce antibody-dependent cell-mediated cytotoxicity (ADCC), there is a potential risk of tumor lysis syndrome. Should this occur, subjects should be treated per the local guidelines and the management algorithm (Figure 4) published by Howard et al (2011) [52].

Figure 4. Assessment and Initial Management of Tumor Lysis Syndrome

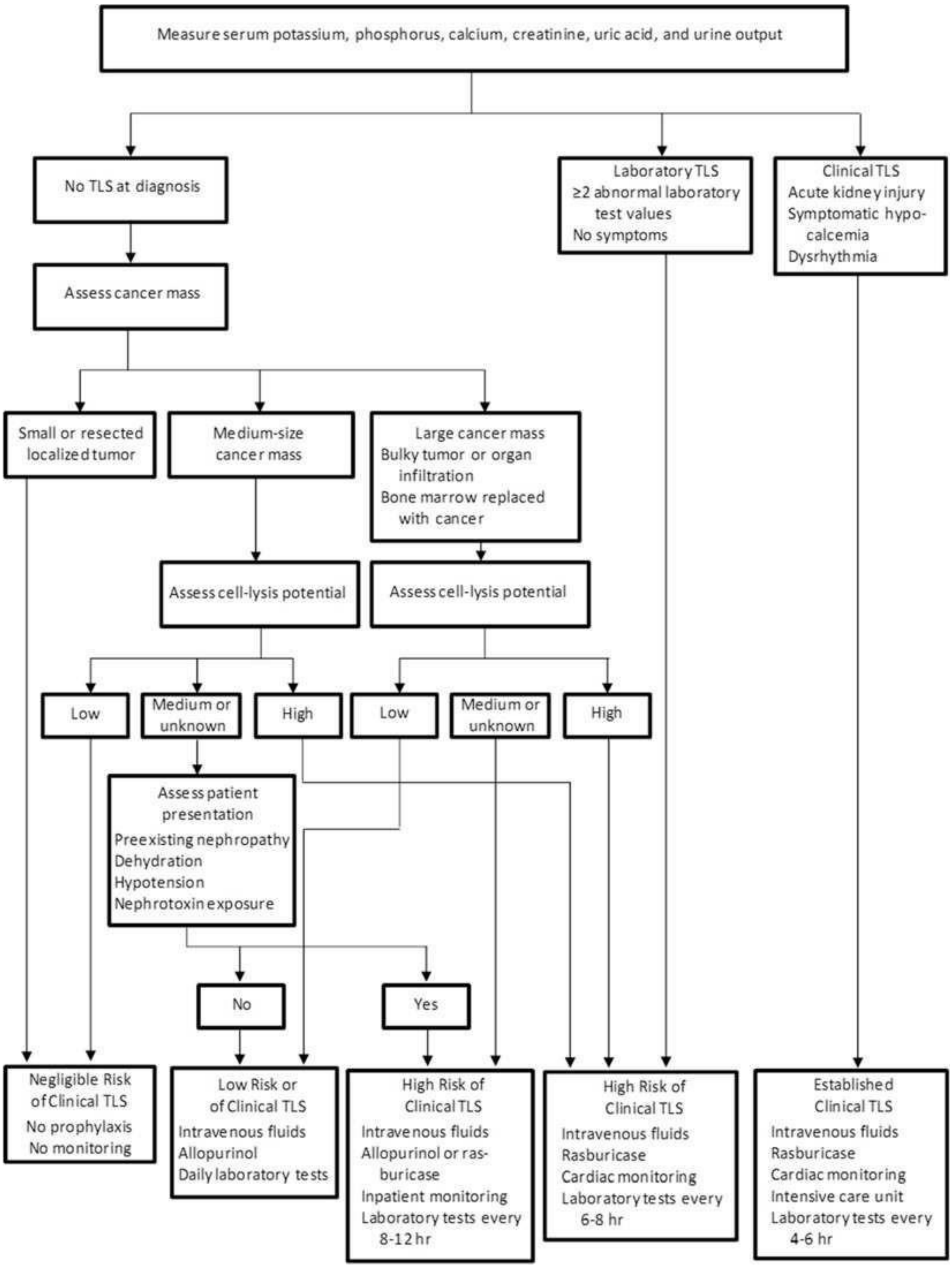

11.2.4 Immune-related Adverse Events

Because inhibition of PD-L1 stimulates the immune system, irAEs may occur. Treatment of irAEs is mainly dependent upon severity (NCI-CTCAE grade):

- Grades 1 to 2: treat symptomatically or with moderate dose steroids, more frequent monitoring
- Grades 1 to 2 (persistent): manage similar to high grade AE (Grades 3 to 4)
- Grades 3 to 4: treat with high dose corticosteroids. Treatment of irAEs should follow the guidelines in Table 13.

Table 13. Management of Immune-related Adverse Events

Note: for avelumab-related adverse events, only avelumab should be withheld (FLOT should follow usual schedule).

Gastrointestinal irAEs		
**Severity of Diarrhea/Colitis (NCI-CTCAE v4.0)**	**Initial Management**	**Follow-up Management**
**Grade 1**<br>Diarrhea: < 4 stools/day over Baseline<br>Colitis: asymptomatic	Continue avelumab therapy<br>Symptomatic treatment (e.g. loperamide)	Close monitoring for worsening symptoms<br>Educate subject to report worsening immediately<br>If worsens:<br>Treat as Grade 2, 3 or 4.
**Grade 2**<br>Diarrhea: 4 to 6 stools per day over Baseline; IV fluids indicated < 24 hours; not interfering with ADL<br>Colitis: abdominal pain; blood in stool	Withhold avelumab therapy<br>Symptomatic treatment	If improves to Grade ≤ 1:<br>Resume avelumab therapy<br><br>If persists > 5-7 days or recurs:<br>Treat as Grade 3 or 4.
**Grade 3 to 4**<br>Diarrhea (Grade 3): ≥ 7 stools per day over Baseline; incontinence; IV fluids ≥ 24 h; interfering with ADL<br>Colitis (Grade 3): severe abdominal pain, medical intervention indicated, peritoneal signs<br>Grade 4: life-threatening, perforation	Withhold avelumab for Grade 3.<br>Permanently discontinue avelumab for Grade 4 or recurrent Grade 3.<br><br>1.0 to 2.0 mg/kg/day prednisone IV or equivalent<br>Add prophylactic antibiotics for opportunistic infections<br>Consider lower endoscopy	If improves:<br>Continue steroids until Grade ≤ 1, then taper over at least 1 month; resume avelumab therapy following steroids taper (for initial Grade 3).<br><br>If worsens, persists > 3 to 5 days, or recurs after improvement:<br>Add infliximab 5mg/kg (if no contraindication). Note: infliximab should not be used in cases of perforation or sepsis.
**Dermatological irAEs**		
**Grade of Rash (NCI-CTCAE v4.0)**	**Initial Management**	**Follow-up Management**
**Grade 1 to 2**<br>Covering ≤ 30% body surface area	Continue avelumab therapy<br>Symptomatic therapy (for example, antihistamines, topical steroids)	If persists > 1 to 2 weeks or recurs:<br>Withhold avelumab therapy<br>Consider skin biopsy<br><br>Consider 0.5-1.0 mg/kg/day prednisone or equivalent. Once improving, taper steroids over at least 1 month, consider prophylactic antibiotics for opportunistic infections, and resume avelumab therapy following steroids taper.<br>If worsens:<br>Treat as Grade 3 to 4.

**Grade 3 to 4**<br>Grade 3: Covering > 30% body surface area;<br>Grade 4: Life threatening consequences	Withhold avelumab for Grade 3.<br>Permanently discontinue for Grade 4 or recurrent Grade 3.<br>Consider skin biopsy<br>Dermatology consult<br>1.0 to 2.0 mg/kg/day prednisone or equivalent<br>Add prophylactic antibiotics for opportunistic infections	If improves to Grade ≤ 1:<br>Taper steroids over at least 1 month; resume avelumab therapy following steroids taper (for initial Grade 3).
**Pulmonary irAEs**		
**Grade of Pneumonitis (NCI-CTCAE v4.0)**	**Initial Management**	**Follow-up Management**
**Grade 1**<br>Radiographic changes only	Consider withholding avelumab therapy<br>Monitor for symptoms every 2 to 3 days<br>Consider Pulmonary and Infectious Disease consults	Re-assess at least every 3 weeks<br>If worsens:<br>Treat as Grade 2 or Grade 3 to 4.
**Grade 2**<br>Mild to moderate new symptoms	Delay avelumab therapy<br>Pulmonary and Infectious Disease consults<br>Monitor symptoms daily; consider hospitalization<br>1.0 to 2.0 mg/kg/day prednisone or equivalent<br>Add prophylactic antibiotics for opportunistic infections<br>Consider bronchoscopy, lung	Re-assess every 1 to 3 days<br>If improves:<br>When symptoms return to Grade ≤ 1, taper steroids over at least 1 month, and then resume avelumab therapy following steroids taper<br>If not improving after 2 weeks or worsening:<br>Treat as Grade 3 to 4.
	biopsy	
**Grade 3 to 4**<br>Grade 3: Severe new symptoms; New/worsening hypoxia;<br>Grade 4: Life-threatening	Permanently discontinue avelumab therapy.<br>Hospitalize.<br>Pulmonary and Infectious Disease consults.<br>1.0 to 2.0 mg/kg/day prednisone or equivalent<br>Add prophylactic antibiotics for opportunistic infections<br>Consider bronchoscopy, lung biopsy	If improves to Grade ≤ 1:<br>Taper steroids over at least 1 month<br>If not improving after 48 hours or worsening:<br>Add additional immunosuppression (for example, infliximab, cyclophosphamide, IV immunoglobulin, or mycophenolate mofetil)
**Hepatic irAEs**		
**Grade of Liver Test Elevation (NCI-CTCAE v4.0)**	**Initial Management**	**Follow-up Management**

**Grade 1**<br>Grade 1 AST or ALT > ULN to 3.0 x ULN and/or Total bilirubin > ULN to 1.5 x ULN	Continue avelumab therapy	Continue liver function monitoring<br>If worsens:<br>Treat as Grade 2 or 3 to 4.
**Grade 2**<br>AST or ALT > 3.0 to ≤ 5 x ULN and/or total bilirubin > 1.5 to ≤ 3 x ULN	Delay avelumab therapy<br>Increase frequency of monitoring to every 3 days.	If returns to Grade ≤ 1:<br>Resume routine monitoring; resume avelumab therapy.<br>If elevation persists > 5 to 7 days or worsens:<br>Treat as Grade 3 to 4.
**Grade 3 to 4**<br>AST or ALT > 5 x ULN and/or total bilirubin > 3 x ULN	Permanently discontinue avelumab therapy<br>Increase frequency of monitoring to every 1 to 2 days<br>1.0 to 2.0 mg/kg/day prednisone or equivalent<br>Add prophylactic antibiotics for opportunistic infections<br>Consult gastroenterologist/ hepatologist<br>Consider obtaining MRI/CT scan of liver and liver biopsy if clinically warranted	If returns to Grade ≤ 1:<br>Taper steroids over at least 1 month<br>If does not improve in > 3 to 5 days, worsens or rebounds:<br>Add mycophenolate mofetil 1 gram (g) twice daily<br>If no response within an additional 3 to 5 days, consider other immunosuppressants per local guidelines.
**Renal irAEs**		
**Grade of Creatinine Increased (NCI-CTCAE v4.0)**	**Initial Management**	**Follow-up Management**
**Grade 1**<br>Creatinine increased > ULN to 1.5 x ULN	Continue avelumab therapy	Continue renal function monitoring<br>If worsens:<br>Treat as Grade 2 to 3 or 4.
**Grade 2 to 3**<br>Creatinine increased > 1.5 and ≤ 6 x ULN	Withhold avelumab therapy<br>Increase frequency of monitoring to every 3 days<br>1.0 to 2.0 mg/kg/day prednisone or equivalent.<br>Add prophylactic antibiotics for opportunistic infections<br>Consider renal biopsy	If returns to Grade ≤1:<br>Taper steroids over at least 1 month, and resume avelumab therapy following steroids taper.<br>If worsens:<br>Treat as Grade 4.
**Grade 4**<br>Creatinine increased > 6 x ULN	Permanently discontinue avelumab therapy<br>Monitor creatinine daily<br>1.0 to 2.0 mg/kg/day prednisone or equivalent.<br>Add prophylactic antibiotics for opportunistic infections<br>Consider renal biopsy<br>Nephrology consult	If returns to Grade ≤1:<br>Taper steroids over at least 1 month.
**Cardiac irAEs**		

Myocarditis	Initial Management	Follow-up Management
New onset of cardiac signs or symptoms and / or new laboratory cardiac biomarker elevations (e.g. troponin, CK-MB, BNP) or cardiac imaging abnormalities suggestive of myocarditis.	Withhold avelumab therapy.<br>Hospitalize.<br>In the presence of life threatening cardiac decompensation, consider transfer to a facility experienced in advanced heart failure and arrhythmia management.<br>Cardiology consult to establish etiology and rule-out immune-mediated myocarditis.<br>Guideline based supportive treatment as per cardiology consult.*<br><br>Consider myocardial biopsy if recommended per cardiology consult.	If symptoms improve and immune-mediated etiology is ruled out, re-start avelumab therapy.<br><br>If symptoms do not improve/worsen, viral myocarditis is excluded, and immune-mediated etiology is suspected or confirmed following cardiology consult, manage as immune-mediated myocarditis.
Immune-mediated myocarditis	Permanently discontinue avelumab.<br>Guideline based supportive treatment as appropriate as per cardiology consult.*<br>1.0 to 2.0 mg/kg/day prednisone or equivalent<br>Add prophylactic antibiotics for opportunistic infections.	Once improving, taper steroids over at least 1 month.<br><br>If no improvement or worsening, consider additional immunosuppressants (e.g. azathioprine, cyclosporine A).
*Local guidelines, or eg. ESC or AHA guidelines<br>ESC guidelines website: https://www.escardio.org/Guidelines/Clinical-Practice-Guidelines<br>AHA guidelines website:<br>http://professional.heart.org/professional/GuidelinesStatements/searchresults.jsp?q=&y=&t=1001		
**Endocrine irAEs**		
**Endocrine Disorder**	**Initial Management**	**Follow-up Management**
**Grade 1 or Grade 2 endocrinopathies (hypothyroidism, hyperthyroidism, adrenal insufficiency, type I diabetes mellitus)**	Continue avelumab therapy<br>Endocrinology consult if needed<br><br>Start thyroid hormone replacement therapy (for hypothyroidism), anti-thyroid treatment (for hyperthyroidism), corticosteroids (for adrenal insufficiency) or insulin (for Type I diabetes mellitus) as appropriate.<br><br>Rule-out secondary endocrinopathies (i.e. hypopituitarism / hypophysitis)	Continue hormone replacement/suppression and monitoring of endocrine function as appropriate.

**Grade 3 or Grade 4 endocrinopathies (hypothyroidism, hyperthyroidism, adrenal insufficiency, type I diabetes mellitus)**	Withhold avelumab therapy<br>Consider hospitalization<br>Endocrinology consult<br><br>Start thyroid hormone replacement therapy (for hypothyroidism), anti-thyroid treatment (for hyperthyroidism), corticosteroids (for adrenal insufficiency) or insulin (for type I diabetes mellitus) as appropriate.<br><br>Rule-out secondary endocrinopathies (i.e. hypopituitarism / hypophysitis)	Resume avelumab once symptoms and/or laboratory tests improve to Grade ≤ 1 (with or without hormone replacement/suppression).<br><br>Continue hormone replacement/suppression and monitoring of endocrine function as appropriate.
**Hypopituitarism/Hypophysitis (secondary endocrinopathies)**	If secondary thyroid and/or adrenal insufficiency is confirmed (i.e. subnormal serum FT4 with inappropriately low TSH and/or low serum cortisol with inappropriately low ACTH) :<br>• Refer to endocrinologist for dynamic testing as indicated and measurement of other hormones (FSH, LH, GH/IGF-1, PRL, testosterone in men, estrogens in women)<br>• Hormone replacement/suppressive therapy as appropriate<br>• Perform pituitary MRI and visual field examination as indicated	Resume avelumab once symptoms and hormone tests improve to Grade ≤ 1 (with or without hormone replacement).<br><br>In addition, for hypophysitis with abnormal MRI, resume avelumab only once shrinkage of the pituitary gland on MRI/CT scan is documented.<br><br>Continue hormone replacement/suppression therapy as appropriate.
	**If hypophysitis confirmed:**<br>• Continue avelumab if mild symptoms with normal MRI. Repeat the MRI in 1 month<br>• Withhold avelumab if moderate, severe or life-threatening symptoms of hypophysitis and/or abnormal MRI. Consider hospitalization. Initiate corticosteroids (1 to 2 mg/kg/day prednisone or equivalent) followed by corticosteroids taper during at least 1 month.<br>• Add prophylactic antibiotics for opportunistic infections.	
**Other irAEs (not described above)**		
**Grade of other irAEs (NCI-CTCAE v4.0)**	**Initial Management**	**Follow-up Management**

**Grade 2 or Grade 3 clinical signs or symptoms suggestive of a potential irAE**	Withhold avelumab therapy pending clinical investigation	If irAE is ruled out, manage as appropriate according to the diagnosis and consider re-starting avelumab therapy<br>If irAE is confirmed, treat as Grade 2 or 3 irAE.
**Grade 2 irAE or first occurrence of Grade 3 irAE**	Withhold avelumab therapy<br>1.0 to 2.0 mg/kg/day prednisone or equivalent<br>Add prophylactic antibiotics for opportunistic infections<br>Specialty consult as appropriate	If improves to Grade ≤ 1:<br>Taper steroids over at least 1 month and resume avelumab therapy following steroids taper.
**Recurrence of same Grade 3 irAEs**	Permanently discontinue avelumab therapy<br>1.0 to 2.0 mg/kg/day<br>prednisone or equivalent<br>Add prophylactic antibiotics for opportunistic infections<br>Specialty consult as appropriate	If improves to Grade ≤ 1:<br>Taper steroids over at least 1 month.
**Grade 4**	Permanently discontinue avelumab therapy<br>1.0 to 2.0 mg/kg/day<br>prednisone or equivalent and/or other immunosuppressant as needed<br>Add prophylactic antibiotics for opportunistic infections<br>Specialty consult.	If improves to Grade ≤ 1:<br>Taper steroids over at least 1 month
**Requirement for 10 mg per day or greater prednisone or equivalent for more than 12 weeks for reasons other than hormonal replacement for adrenal insufficiency**<br><br>**Persistent Grade 2 or 3 irAE lasting 12 weeks or longer**	Permanently discontinue avelumab therapy<br>Specialty consult	

ADL=activities of daily living, ALT=alanine aminotransferase, AST=aspartate aminotransferase, CT=computed tomography; irAE=immune-related adverse event, IV=intravenous, LFT=liver function test, LLN=lower limit of normal, MRI=magnetic resonance imaging, NCI-CTCAE=National Cancer Institute-Common Terminology Criteria for Adverse Event, NSAIDs = nonsteroidal anti-inflammatory drugs, T4=free thyroxine, TSH=thyroid-stimulating hormone, ULN=upper limit of normal.
